# Adaptive Conductive Systems: Enabling Deformable Electronics Through Engineered Materials, Circuits, and Intelligent Systems

**DOI:** 10.1002/advs.77522

**Published:** 2026-09-21

**Authors:** Yufei Lu, Siyu Liang, Jingyi Niu, Jie Chen, Shuo Zhang, Hongjian Zhang, Dongsheng Jia, Wei Huang

**Affiliations:** ^1^ School of Flexible Electronics (SoFE) and Henan Institute of Flexible Electronics (HIFE) Henan University Zhengzhou China; ^2^ State Key Laboratory of Flexible Electronics (LoFE) Nanjing University of Posts & Telecommunications Nanjing China; ^3^ State IJR Center of Aerospace Design and Additive Manufacturing School of Mechanical Engineering Northwestern Polytechnical University Xi'an China; ^4^ State Key Laboratory of Flexible Electronics (LoFE) & Institute of Flexible Electronics (IFE) Northwestern Polytechnical University (NPU) Xi'an China

**Keywords:** adaptive conductive systems, circuit interconnection design, deformable conductors, flexible electronics, system integration

## Abstract

The rise of intelligent systems exacerbates scenario‐specific limitations in conventional rigid electronics, revealing critical compatibility gaps across emerging application fields such as wearable devices, bioelectronics, soft robotics, human–machine interfaces, etc. Adaptive conductive systems serve as critical enablers for next‐generation deformable electronics through engineering deformable conductors, innovating reconfigurable circuit topologies, and implementing intelligent systems, ensuring robust electrical connectivity and dynamic compliance in variable operating environments. This review presents the research advances of adaptive conductive systems from the perspective of material innovation, circuit interconnection design, system integration, and applications. Deformable conductors consist of substrates and conductive materials with their independent intrinsic properties and synergistic effects, structural designs and fabrication methods of conductive circuits with specialized stretchable configurations, as well as intelligent systems constructed via signal processing, machine learning, and coherent integration, are systematically summarized and reviewed. Finally, current challenges and future research perspectives of adaptive conductive systems are outlined, offering deep insights into research directions and strategies.

## Graphical abstract

This review presents the research advances of adaptive conductive systems from the perspective of materials, circuits, systems, and applications. Deformable conductors consist of substrates/matrices and conductive materials, structural design of circuit interconnections, fabrication of circuit interconnections, as well as intelligent systems constructed via signal processing, machine learning, and coherent integration are systematically summarized and reviewed.

## 1 Introduction

Driven by the development of the Internet of Things (IoT) and intelligent technologies, traditional rigid electronic devices are now confronting tremendous challenges in terms of adaptability and functionality. From wearable devices that conform to the human body, to soft robotics capable of adapting to complex environments, and to implantable monitoring devices, electronic technology is gradually overcoming the limitations of rigid materials by evolving toward flexible and deformable alternatives [1, 2]. Against this backdrop, adaptive conductive systems have emerged as a critical enabler for flexible electronic devices and systems due to their unique combination of electrical conductivity and mechanical flexibility or stretchability. Compared with rigid conductors, adaptive conductive systems can provide superior mechanical adaptability as well as maintain excellent electrical performance under continuous deformation, thereby enabling their application in dynamic environments. Specifically, the adaptability is systematically established across material, structural, and system levels. At the material level, they can actively adapt to environmental or mechanical stimuli through dynamic mechanisms such as dynamic reversible bonding or fluidic remodeling. At the structural level, engineered interconnect geometries effectively relieve mechanical stress, while at the system level, machine learning algorithms facilitate real‐time data adaptation and signal decoupling. Adaptive conductive systems have spurred rapid development of flexible and stretchable electronics in the field of wearable devices, bioelectronics, soft robotics, human–machine interfaces, etc., serving as a core component bridging materials science, electronic engineering, and biomedical disciplines [3].

The fundamental challenge of adaptive conductive systems lies in balancing electrical conductivity with mechanical performance, ensuring that stable electrical properties are maintained under significant mechanical deformation such as stretching, bending, or twisting. This objective imposes stringent demands on both the material system and structural design of the circuit. From the perspective of materials, substrate materials are enhanced via the introduction of dynamic bonding mechanisms [4, 5, 6] or through novel structural designs that endow them with self‐healing and reconfigurable functionalities. Simultaneously, the design of hierarchical or microstructures, or the employment of conductors with liquid state [7], ameliorates the inherent trade‐off between conductivity and stretchability of the conductive components. In terms of structural design of the circuit, configurations such as island‐bridge and curved layouts effectively mitigate strain concentration and thereby enhance the mechanical durability of adaptive conductive systems [8]. Despite these significant advances, several critical challenges remain to be addressed for adaptive conductive systems and their applications. First, current deformable conductors have not yet reached the theoretical limits of both metallic conductivity and polymeric deformability, particularly under extreme conditions (e.g., low or high temperatures, chemical corrosion, and high‐frequency dynamic loads) [9]. Second, existing manufacturing processes struggle to balance precision, cost, and scalability, thereby restricting large‐scale production and commercial applications [10, 11, 12]. Third, the long‐term stability and the integration of deformable conductors within flexible electronic systems require further optimization to meet the demands of diverse application scenarios.

In recent relevant reviews, although some related themes about deformable or stretchable conductors have been analyzed and discussed from different perspectives, there are still limitations such as the analytical targets remain narrowly constrained on the structural design, [13] or the applications are predominantly confined to a certain field (e.g., human–machine interfaces), [14] or the deformation mode is limited to tensile stretching, leaving other forms of deformation unexplored, [15] or the emphasis of the review is solely on the material aspect, lacking systematic discussion that spans from material design, to structural strategies, and to system integrations [16]. In comparison, this review offers a comprehensive analysis of materials categories and circuit design, encompassing a broader scope of diverse adaptive conductors. In addition, the system constitution of adaptive conductive systems spanning from signal processing, machine learning, to system integration is thoroughly reviewed, offering a summary and discussion from the perspectives of interdisciplinary research and multiple application fields.

As illustrated in Figure 1, this review systematically examines the research advances of adaptive conductive systems, establishing a multi‐tiered technical framework that spans material innovation, circuit interconnection design, system integration, and applications [13, 17, 18, 19]. To assist readers in navigating the extensive and rapidly expanding landscape of adaptive conductive systems, this comprehensive review is structured in a highly modular and logical sequence. Section 2 (Deformable Conductors) systematically examines the fundamental building blocks, detailing the selection criteria and mechanical interplay between flexible matrices/substrates and active conductive materials. Building upon these material foundations, Section 3 (Circuit Interconnections) explores the structural engineering of deformable interconnects and evaluates scalable fabrication techniques. Finally, Section 4 (System and Applications) shifts the focus toward macro‐level architectures. To articulate this evolution, the section follows a signal‐to‐application workflow, adopting a progressive logic that spans from the physical transmission of signals to intelligent data processing via machine learning algorithms [20, 21, 22, 23, 24]. This review places the emphasis on the synergistic interactions among materials, circuits, systems, and applications, summarizing current technical advances and challenges, proposing viable design strategies, and providing deep insights to facilitate the advancement of adaptive conductive systems in practical applications.

## 2 Deformable Conductors

Traditional conductive materials, such as metal films and rigid wires, typically suffer from cracking, delamination, or electrical failure under deformation. Their mechanical mismatch with soft substrates severely limits their application in deformable systems. To address this, researchers have developed a wide range of deformable conductors based on novel conductive materials. This section provides an analysis and overview of deformable conductors, focusing on two critical structural components: the supporting materials (substrates and matrices) and the conductive elements (fillers and coatings). This part introduces the selection and properties of various materials used as substrates or matrices, such as hydrogel, polydimethylsiloxane (PDMS), polyurethane (PU), polyethylene terephthalate (PET), and polyimide (PI), as well as carbon‐based materials, metals, and conductive polymers serving as conductive fillers or surface conductive layers, discussing their performance within deformable conductors.

To provide an overall understanding of different materials and their key characteristics, Table 1 summarizes various substrates or matrices, and conductive materials used in deformable conductors, along with their respective electrical and mechanical properties. By presenting a clear comparison, the table serves as a reference for the subsequent discussions on material selection, circuit interconnection design, and system integration.

**TABLE 1:** Comparison table of materials and properties of deformable conductors.

Substrate or matrix	Conductive material	Fabrication process/structure	Conductivity (S/cm)	Max strain (%)/twist	Number of cycles	Application	Reference
Hydrogel/VHB	CNT	Blending	0.01	1000/720°	1000	Wearable electronics	[25]
Hydrogel	Graphene	Freeze–thaw treatment	0.0035	366	2000	Wearable electronics	[26]
Hydrogel	AgNWs	Freezing and salting‐out treatments	1739	480	3000	Underwater equipment	[27]
Hydrogel/PAAc	PEDOT:PSS	Infiltration	2.35	399	10	Bio‐electronic Interfaces	[28]
Hydrogel/PU	PEDOT:PSS	Infiltration	11	400	5000	All‐hydrogel bioelectronic interfaces)	[29]
PDMS	CNFs	CVD	104	30	1000	Wearable sensors	[30]
PDMS	AgNWs	Shadow mask/Wave structure	8130	50	40	LED circuits	[31]
PDMS	PPy	Chemical polymerization	∖	70/90°	10,000	Flexible strain sensors	[32]
PDMS	Cr/Cu	Magnetron sputtering/Island bridge structure	596	8/76°	2000	Nanogenerator	[33]
PDMS	CNT	Laser cutting/Kirigami	7.66	430	5000	LED circuits	[34]
PDMS	CNT	Sedimentation/Kirigami	0.001	290	1000	Stretchable electrodes	[35]
PDMS	Au	Thermal evaporation/Crack structure	11 000	125	3000	Transparent stretchable circuit boards	[36]
PDMS	PS@Ag	Screen printing/Curved structure	165	80	30	Wearable electronics	[37]
PU	MXene	Spraying method	40	1600	10,000	Human activity monitoring	[38]
PU	Graphene	Paintability	12 400	1010	4000	LED circuits	[39]
PU	Au nanofilms	Sputtering/Curved structure	33 333	80	1000	Flexible strain sensors	[40]
PI	Pt	Ion‐sputtering	100	120°	5000	Human health monitoring	[41]
PI	Ag	Chemical silver coating/Curved structure	∖	61.5/360°	1000	Human activity monitoring	[42]
PET	Cu_x_S	Chemical bath method	793.65	83.3	200	Wearable electric heating devices	[43]
PET	Graphdiyne	Spin coating	5560	180°	1000	Photodetector	[44]
PET	CuNWs	Sedimentation	2.5×10^5^	∖	1500	Flexible transparent capacitive touchpad	[45]
PU/PDMS	Cu	Heat curing/Curved structure	160 000	100	∖	Strain sensors	[46]
TPU	Graphene	CVD/Wave structure	4.3	150	100	LED circuits	[47]
TPU	Graphene	Screen printing	1724.1	100	1000	Flexible circuits	[48]
TPU	CuGa LM	Fully solution‐based process/Island bridge structure	4.15 × 10^4^	300	1000	Flexible circuits	[49]
VHB acrylic elastomers	Graphene	Stamping transfer	∖	450/360°	50	Electrodes	[50]
Silicone rubber	CNTs	Blending	24 880	211	100	Strain sensors	[51]
Silicone Rubber	AuNW	Sedimentation/Curved structure	16 000	700	500	Stretchable electrodes	[52]
FG	AgNWs	Spraying method	64,300	∖	500	Electroluminescent device	[53]
SEBS	CNTs	Coating	38.3	1320	2000	Strain sensors	[54]
Ecoflex	AgNWs/CNTs	Solution filtration	370.4	460/540°	10,000	LED circuits	[55]
Ecoflex	GaIn LM	Screen printing/Island bridge structure	34 000	400	700	Battery array	[56]
Ecoflex	Ag/EGaIn LM	Screen printing/Island bridge structure	200 000	800	150	Biofuel cell	[57]
Polyurethane	AuNPs	Layer‐by‐layer deposition	11 000	486	10,000	Flexible electronics	[58]
Thermoplastic polymer	NiNPs	Fused filament fabrication	31 000	60	∖	Flexible electronics	[59]
Polyurethane sponge	EGaIn LM	Mechanical sintering	478	50	1000	LED circuits	[60]
SBS	EGaIn LM	Stencil printing/Curved structure	18 000	1800	100	LED circuits	[61]
∖	EGaIn LM/AgNW	Laser sintering/Curved structure	7090	800	10,000	LED circuits	[62]
SIS	PANI	Electrospinning	0.001	50	100	Flexible sensors	[63]
Fabrics	PEDOT:PSS	Wet‐spinning	10	55	1000	Flexible strain sensors	[64]
SEBS	PEDOT:PSS	Directly coated	4100	800	1000	LED circuits	[65]
	PEDOT:PSS/AgNFs	Electrospinning	47.17	∖	5000	LED circuits	[66]
Airlaid paper	Cu/Ni	Thermal adhesive tape/Origami	2870	30.4	1000	Flexible humidity sensors	[67]
PVA	EGaIn LM	Direct writing method/Kirigami	3100	105/720°	100	E‐skin	[68]
PUA	Pt	Sputter‐coating/Crack structure	106 000	2	10,000	E‐skin	[69]

*Note*: VHB, very high bond; CNT, carbon nanotube; AgNWs, silver nanowires; PAAc, polyacrylic acid; PDMS, Polydimethylsiloxane; CNFs, carbon nanofibers; CVD, chemical vapor deposition; PPy, polypyrrole; Cr, chromium; Cu, copper; Au, aurum; PS, polystyrene; PU, polyurethane; PI, polyimide; Pt, platinum; Ag, silver; PET, polyethylene terephthalate; CuNWs, copper nanowires; TPU, thermoplastic polyurethanes; Ga, gallium; AuNWs, gold nanowires; FG, fish gelatin; SEBS, styrene ethylene butylene styrene; GaIn LM, Eutectic Gallium‐Indium liquid metal; AuNPs, gold nanoparticles; NiNPs, nickel nanoparticles; SBS, styrene‐block‐butadiene‐block‐styrene; SIS, polystyrene‐block‐polyisoprene‐block‐polystyrene; PANI, polyaniline; PEDOT:PSS, poly(3,4‐ethylene dioxythiophene): polystyrene sulfonate; Ni, nickel; PVA, polyvinyl alcohol; PUA, polyurethane acrylate.

**FIGURE 1:**
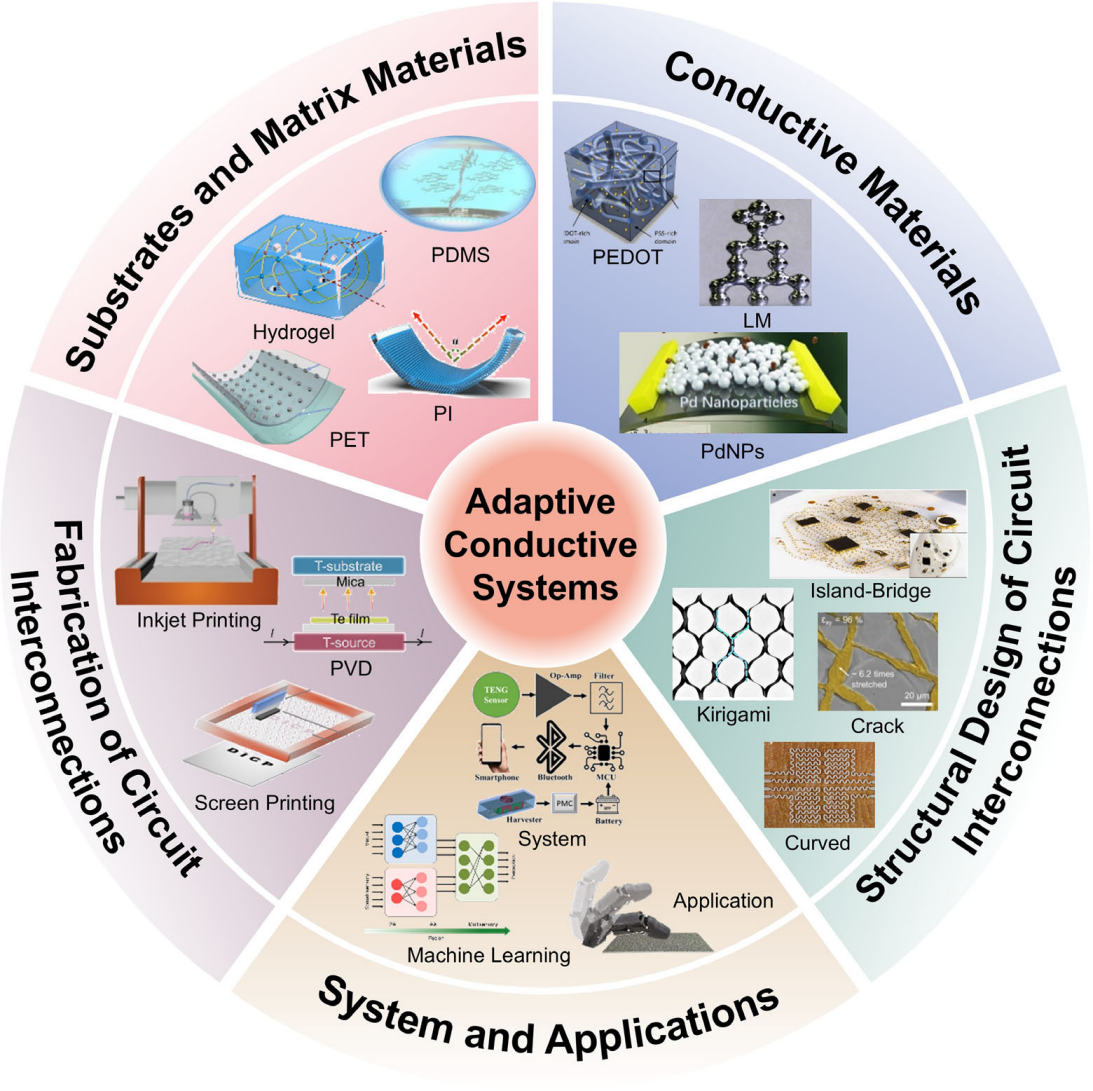
Schematic diagram of the adaptive conductive systems. This review focuses on the substrates and matrix materials, conductive materials, structural design of circuit interconnections, fabrication of circuit interconnections, and system and applications of adaptive conductive systems. PDMS image: Reproduced with permission [70]. Copyright 2024, American Chemical Society. Hydrogel image: Reproduced with permission [71]. Copyright 2023, Elsevier. PI image: Reproduced with permission [42]. Copyright 2021, Elsevier. PET image: Reproduced with permission [72]. Copyright 2018, Wiley‐VCH. Inkjet Printing image: Reproduced with permission [73]. Copyright 2025, Wiley‐VCH. PVD image: Reproduced with permission [74]. Copyright 2023, Wiley‐VCH. Screen Printing image: Reproduced with permission [75]. Copyright 2021, Elsevier. Machine Learning image: Reproduced with permission [76]. Copyright 2020, Springer Nature. System image: Reproduced with permission [77]. Copyright 2022, Wiley‐VCH. Application image: Reproduced with permission [78]. Copyright 2020, National Academy of Sciences. Island‐Bridge image: Reproduced with permission [79]. Copyright 2017, Springer Nature. Kirigami image: Reproduced with permission [68]. Copyright 2016, Wiley‐VCH. Crack image: Reproduced with permission [36]. Copyright 2021, Wiley‐VCH. Curved image: Reproduced with permission [80]. Copyright 2018, American Chemical Society. PEDOT image: Reproduced with permission [65]. Copyright 2017, American Association for the Advancement of Science. LM image: Reproduced with permission [81]/ Copyright 2013, Wiley‐VCH. PdNPs image: Reproduced with permission [82]. Copyright 2018, American Chemical Society.

### 2.1 Substrates and Matrix Materials

In the application of deformable conductors, the selection of the base materials is of paramount importance as it directly influences the flexibility, conductivity, stability, and overall performance of the system. Substrates provide solid, flexible foundations upon which conductive layers are coated, printed, or patterned. Polymers like PET and PI are predominantly utilized as traditional flexible substrates due to their thin‐film form and insulating nature. Comparatively, matrices encapsulate and embed conductive fillers to form a percolating composite network. For example, hydrogels are commonly used as matrices due to their excellent composition tunability and stretchability. Specifically, some versatile materials can either serve as substrate or matrix materials due to their unique components and structures, such as hydrogel, PDMS, and PU. Substrate and matrix materials should possess excellent mechanical properties, such as high flexibility and durability, as well as the ability to conform to complex geometries, ensuring proper adaptation to dynamic deformed surfaces. Moreover, biocompatibility and self‐healing capabilities are critical, particularly in biomedical device applications. This section provides a detailed discussion of several commonly used substrate and matrix materials, and by analyzing the characteristics and applications of these materials, theoretical guidance for selecting appropriate materials for deformable conductors is provided.

#### 2.1.1 Hydrogels

Hydrogels are commonly used elastomeric materials that serve as both a versatile substrate and a robust matrix for deformable conductors. Owing to their high‐water content (70–99 wt%), biocompatibility, and excellent flexibility and stretchability, hydrogel has been widely applied as a reliable structural platform for deformable conductors and soft bioelectronics [83]. Hydrogels exhibit superior mechanical compatibility with biological tissues compared to conventional rigid materials, particularly due to their ability to match the elastic modulus of biological tissues. Moreover, hydrogels possess appropriate softness and compliance, making them highly suitable for integration with biological systems [84, 85].

Through structural design and functional optimization of hydrogels, researchers have endowed them with enhanced mechanical properties, superior tear resistance, and self‐healing capabilities, thereby significantly broadening their application scope. Several studies have constructed double‐network structures [28, 86, 87] or employed physical crosslinking [88, 89] to impart superior stretchability and fatigue resistance. For example, Feig et al. developed a conductive interpenetrating network (IPN) hydrogel as a specific architecture of double‐network systems, successfully decoupling electrical and mechanical properties. This strategy enabled orthogonal control over the elastic modulus across three orders of magnitude (0.5–500 kPa) to mimic various biological tissues, providing a mechanically compliant matrix for soft bioelectronics (Figure 2a) [28]. Di et al. fabricated a purely physically cross‐linked conductive hydrogel through multiple non‐covalent interaction matching. By modulating the ratio of “soft” hydrophobic domains to “rigid” ionic coordination bonds, they achieved precise control over the structural and mechanical properties of the conductive matrix. The resulting hydrogel exhibited an ultrahigh fracture strain of over 2000%, a toughness of ∼60 MJ/m^3^, and a recovery ratio of 88.2%, providing a robust platform for high‐sensitivity motion detection and information transmission (Figure 2b) [88]. Such physical interactions facilitate the recover and reconstruct of the network of hydrogels during deformation (Figure 2c) [90, 91]. Furthermore, to enhance the stretchability of hydrogels, Jiang et al. developed a bacterial cellulose‐reinforced hydrogel that achieved a tensile strain of 2400% through hydrophobic associations, while exhibiting a fracture energy of up to 38 kJ/m^2^. When used as a stretchable matrix, the modified hydrogel demonstrated excellent mechanical stability under repeated cycles (1000 cycles at 30% strain), including its suitability for dynamic motion sensing applications [91]. The P(AA‐SMA)M hydrogel developed by Yang et al. exhibits reversible disruption and reformation of hydrophobic interactions upon mechanical damage, primarily attributed to the incorporation of hydrophobic long chains within the hydrogel network. As demonstrated in their study, the hydrogel matrix retained an elongation of up to 843% even after 20 fracture and self‐healing cycles (Figure 2d) [92]. Although the mechanical properties of hydrogels can be significantly improved through structural modulation, fatigue failure under repeated stretching cycles remains a critical challenge. Therefore, further investigation is required to develop strategies that enhance their resistance to mechanical fatigue.

**FIGURE 2:**
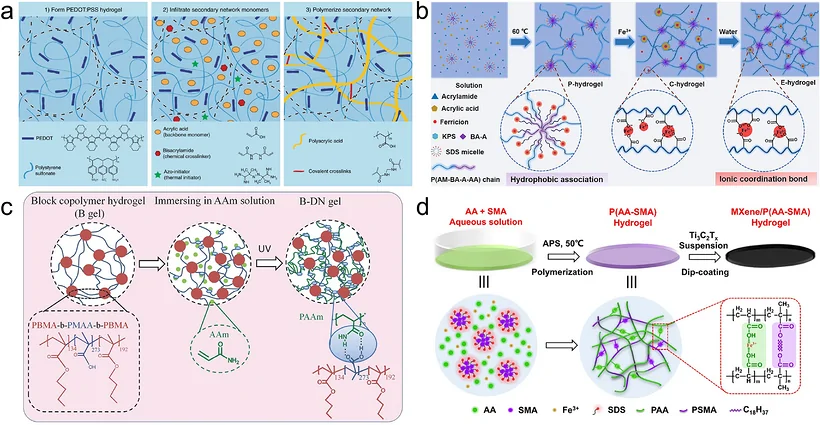
Functionalised construction of hydrogel structures. (a) Double‐network structure hydrogel. Reproduced with permission [28]. Copyright 2018, Springer Nature. (b) Schematic of the preparation process of double‐network dynamic cross‐linked hydrogels. Reproduced with permission [88]. Copyright 2022, Royal Society of Chemistry. (c) Structure of block copolymer gel (B gel), the synthesis procedure of the double‐network gel (B‐DN gel), and the structure of B‐DN gel with hydrogen bonds. Reproduced with permission [91]. Copyright 2016, Wiley‐VCH. (d) Schematic illustration of the fabrication of the hydrogel and the corresponding interactions in hydrogel polymer networks. Reproduced with permission [92]. Copyright 2023, American Chemical Society.

The self‐healing capability [93] is a critical evaluation metric for hydrogels used as flexible substrates or matrices, primarily relying on dynamically cross‐linked networks. These networks enable rapid recovery through non‐covalent bonds (e.g., hydrogen bonding [94, 95] and hydrophobic interactions [92, 96] or reversible covalent bonds, such as disulfide bonds [6] and Diels–Alder reactions (Figure 3a) [97, 98]. For instance, Cai et al. developed a dynamically cross‐linked hydrogel composed of polyvinyl alcohol (PVA) and borate, primarily facilitated by reversible borate ester bonds and supplemental hydrogen bonding, which demonstrated a recovery of 99% in electrical conductivity after damage. This hydrogel exhibits reversible cross‐linking and dissociation capabilities at room temperature, enabling the matrix to self‐repair under mechanical deformation [25]. The hydrogel composed of xanthan gum and montmorillonite demonstrates a high self‐healing efficiency of up to 95% through hydrophobic interactions, hydrogen bonds, and ionic coordination interactions with COO^−^ groups [5]. In addition to its fatigue resistance and reusability, this material presents valuable implications for the design of deformable conductors requiring repeated mechanical deformation. In another study, a supramolecular hydrogel based on hydrogen bonding achieved both high mechanical strength (1.1 MPa) and a self‐healing efficiency of approximately 95% by facilitating dynamic reorganization of multiple hydrogen bonds. The double‐network structure combines hydrogen‐bonding networks with physically cross‐linked networks in a synergistic manner, imparting the hydrogel with remarkable shape memory behavior and dynamic recovery capability [99]. Bi et al. developed a PVA double‐network hydrogel using an alkali solution system and physical cross‐linking via multiple hydrogen bonding. The resulting hydrogel exhibited exceptional mechanical performance, achieving a tensile strength of 2.1 MPa and a fracture energy of 13.5 kJ/m^2^. Furthermore, the material demonstrated a low equilibrium swelling ratio and superior biocompatibility with no cytotoxicity, highlighting its potential for tissue engineering and implantable medical applications [86]. An innovative strategy involves the introducing of covalent‐like hydrogen‐bonding network repair mechanisms, such as the addition of trehalose to enhance the environmental stress resistance of hydrogels. Trehalose molecules form strong hydrogen bonds with polar groups in polyacrylamide chains, which not only significantly improves the mechanical resilience and extensibility of the hydrogel but also maintains excellent mechanical stability under extreme temperatures and arid conditions. This approach effectively mitigates the mechanical collapse typically observed in conventional hydrogels, providing a versatile platform for robust soft electronics in harsh environments [100]. In practical applications, self‐healing hydrogels exhibit outstanding environmental adaptability and long‐term durability. For instance, ionic hydrogels form fully reversible network structures through dynamic hydrogen bonding and ionic coordination, which not only enhance their intrinsic self‐healing capabilities but also confer ultraviolet filtering properties and environmental stability. These hydrogels maintain effective moisture retention even under cryogenic conditions. Such multifunctional composite ionic hydrogels demonstrate significant potential for use in human health monitoring and smart wearable electronic devices [101]. Moreover, by combining deep eutectic solvents with cellulose nanocrystals, hydrogels formed through ionic and physical cross‐linking exhibit a dynamic network that achieves exceptional self‐healing performance and water solubility. This water‐soluble feature significantly mitigates environmental impact from electronic waste, making such hydrogels particularly suitable for eco‐friendly applications requiring biodegradable sensors [102]. Despite notable progress in improving the self‐healing capabilities of hydrogels, further optimization of their performance stability under prolonged and repeated cyclic deformation remains essential. For instance, repeated healing cycles may lead to a gradual decline in mechanical strength, which poses a significant challenge that must be addressed in future research. Moreover, to meet the demands of complex application scenarios, it is imperative to develop novel self‐healing hydrogel‐based materials with enhanced multifunctionality.

**FIGURE 3:**
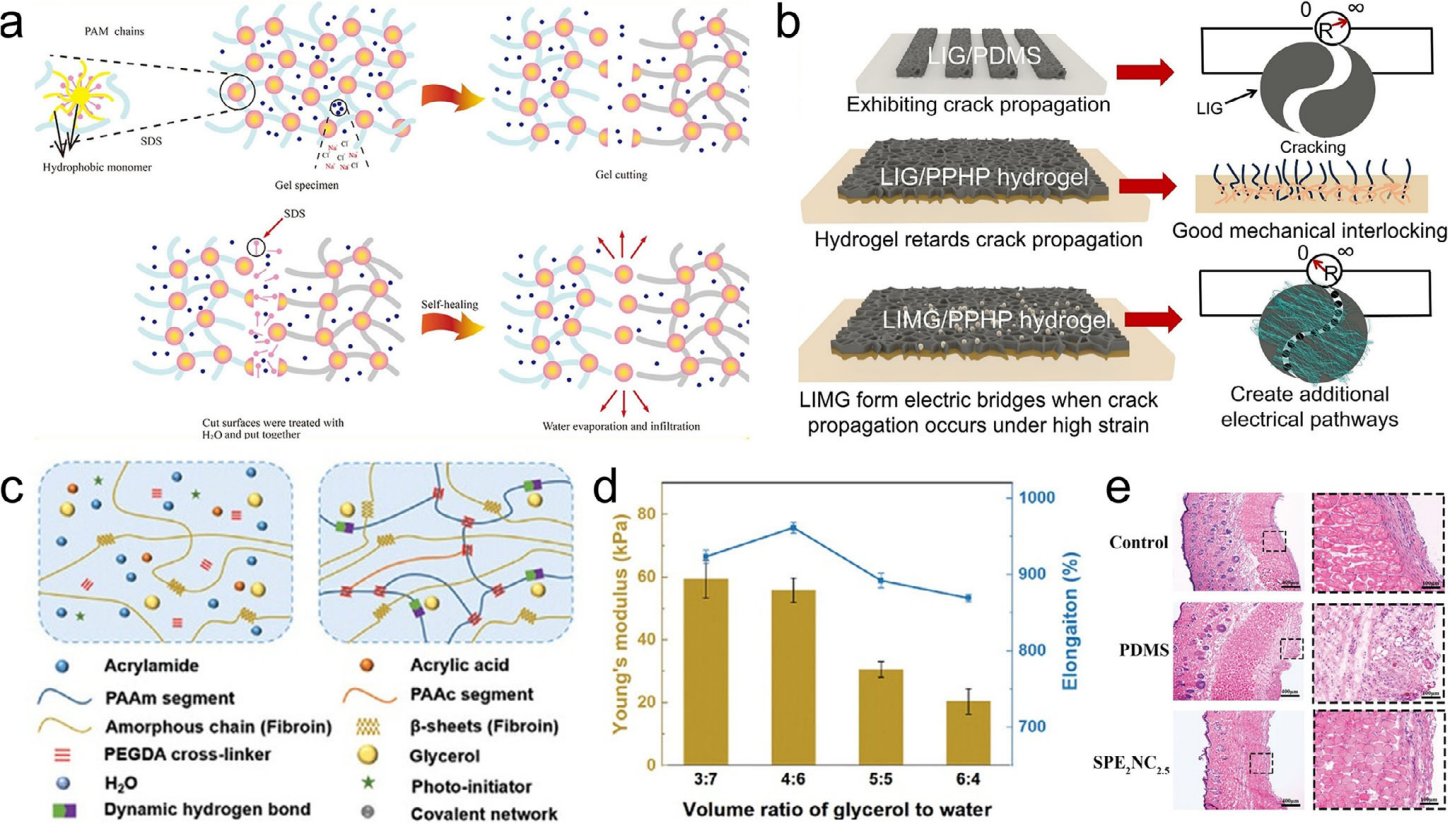
Functionalised hydrogel matrix. (a) Schematic representation of the self‐healing mechanism of the hydrogel. Reproduced with permission [98]. Copyright 2016, American Chemical Society. (b) The mechanisms by which electrical stability is maintained in hydrogel. Reproduced with permission [26]. Copyright 2024, Elsevier. (c) Schematic of the cross‐linking network. Reproduced with permission [107]. Copyright 2024, Wiley‐VCH. (d) The performance of hydrogels under different glycerol concentrations. Reproduced with permission [107]. Copyright 2024, Wiley‐VCH. (e) The biocompatibility of hydrogels. Reproduced with permission [111]. Copyright 2023, Wiley‐VCH.

Electrical conductivity is another essential property of hydrogels in flexible electronics. The incorporating of conductive materials such as graphene, carbon nanotubes (CNTs), or polyaniline (PANI) can significantly enhance their electrical performance. The conduction of ionically conductive hydrogels originates from ion migration within the hydrogel network. The addition of ionic dopants (e.g., NaCl, KCl, CaCl_3_) significantly improves ionic conductivity. For instance, Cheng et al. developed an organohydrogel with a conductivity of 1.25 S/m, which exhibits high sensitivity to mechanical deformation and demonstrates outstanding performance in dynamic monitoring and health management applications [103]. As another example, the composite material combining bilayer graphene with an ionic hydrogel demonstrates the ability to maintain a linear electrical response under high‐strain conditions (exceeding 200%), while also exhibiting effective humidity management functionality (Figure 3b) [26, 104]. However, ionic hydrogels face stability challenges, as dissolved ions may induce electrode corrosion and gradually compromise mechanical integrity. Beyond ionically conductive hydrogels, researchers have developed electronically conductive hydrogels with exceptional performance. For example, a freeze‐salting‐out method was employed to embed into a PVA matrix, resulting in a hierarchically structured conductive hydrogel with ultrahigh conductivity of 1739 S/cm, while maintaining a high water content of 87% and a stretchability of 480% [27]. The hierarchical structure consisting of a cationic polyacrylamide network and lantern arene‐based hydrogen‐bonding organic framework (HOF) nanofibers, enhances the continuity of conductive pathways while providing exceptional mechanical strength. Unlike conventional soft hydrogels, this design achieves a high Young's modulus of 31.9 MPa and a toughness of 6.97 MJ/m^3^ without sacrificing stretchability. Nonetheless, the trade‐off between mechanical properties and electrical conductivity remains a major challenge in material development. Double‐network hydrogels and multilayer composite architectures have been widely used to balance these characteristics [105]. For example, Ren et al. fabricated a sandwich‐structured hydrogel by embedding a flexible interlayer between two conductive CNTs layers, achieving a conductivity of 25 S/m while maintaining high stretchability with a maximum strain of 298% [106].

Water loss is a critical limiting factor for hydrogels in practical applications. To address this issue, researchers have developed various strategies to enhance water retention. For example, the incorporation of hydrophilic components (e.g., glycerol, polyethylene glycol (PEG) or the design of high‐water‐content double‐network structures can significantly mitigate water evaporation, improving the performance stability of hydrogels under dry or harsh conditions. Wu et al. demonstrated that adding glycerol reduces the saturated vapor pressure of hydrogels, thereby enhancing their water‐retention capacity. This modification enables long‐term usability, with the hydrogel maintaining robust mechanical and adhesive performance even after 10 days of storage (Figure 3c,d) [107, 108]. Zhuo et al. enhanced the dehydration resistance of hydrogels by incorporating a glycerol‐water binary system, achieving storage stability of more than 30 days [109]. Similarly, Ge et al. achieved simultaneous cryoprotection and high water retention in hydrogels through a dual‐solvent strategy based on glycerol and water [110].

Hydrogels have emerged as ideal platform materials for implantable devices and neural interfaces owing to their biocompatibility and mechanical flexibility. For example, a hydrogel based on PEG‐modified silk fibroin combined high conductivity with optical transparency, providing a reliable dual‐modal interface for monitoring neurovascular activity. This material exhibits excellent in vivo stability and low impedance, enabling long‐term efficient signal transmission [98]. Zhang et al. conducted a comprehensive series of analyses, including protein adsorption resistance tests and cell compatibility experiments. The results demonstrated that the hydrogel significantly reduced nonspecific protein adsorption, supported high cell viability, and induced minimal inflammatory responses under physiological conditions, conclusively establishing its biocompatibility (Figure 3e) [111]. Fu et al. evaluated the biocompatibility of hydrogels using mouse epithelial‐like fibroblasts and demonstrated that the hydrogels induced negligible cell death [71]. These findings collectively indicate that stretchable and biocompatible hydrogels possess substantial potential as matrix materials for flexible biomedical devices.

In summary, hydrogels have emerged as a pivotal material for deformable conductors due to their integrated performance in conductivity, mechanical flexibility, and biocompatibility. However, there remain several critical challenges, including conductivity degradation in dry conditions, the inherent trade‐off between the content of conductive fillers and mechanical robustness, and the difficulty of maintaining long‐term functional stability. Future research should focus on developing multifunctional hydrogel‐based elastic platforms with enhanced environmental adaptability. This goal may be achieved by leveraging dynamic cross‐linking strategies, optimizing composite structures, or employing innovative methods to simultaneously enhance tear resistance, self‐healing performance, water retention, and biocompatibility.

#### 2.1.2 Polydimethylsiloxane

Polydimethylsiloxane (PDMS), a highly flexible silicone elastomer, has been widely employed in flexible electronics and sensors applications. As a classical elastic material, PDMS exhibits low Young's modulus, excellent biocompatibility, and remarkable chemical stability [112, 113]. These characteristics make it an ideal substrate material for various flexible and wearable electronic devices [114].

With its superior flexibility and optical clarity, PDMS serves as an ideal substrate for deformable conductors, enabling stable integration with complex surfaces and enhancing the mechanical and sensing performance of the overall device. To further improve sensitivity, various surface micro‐architectures, such as micropillars [115, 116, 117], interlocking structures [118], microdomes [119, 120], and pyramids [121, 122], have been patterned onto PDMS substrates. By modulating the compression and deformation processes of the multistage structure composed of PDMS microstructures, the contact area inside the sensing layer was altered when pressure was applied, which significantly modified the resistance of the flexible sensors [123]. Furthermore, the excellent flexibility and mechanical stability of PDMS enable conformal adhere to curved biological surfaces or device surfaces, ensuring stable electrical conductivity even after tens of thousands of cycles of stretching and bending [124]. In the design of bioinspired, PDMS serves as a critical flexible substrate, providing an ideal mechanical support platform for the integration of graphene‐PDMS composite microsphere sensing layers. Experimental data indicate that the superior elasticity of PDMS ensures the structural integrity of the device even at a fracture elongation of 70%. Furthermore, the PDMS‐based sensor exhibited exceptional mechanical stability and signal repeatability over more than 2000 loading cycles, demonstrating its significant potential for applications in electronic skin and long‐term health monitoring [114].

The enhancement of self‐healing capability has become an important research focus for PDMS elastic substrates. By incorporating reversible chemical bonds such as imine bonds (Figure 4a) [70, 125, 126], coordination bonds (Figure 4b) [127], hydrogen bonds [128], and disulfide bonds [129]. PDMS‐based materials can achieve effective autonomous repair, thereby expanding their application potential and service life. A representative study by Sun's team demonstrated that introducing reversible imine bonds into PDMS enables flexible sensors to autonomously repair minor damage. This self‐healing capability significantly improves device longevity and operational safety, particularly for long‐term wearable health monitoring applications [70]. By integrating sliding crosslinks (polyrotaxanes) and dynamic hydrogen bonding into the PDMS, the material achieves a high fracture strength of 1.05 MPa and an exceptional stretchability of 2800%. The synergistic effect of the movable crosslinkers and dynamic hydrogen bonds enables efficient energy dissipation and confers the material with robust mechanical properties. Furthermore, the elastomer exhibits a remarkable thermally induced self‐healing capability, recovering 93% of its original strain after 48 h at 55°C (Figure 4c) [130]. The dynamic nature of disulfide bonds introduced into the PDMS substrate by Guo et al. is significant to enabling self‐healing behavior. These bonds possess dynamic exchange capabilities, allowing spontaneous bond cleavage and reformation even at room temperature, without the need for external energy input (Figure 4d) [131]. However, self‐healing PDMS still exhibits lower mechanical strength compared to traditional materials such as plastics and rubbers. Besides, performance degradation may also occur during repeated healing cycles. Therefore, achieving an optimal balance between self‐healing capability and overall mechanical performance remains a key challenge requiring further investigation.

**FIGURE 4:**
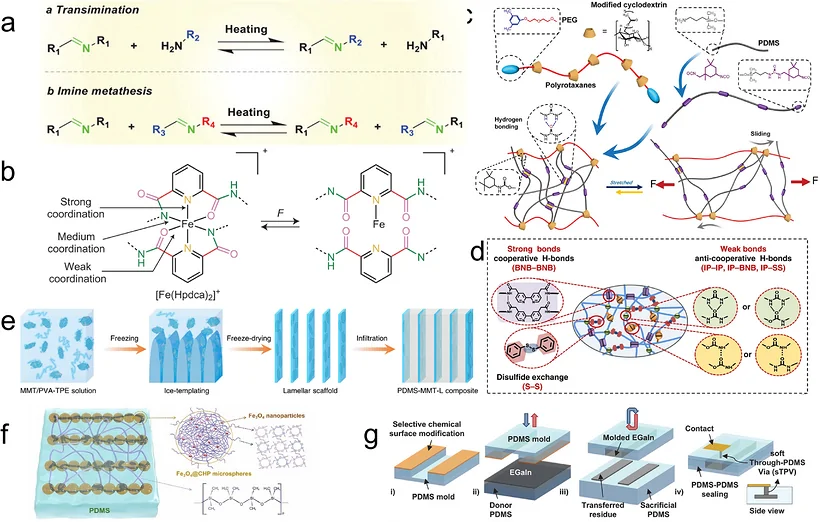
Functionalized PDMS substrate or matrix. (a) The network containing imine bonds combines the dynamic properties of supramolecular polymers and the structural stability of covalent networks. Reproduced with permission [126]. Copyright 2023, Wiley‐VCH. (b) The combination of strong and weak bonding sites in a single ligand provides both strong crosslinking and energy dissipation mechanisms for strain. Reproduced with permission [127]. Copyright 2016, Nature. (c) A schematic depiction of elastomer crosslinked by polyrotaxanes and hydrogen bonding. Reproduced with permission [130]. Copyright 2020, Wiley‐VCH. (d) These dynamic bonds are introduced in a PDMS polymer backbone, spontaneously forming a dynamic supramolecular polymer network. Reproduced with permission [131]. Copyright 2020, Springer Nature. (e) PDMS incorporating nanomaterials. Reproduced with permission [134]. Copyright 2021, Springer Nature. (f) Aligned CHP microspheres are incorporated into the PDMS matrix. Reproduced with permission [136]. Copyright 2023, Springer Elsevier. (g) Schematic diagram of the proposed advanced liquid metal patterning process based on soft lithography for scalable. Reproduced with permission [137]. Copyright 2017, Wiley‐VCH.

To enhance the toughness and tear resistance of PDMS elastic substrates, researchers commonly incorporate reinforcing materials such as nanomaterials [132, 133, 134] and liquid metals [135]. To overcome the limitations of low stiffness and toughness in conventional silicones, Peng et al. employed an ice‐templating technique to fabricate a nacre‐like PDMS‐MMT nanocomposite. This approach resulted in a nacre‐inspired layered nanocomposite that achieved a 23‐fold increase in Young's modulus and a 12‐fold improvement in toughness compared to pure PDMS, providing a stiff yet tough substrate platform for advanced flexible devices (Figure 4e) [134]. To enhance the mechanical robustness of PDMS substrates, Tao et al. proposed a toughening strategy by incorporating cyclosiloxane hybrid polymer (CHP) microspheres into the PDMS matrix, a “homogeneous yet heterostructured” design that significantly improves the fracture toughness of the resulting substrate while preserving excellent processability. These high cross‐linking density CHP microspheres, synthesized via emulsion polymerization, exhibit inherent chemical compatibility with PDMS and facilitate robust adhesion through interfacial polymer chain interpenetration. Consequently, the modulus contrast between the microspheres and the PDMS matrix induces significant pinning and deflection effects during crack propagation. With a fracture toughness reaching 4223 J/m^2^, this composite offers a high‐performance solution for encapsulation and substrates in the advancement of long‐life flexible electronics (Figure 4f) [136]. From a structural engineering perspective, Kim et al. demonstrated that embedding liquid‐metal microchannels within a PDMS matrix can significantly enhance the system's mechanical endurance. Under external deformations, these fluidic micro‐networks structurally adapt to applied stresses without localized tearing or rupturing, contributing to outstanding overall mechanical resilience and functional reliability (Figure 4g) [137]. The PDMS‐MMT composite demonstrated crack resistance under stress concentration, making it suitable for high‐stress flexible electronics. However, such additives may reduce optical transparency, potentially limiting applications in optoelectronic devices. In a macroscopic fiber design, Li et al. developed a strategy embedding stretchable hard fibers within the soft PDMS matrix. In this design, the hard fibers initially arrest crack propagation. When cracks eventually sever the fibers, the stored elastic energy dissipates dramatically, achieving exceptional tear resistance exceeding 1000 J/m^2^ [22, 138]. This fiber‐reinforced structure enables PDMS composites to rival natural rubber in toughness. Nevertheless, interfacial debonding between fibers and the PDMS elastic substrate can induce atypical crack deflection phenomena. Achieving simultaneous enhancement of both elastic modulus and fracture resistance in PDMS substrates to match the performance benchmarks of natural soft materials (e.g., human skin or animal leather) remains an unresolved challenge in materials engineering [134].

In biomedical applications, PDMS has garnered significant attention due to its excellent biocompatibility and chemical inertness. When in contact with human tissues, PDMS does not induce immune responses and exhibits strong resistance to chemical degradation, making it widely used as an encapsulation material for implantable sensors and microfluidic devices [139]. Moreover, the inherent optical transparency and gas permeability of PDMS further enhance its suitability for biomedical applications, particularly in wearable devices and in vitro diagnostic applications. It is extensively used as an elastic substrate in areas such as human motion monitoring [37, 140, 141, 142], eye movement control [143], bone regeneration [144, 145, 146], and electronic skin [147, 148, 149]. For instance, Tang et al. combined PDMS with aluminum nitride to create a piezoelectric thin‐film substrate. This film generates sustained alternating current under mechanical vibration, simulating the natural electrical potential gradient in bone tissue, thereby significantly promoting osteoblast proliferation and osteogenic gene expression. Compared to traditional electroactive substrate materials, this PDMS‐based piezoelectric film offers superior processability, flexibility, and biocompatibility, breaking the limitations of rigid materials in bone regeneration [146]. However, the inherent hydrophobicity of PDMS surfaces may lead to insufficient affinity in certain biological applications. To improve biocompatibility, some studies have attempted surface modifications or plasma treatments [150] to enhance the hydrophilicity of PDMS elastic substrates, better supporting cell culture and tissue engineering applications [151]. Wolf's study revealed that the hydrophobicity of PDMS restricts its adhesion to skin surfaces, leading to reduced comfort during prolonged wear [152]. To further improve its skin contact, researchers typically apply hydrophilic modifications to PDMS, including plasma treatment [153], silanization [154], and chemical grafting [155], thereby enhancing its skin conformability [156, 157].

PDMS is extensively utilized as a substrate for wearable health monitoring due to its biocompatibility and skin‐like mechanical properties, featuring a low Young's modulus (typically 0.5–3 MPa) that ensures seamless modulus matching with human tissue. When integrated with conductive fillers such as silver nanowires (AgNWs), carbon nanotubes (CNTs), or carbon fiber cloth, PDMS‐based deformable conductors enable high‐resolution monitoring of physiological signals including pulse waves, vocal vibrations, and joint movements, while maintaining long‐term stability under repeated mechanical deformation [148]. For example, Verma et al. embedded carbon fiber cloth into a PDMS substrate to create a highly sensitive nanocomposite membrane. The resulting strain sensor demonstrated a high electrical conductivity (1.65 × 10^4^ S/m), excellent stability (2000 cycles), and strong tolerance to environmental variations ranging from 5°C to 45°C, the resistance change also remains essentially constant over a humidity change of 40% to 85% [140]. When applied to monitoring dynamic moving of body regions such as the forearm and knee, the PDMS‐based substrate exhibited excellent sensitivity and signal reproducibility, offering an ideal solution for health monitoring and biomechanical studies [37]. Wang et al. developed a PDMS‐based flexible sensor for tracking eye movements, providing disabled patients with intuitive control of assistive devices (e. g., wheelchairs). Unlike conventional rigid metal electrodes, the breathable PDMS substrate minimizes allergic reactions and skin irritation, enhancing the comfort and safety for wearers. With a low Young's modulus of 296 kPa, the deformable conductor closely conforms to curved anatomical surfaces such as the forehead without causing pressure‐related discomfort during extended wear [143]. Furthermore, since PDMS lacks inherent antibacterial properties, various functionalization strategies have been employed to enhance its suitability for medical applications [158]. For instance, Hu et al. developed a PDMS elastic substrate incorporating polydopamine‐modified gallium (Ga) nanodroplets, which demonstrated significantly improved antibacterial performance [159]. Although PDMS holds considerable promise for biomedical applications, its use is constrained by inherent hydrophobicity, limited surface adhesion, and inadequate intrinsic electrical conductivity. Researchers can address these limitations by employing chemical modification and composite material strategies to expand the range of potential applications in the future [152].

In conclusion, PDMS demonstrates significant potential as an elastomeric substrate material for flexible electronics and biomedical devices. Through tailoring composite materials and structural designs, the conductivity, toughness, self‐healing capability, and biocompatibility of PDMS‐based substrates have been markedly enhanced, enabling diverse applications in flexible sensors, electronic skin, and wearable electronics. However, persistent challenges remain regarding the lack of intrinsic conductivity and extreme surface hydrophobicity. Future research should focus on developing novel composite materials and functionalization strategies to optimize the multifunctional performance of PDMS, addressing the increasingly complex demands in both practical applications and fundamental studies.

#### 2.1.3 Polyurethane

Polyurethane (PU), as an elastic substrate or matrix material, has demonstrated significant application potential in flexible electronics due to its excellent mechanical flexibility and good compatibility with various conductive fillers. By incorporating conductive materials such as graphene [160], AgNWs [161], and liquid metals (Figure 5a) [162], PU‐based elastomer materials can simultaneously achieve high stretchability and superior electrical conductivity, making them ideal candidates for deformable conductors in flexible sensors and wearable electronic devices.

**FIGURE 5:**
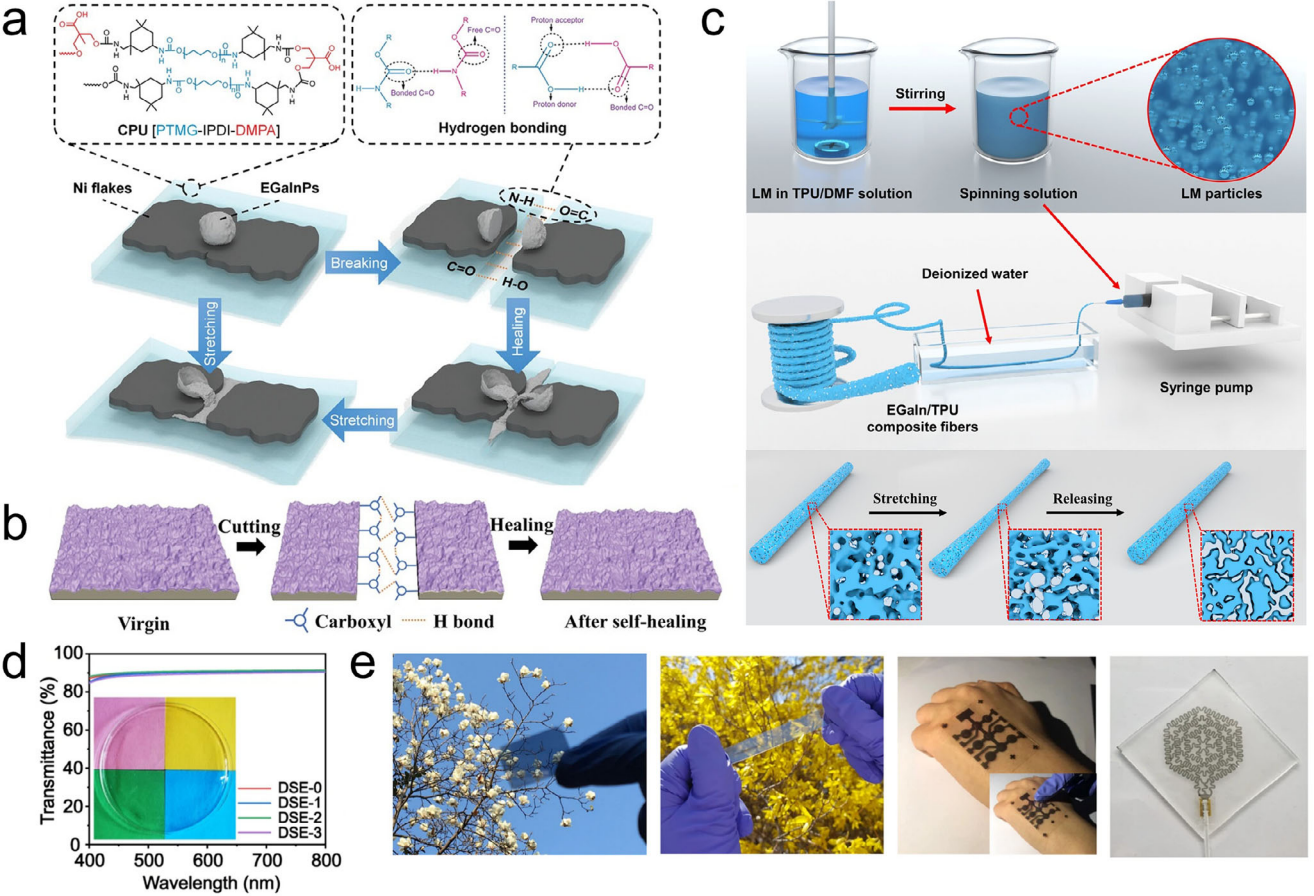
Functionalized PU substrate or matrix. (a) Schematics of the mechanism for mechanically and electrically stretchable and self‐healing properties of the Ni flakes‐EGaInPs‐CPU conductor. Reproduced with permission [162]. Copyright 2019, Wiley‐VCH. (b) Schematic diagram of the self‐healing mechanism of the sensitive layer. Reproduced with permission [38]. Copyright 2022, Wiley‐VCH. (c) Schematic diagram of the PU 3D structure. Reproduced with permission [164]. Copyright 2013, American Chemical Society. (d) Schematic diagram of transparency. Reproduced with permission [166]. Copyright 2022, Springer Nature. (e) Schematic diagram of stretchable and semi‐transparent. Reproduced with permission [167]. Copyright 2021, Springer Nature.

The molecular structure of PU features a combination of soft and hard segments, which provides an effective balance between elasticity and mechanical strength. This makes it an excellent platform for the development of composite materials. For example, Gao et al. synthesized a graphene‐enhanced PU matrix that exhibits both high stretchability and notable self‐healing capability. By utilizing dynamic bonding mechanisms, the graphene‐PU composite achieved efficient self‐healing property at room temperature, restoring ∼90% of its electrical conductivity within 10 min. This self‐healing property renders PU composites highly suitable for applications such as wearable sensors and flexible electronic circuits [160]. PU, due to its inherent elasticity and structural versatility, has been widely employed as a substrate material in high‐performance flexible pressure sensors. Yang et al. fabricated high performance flexible pressure sensors using PU as the substrate material. Their work reported the tensile properties of PU in both pristine and self‐healed states, exhibiting the pristine material achieving strains of up to 1600%, while the self‐healing material maintains strains exceeding 1400% (Figure 5b) [38]. Li et al. designed a flexible sensor using a PU sponge as the elastic matrix. The sponge exhibited high porosity, approximately 95%, along with excellent mechanical resilience. It was capable of withstanding substantial deformations, including compression, bending, stretching, shearing, and twisting, without structural failure, thereby demonstrating exceptional flexibility [163]. These examples underscore the structural adaptability and mechanical robustness of PU‐based substrate or matrix in adaptive conductive systems, particularly under complex mechanical loading conditions.

The incorporation of three‐dimensional (3D) structures can significantly enhance the performance of PU as a stretchable substrate or matrix. For example, a 3D structure constructed from polydopamine‐modified PU sponge served as an effective scaffold for liquid metal conductors, resulting in composite materials with high conductivity of 478 S/cm, and the conductor maintained stable electrical pathways even under extreme deformation, with the relative resistance increasing only 2.2 times at 400% strain. These properties make such a composite particularly well‐suited for applications in stretchable LED arrays and other flexible electronic devices [60]. Similarly, the integration of wavy graphene foam with thermoplastic polyurethane (TPU) enabled the construction of a hierarchical conductive network. It maintains excellent electrical conductivity (4.3 S/cm) with minimal resistance change (only a 22% increase in resistance) even at 150% strain, ensuring stable performance under cyclic deformation. The composite demonstrates consistent electrical resistance during repeated stretching up to 150% strain and twisting up to 720° [47]. The three‐dimensional (3D) structure of PU substrates is primarily manifested through their porosity, dynamic cross‐linked networks, and helical configurations. As a thermoplastic polyurethane, TPU contains both soft and hard segments in its molecular architecture. This unique molecular design allows the hydrogen bonds to dissociate upon heating, rendering the polymer chains mobile. Upon cooling, the hydrogen bonds reform, and the chains re‐crosslink to establish a new 3D network structure. This programmability offers significant flexibility in designing deformable conductor substrates (Figure 5c) [164].

Furthermore, PU demonstrates excellent compatibility as a stretchable substrate. For instance, PU substrates exhibit high optical transparency, making them suitable for applications requiring transparent or semi‐transparent properties. They also show superior processing compatibility and can be readily integrated with conductive layers through simple fabrication techniques (e.g., coating, spraying), thereby reducing manufacturing complexity and cost. Huang et al. deposited nanocomposite polycaprolactone‐gelatin (PCLG) nanofibers (NFs) onto the PU substrates through electrospinning, forming a PU/NF composite membrane. A gold layer was subsequently deposited on the composite membrane via magnetron sputtering to enhance conductivity. Owing to the high elasticity of the PU substrate and its mechanical compatibility with NFs, the resulting PU/NF‐based deformable conductors achieved a low detection limit of 0.1% and a fast response time within 0.15 s when used as strain sensors, enabling the monitoring of subtle physiological activities such as wrist bending and swallowing [165]. Dynamic Supramolecular Elastomer (DSE) can be regarded as a specialized form of PU material. As studied by Chen et al., its microphase‐separated structure enables a transmittance exceeding 90% in the visible light range, demonstrating excellent optical transparency. Building upon the PU framework, DSE incorporates dynamic supramolecular chemical bonds, which endow the material with enhanced self‐healing ability, transparency, and flexibility (Figure 5d) [166]. Yoon et al. employed polyurethane (PU) (20 nm thick) as a compliant substrate to fabricate semitransparent and stretchable Ag‐polytetrafluoroethylene (PTFE) (140 µm thick) hybrid conductors via a co‐sputtering process. The system achieved a low sheet resistance of 3.09–17.23 Ω/sq alongside an optical transmittance of 25.27%–38.49% at 550 nm. Furthermore, the conductor exhibited exceptional mechanical reliability, withstanding up to 20% tensile strain, achieving stable electrical resistance over 1000 bending cycles at a bending radius of 1 mm, while simultaneously providing hydrophobic self‐cleaning properties. These synergistic optoelectronic and mechanical characteristics position the Ag‐PTFE/PU platform as a promising candidate for wearable self‐cleaning sensors, touch panels, and electrophysiological monitoring systems (Figure 5e) [167].

In summary, although PU has been widely utilized as a stretchable substrate or matrix in flexible electronics, several challenges still remain, including optimizing the dispersion of conductive fillers and maintaining the integrity of conductive networks under mechanical strain. Furthermore, achieving a balance between high stretchability and environmental stability requires continued innovation in material design and processing strategies. Addressing these challenges will further establish PU as a versatile and high‐performance material for next‐generation flexible and wearable electronic devices.

#### 2.1.4 Polyethylene Terephthalate

Polyethylene terephthalate (PET) is a cost‐effective insulating substrate, making it an ideal support for transparent conductive electrodes. It exhibits superior optical transparency, excellent processability, and mechanical flexibility. Although it has limitations such as low heat resistance and limited flexibility without inherent stretchability, these drawbacks can be mitigated through functional doping and material modification, which may also introduce additional functionalities [168].

By leveraging the excellent dimensional stability of PET, researchers have developed various high‐performance conductive composites. For example, Zhang et al. employed a chemical bath deposition method to uniformly coat an insulating PET substrate with a continuous Cu_x_S film embedded with abundant Cu_x_S nanospheres. The resulting composite integrates dual‐mode electrothermal and photothermal heating functionalities. By carefully engineering the hierarchical nanostructure, the Cu_x_S‐coated PET composite exhibits electrothermal and photothermal conversion performance, achieving a low sheet resistance of 1.26 Ω/sq and a maximum surface temperature of up to 172°C. It is worth noting that the commercial PET fabric used here consists of highly semi‐crystalline fibers that are industrially heat‐set. These features underscore its strong potential for all‐weather wearable thermal management applications. In this study, PET is utilized as an insulating substrate that offers both mechanical flexibility and chemical stability. Through the incorporation of nano‐embedded Cu_x_S, the resulting composite effectively addresses the performance instability typically observed in conventional substrate materials under high‐temperature and high‐humidity conditions [43]. To further enhance interfacial characteristics, Yu and co‐workers examined the correlation between the surface roughness of PET and the leakage current in deformable conductors. By employing a PI‐based surface modification strategy to reduce surface roughness, they significantly improved the interfacial properties of the PET substrate [169]. Similarly, Zhang's team fabricated a highly deformable conductor by spin‐coating ultrathin graphdiyne (GDY) nanosheets onto a PET substrate coated with indium tin oxide (ITO). The resulting GDY/PET composite maintained excellent stability after repeated bending cycles, demonstrating mechanical resilience of 45°, 90°, and 180° for up to 1000 cycles [44]. In the development of stretchable displays, PET is frequently employed as a substrate for rigid islands due to its excellent transparency and mechanical strength, which serve to shield active components from excessive deformation. Nam et al. demonstrated the dynamic load‐bearing capacity of PET substrates through their OLED device design, where island‐interconnect structures were fabricated via laser patterning and organic‐inorganic thin‐film encapsulation techniques. This configuration maintained stable conductive pathways under 95% strain while enduring 100 000 stretch‐release cycles [170]. For the synergistic deformation mechanism between PET substrates and functional layers, Cai et al. developed a PEDOT: PSS/Ag grid‐based flexible conductor on PET substrates using direct inkjet printing technology. This configuration preserves the inherent flexibility of PET while maintaining stable electrical performance under various bending and twisting conditions. Remarkably, the composite demonstrates excellent conductivity and mechanical stability even after 5000 bending cycles [171].

As the field progresses, while PET remains an indispensable transparent substrate, further research is required to optimize the interfacial adhesion between the chemically inert PET surface and functional conductive layers, ensuring long‐term reliability under dynamic and high‐strain conditions.

#### 2.1.5 Polyimide

Polyimide (PI) has been widely adopted as the flexible substrate in advanced deformable electronic devices due to its exceptional thermal resistance, outstanding mechanical properties, high elastic modulus, and excellent chemical stability. Recent studies demonstrate that PI can maintain both mechanical integrity and electrical performance while integrating functional thin‐film layers, making it highly competitive for various wearable and flexible device applications.

PI substrates exhibit a unique combination of high tensile strength, exceptional thermal resistance, and chemical stability, making them a cornerstone for flexible electronics. By constructing highly flexible bilayer architectures through graphene ink assembly on PI substrates, the composite achieves significantly enhanced conductivity while maintaining superior flexibility. Advanced processing techniques like vacuum plasma systems can effectively strengthen the interfacial adhesion between graphene and PI, ensuring exceptional durability under bending conditions. The interfacial energy between graphene and PI improved from 0.72 J/m^2^ (peeling separation) and 4.02 J/m^2^ (shear separation) to 2.54 J/m^2^ and 14.02 J/m^2^ after plasma treatment, respectively [172]. PI has also been utilized as a flexible substrate for bioinspired sensor designs. For example, Wang et al. fabricated parallel‐aligned PI substrates coated with silver nanoparticles (AgNPs) via electrospinning technology, mimicking the sensory whisker structure of star‐nosed moles Eimer's organ. The mole‐inspired sensor, encapsulated in PDMS, exhibited high sensitivity with robust durability over 5000 cycles, enabling effective detection of anisotropic motion and pressure (Figure 6a) [42]. This application of PI highlights its multifunctionality in combining mechanical flexibility with exceptional thermal insulation, which is critical for high‐precision aerodynamic sensing. PI substrates can maintain excellent mechanical performance under high‐temperature conditions while offering good flexibility, allowing them to withstand significant bending and twisting without fracture. Gong et al. designed the PI substrate in a thin‐film configuration, which not only reduces the overall weight of the deformable conductor but also enhances its flexibility. The low thermal conductivity of the PI film effectively minimizes heat loss from the suspended thermistor array to the substrate, enabling an unprecedented velocity resolution of 0.11 mm/s and an angular resolution of 0.1°. Such thin PI films can better conform to curved surfaces, making them suitable for integration onto complex aerodynamic structures, such as the leading edge of aircraft wings (Figure 6b) [173].

**FIGURE 6:**
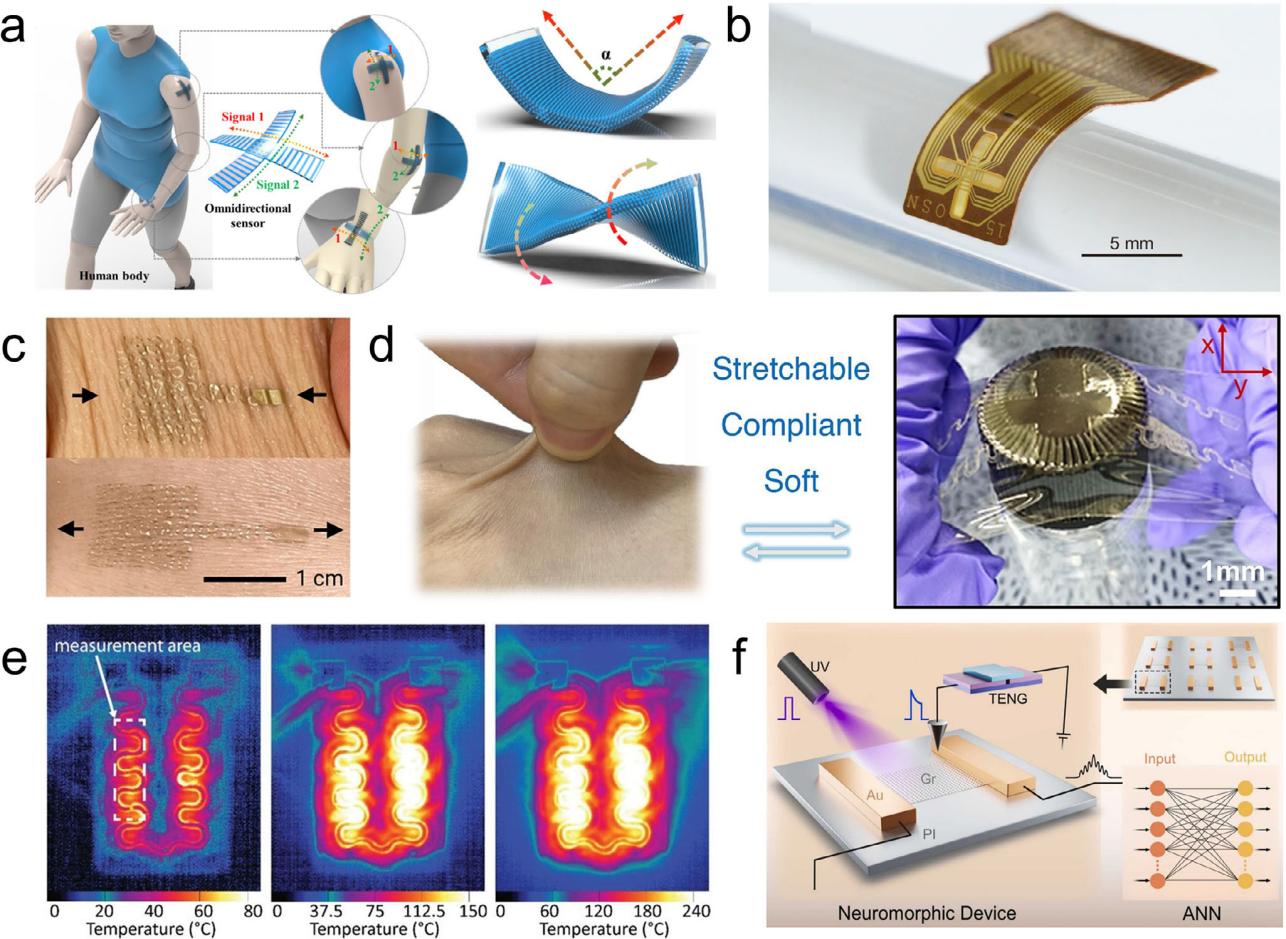
Functionalized PI substrates. (a) Schematic diagram of a flexible substrate using PI. Reproduced with permission [42]. Copyright 2021, Elsevier. (b) Schematic diagram of sufficient flexibility to adhere to curved surfaces. Reproduced with permission [173]. Copyright 2024, Springer Nature. (c) The substrate offers highly conformal lamination on the human skin while maintaining contact quality under extension. Reproduced with permission [174]. Copyright 2023, American Chemical Society. (d) Schematic diagram of the substrate stretched in *x*–*y* direction showing skin‐like resilience and stretchability. Reproduced with permission [175]. Copyright 2024, Elsevier. (e) Schematic diagram of a high‐temperature‐resistant substrate. Reproduced with permission [177] Copyright 2023, Wiley‐Blackwell. (f) The neuromorphic optoelectronic synapse device and working process for handwritten digit recognition. Reproduced with permission [181]. Copyright 2023, Elsevier.

In the field of manufacturing, screen‐printing technology based on PI composites has been widely employed to develop scalable and cost‐effective wearable electronic devices. Park et al. enhanced the printability and mechanical adaptability of the material by blending PI with PEG. This modification enables the fabrication of real‐time health monitoring systems such as electrocardiogram (ECG) devices. Compared to multi‐step cleanroom microfabrication processes, this approach offers notable advantages in terms of simplicity, low cost, and scalability for PI‐based substrates (Figure 6c) [174]. Beyond manufacturing efficiency, addressing the mechanical mismatch between rigid components and soft substrates is equally vital. Wang et al. developed an innovative strain‐engineered substrate (SES) based on poly(imide‐co‐siloxane) (PIS) copolymers to address the integration challenges between rigid electronic components and deformable systems. By incorporating rigid islands into flexible PI‐siloxane copolymers, the substrates achieved an effective balance between mechanical support and flexibility. Specifically, the substrate effectively concentrates mechanical strain in the elastic regions while shielding the active components from excessive deformation. This design effectively mitigated interfacial delamination, further highlighting the versatility of PI in flexible electronic applications (Figure 6d) [175]. Expanding the functional boundaries further, Xiong and collaborators successfully realized the in situ growth of indium antimonide (InSb) nanosheets on both silicon and PI substrates, demonstrating the feasibility of integrating PI‐based substrates with semiconductor materials for high‐performance electronic devices [176].

From a microscopic perspective, the high‐temperature resistance of polyimide (PI) is primarily attributed to the rigidity and thermal stability of its molecular chains. The imide ring structure within the PI backbone exhibits strong chemical bond stability at elevated temperatures, effectively resisting thermal degradation. Additionally, during the curing process, PI releases water molecules, leading to the formation of a highly cross‐linked network structure. This network further enhances the thermal stability and mechanical strength of the material, to maintain their functional integrity at operating temperatures exceeding 300°C (Figure 6e) [177]. Compared to other polymers such as PDMS, PVDF, and PMMA, PI exhibits superior resistance to high temperatures, making it well suited for use in extreme thermal environments. For example, in oil exploration applications where operating temperatures typically range from 150°C to 200°C, Sun et al. developed a flexible piezoelectric energy harvester (PEH) and sensor based on a PI/BLF‐PT 0–3 composite. Experimental results demonstrated that the PI‐based PEH, incorporating (Bi, La) FeO_3_PbTiO_3_ (‐PT) ceramic powder, remained functional at temperatures up to 300°C and was capable of generating an output voltage of 30 V. In contrast, PEHs fabricated using PVDF and PDMS showed negligible electrical output under the same thermal conditions. Additionally, the PI‐based flexible energy harvester maintained a stable voltage output after 12 h of continuous operation while attached to a freely moving finger, illustrating its excellent flexibility and mechanical durability [178]. As reported by Liu et al., PI exhibits superior high‐temperature resistance and structural stability compared to other polymers due to its high glass transition temperature. This makes it an ideal flexible substrate for high‐temperature applications. The resulting flexible pressure sensors demonstrate excellent compressibility, high sensitivity (158.23 kPa^−^^1^ at room temperature, decreasing to 71.47 kPa^−^^1^ at 150°C, and 7.63 kPa^−^^1^ at 250°C), and remarkable durability (5000 cycles at room temperature and 2500 cycles at 150°C [179].

In addition, photosensitive PI incorporating trifluoromethyl moieties and spiropyran derivatives enables sustainable and reversible metal patterning. This innovative approach utilizes ultraviolet light to induce selective metallization on PI surfaces, forming high‐resolution and highly conductive copper (Cu) patterns without conventional complex processing steps. This advancement underscores the potential of PI as a multifunctional flexible substrate, capable of supporting high‐resolution circuit fabrication while retaining chemical and thermal stability [180]. Guo and colleagues developed a PI/graphene heterojunction by transferring chemical vapor deposition (CVD) grown monolayer graphene from a copper foil onto a flexible PI substrate. This structure leveraged the UV absorption characteristics of PI and the high conductivity of graphene to achieve photoelectric signal conversion. The PI substrate not only provides mechanical flexibility for the device but also actively participates in the optoelectronic conversion process, particularly in applications simulating human visual functions (Figure 6f) [181].

Despite its numerous advantages as a deformable substrate, including high thermal resistance and mechanical strength, PI still faces challenges in enhancing interfacial adhesion with other functional layers and achieving seamless integration under high‐strain conditions. Further research may focus on optimizing PI surface modification strategies. For instance, acid‐base treatments can be employed to increase surface energy, and improve wettability, thereby promoting stronger adhesive bonding to the film surface. Plasma treatments are also promising for enhancing surface roughness and hydrophilicity. Additionally, surface grafting techniques that introduce various functional groups or nanomaterials onto the PI surface can further improve its interfacial properties. For example, the incorporation of hydrophilic groups can elevate surface energy, enhance wettability and adhesion, and broaden the applicability of PI in optical coatings and flexible electronic devices [182, 183].

#### 2.1.6 Other Substrates

In addition to the commonly used substrate or matrix materials for deformable conductors mentioned above, numerous studies have explored a variety of alternative materials to broaden the application scope of deformable conductor materials. By addressing material properties, structural design strategies, and specific applications, these studies demonstrate the breadth and innovation of current research in flexible electronics.

For instance, Zhang et al. developed a fish gelatin (FG) film [72] as a biodegradable substrate material, demonstrating excellent biocompatibility and optical transparency. When integrated with AgNWs, the FG film was employed as a substrate for alternating current electroluminescent (ACEL) devices. The resulting device exhibited not only high flexibility and strong luminescent performance but also rapid water‐induced degradation. These characteristics highlight its strong potential for use in wearable electronics and biomedical devices, particularly in applications where sustainability and environmental compatibility are essential [53]. Similarly, poly(octamethylene maleate (anhydride) citrate) (POMaC), a citrate‐based polyester, represents another biodegradable elastic material, whose molecular structure can be precisely engineered through tunable chemical design to achieve an optimal balance between mechanical strength and degradation rate, which is suitable not only for flexible electronic substrates but also for meeting biodegradable requirements in implantable medical devices. However, its current fabrication process remains relatively complex and costly, necessitating further optimization before it can be applied at scale [184].

Ecoflex [185, 186, 187], a commercially available elastomer, is widely utilized for deformable conductors due to its low Young's modulus and high elongation capability. For example, Xu et al. embedded conductive fillers such as graphitized carbon black and sodium chloride into Ecoflex, resulting in a self‐compensating electrical system capable of maintaining stable resistance across a broad strain range. This design benefits from the strong interfacial integration between the fillers and the matrix, which ensures excellent electrical and mechanical stability during stretching and repeated deformation cycles [188].

Thermoplastic elastomers (TPE) [189, 190, 191] have also been employed as deformable substrates owing to their high elasticity and excellent stretchability. Zhou et al. utilized TPE to fabricate coaxial fiber structures in which a self‐curling conductive core was embedded within a TPE channel to form stretchable fibers. This unique coaxial architecture effectively decouples mechanical deformation from electrical path disruption, allowing the fibers to withstand an ultra‐high fracture strain of 659.2%. Notably, these conductors maintain remarkable electrical stability, with resistance variations kept within a minimal range (approximately 4% under moderate cyclic strains). Additionally, these fibers can be produced through wet spinning, allowing for scalable and continuous manufacturing, which significantly enhances their potential for commercial applications [192].

The substrate and matrix materials discussed in these studies fundamentally dictate the functional boundaries and operational stability of deformable conductors. As the field progresses, future research must pivot from generic material exploration toward highly targeted structural and chemical designs. Specifically, at the molecular level [94, 95], incorporating dynamic reversible crosslinks [97, 98] into polymer networks will be critical for realizing intrinsic self‐healing, recyclability, and adaptable moduli. At the interfacial level, targeted surface functionalization design is urgently needed to mitigate the mechanical mismatch and prevent the delamination of rigid or fluidic conductive fillers under extreme multidirectional strain. In parallel, the development of cost‐effective and scalable fabrication techniques will be essential to facilitate the practical application and commercialization of these advanced deformable substrate materials.

### 2.2 Conductive Materials

Conductive materials play a fundamental role in the development of deformable conductors. These materials must exhibit excellent electrical conductivity as well as possess mechanical flexibility to accommodate complex deformations. The selection and design of conductive materials directly influence the electrical, mechanical, and operational performance of deformable conductors. It is worth noting that these conductive materials can be integrated into deformable systems in various structural forms. Rather than being restricted solely to conductive fillers dispersed within a polymer matrix, they are also extensively utilized as surface coatings, printed patterns, or continuous thin films on the surface of elastomeric substrates. Commonly used conductive materials include carbon‐based, metal‐based materials, conductive polymers, and composites, each offering distinct advantages depending on the applications. Carbon‐based materials, such as graphene, CNTs, and carbon nanofibers (CNFs), are widely employed due to their robust structural stability, which ensures sustained electrical performance under long‐term cyclic loading. Importantly, from a manufacturing perspective, carbon materials offer a substantial economic advantage over metal nanomaterials. The raw precursors for carbon structures are highly abundant and intrinsically inexpensive. While synthesizing perfectly crystalline graphene or pristine CNTs traditionally requires energy‐intensive CVD [193], the maturation of industrial‐scale mass production for commercial CNTs, reduced graphene oxide (rGO), and carbon black has drastically driven down their market prices. Metal‐based conductive materials, encompassing metallic nanowires, nanoparticles, and liquid metals, are distinguished by their exceptional electrical conductivity. However, the fabrication of these metallic nanomaterials typically incurs higher costs compared to carbon‐based alternatives. The production of widely utilized noble metal nanomaterials is inherently constrained by the elevated market prices of their metallic precursors. Furthermore, conventional bottom‐up wet chemical protocols necessitate expensive structure‐directing agents or substantial volumes of organic solvents [194], compounded by protracted, low‐yield purification processes. These factors collectively render their synthesis significantly more capital intensive than that of carbonaceous materials. While base metals such as copper or aluminum offer low bulk costs, the synthesis of high‐aspect‐ratio, oxidation‐resistant copper nanowires often mandates stringent inert atmospheres or intricate core–shell protective architectures. Such requirements inadvertently escalate the overall manufacturing expenditure. Conductive polymers such as PANI and polypyrrole (PPy) offer a unique combination of flexibility, processability, and tunable electrical properties through chemical doping. These polymers can maintain continuous conductive networks during mechanical deformation and are less susceptible to fracture or network disruption. Conductive polymers exhibit better adaptability for integration into flexible electronic devices. Their relatively low material cost and compatibility with solution‐based fabrication processes make them promising candidates for scalable manufacturing. However, a trade‐off often exists between electrical conductivity and mechanical performance, necessitating ongoing research into performance optimization. Composite conductive materials integrate the benefits of carbon‐based materials, metals, and conductive polymers. Through synergistic interactions between components, composites can simultaneously achieve electrical conductivity, stretchability, and mechanical durability. Nonetheless, the cost of composite systems can be relatively high, depending on material selection and synthesis complexity. Furthermore, their fabrication frequently involves multi‐step processing, structural design optimization, and precise control of the proportion of materials, which collectively present substantial engineering and manufacturing challenges.

#### 2.2.1 Carbon‐Based Materials

The development of flexible electronic devices and deformable conductors is strongly supported by advances in conductive materials. Among these, carbon‐based materials [50, 195, 196] have attracted considerable attention due to their mechanical stability and high design flexibility within flexible composite systems. The electrical conductivity of these materials is closely related to their intrinsic structure and morphology. Graphene [39], CNFs [30], and CNTs [51, 197, 198] are notable examples, all exhibiting excellent electrical conductivity, which renders them essential components in the construction of high‐performance deformable conductors. When integrated with various substrate materials and functional fillers, carbon‐based materials contribute to the formation of conductive networks that combine flexibility, mechanical robustness, and electrical functionality. Specifically, the electrical conduction within these carbon‐based composites is primarily governed by two distinct mechanisms: Ohmic conduction (percolation), where electrons travel through direct physical contacts between adjacent carbon fillers, and quantum tunneling (or hopping), where electrons traverse thin insulating polymer barriers between closely spaced, non‐contacting fillers [199]. Notably, under large mechanical strains, these materials can maintain continuous conductive pathways by dynamically shifting their reliance toward the tunneling mechanism, effectively adapting to complex deformation modes, making them ideal candidates for use in deformable conductor systems. The section discusses the applications of representative carbon‐based materials in deformable conductors, focusing on their performance advantages under mechanical deformation.

Graphene, as the most representative carbon‐based conductive material, has become a pivotal component in deformable conductors due to its two‐dimensional planar structure and exceptional electrical conductivity. These properties have led to its extensive application in high‐sensitivity stretchable sensors and flexible electronic devices. For example, Wang et al. developed a deformable conductor based on a wavy graphene/TPU composite. This composite exhibited only a 22% increase in resistance under 150% strain, attributed to its hierarchical porous structure and wavy surface morphology, while maintaining a high electrical conductivity of 7.2 S/cm [47]. These results highlight the outstanding ability of graphene to maintain stable conductive pathways under large mechanical deformations. Wang et al. fabricated laser‐induced graphene (LIG), further illustrating the adaptability of graphene in flexible electronic systems. By integrating LIG with hydrogels to construct a mechanically interlocked composite with embedded conductive pathways, the resulting device achieved less than 4% resistance variation under 200% tensile strain [26]. This approach ensures stable electrical responses under cyclic strain conditions while also enabling effective thermal and humidity management, thereby making it particularly suitable for wearable stretchable electronic applications. Chen et al. fabricated a graphene foam/PDMS composite using the CVD method, forming a high‐quality, 3D continuous foam network with an electrical conductivity of approximately 10 S/cm. The resulting deformable conductor exhibited only a 30% resistance variation under 50% strain, which was attributed to the structural adaptability of the porous graphene channels during deformation [200]. Concurrently, advancements in graphene ink printing technology have expanded its application potential. For example, Van Hazendonk et al. printed graphene nanosheet ink onto TPU, producing a deformable conductor that maintained less than 6% resistance variation after 1000 strain cycles at 20%–50% strain [48]. This fabrication approach demonstrates significant potential for scalable manufacturing and customized design of graphene‐based deformable conductors. However, maintaining an optimal balance between electrical conductivity and mechanical flexibility, particularly under high filler loadings, remains an important area for future research and optimization.

CNFs have been widely employed as conductive materials for deformable conductors due to their high conductivity, durability, and flexibility. Typically embedded in various polymer substrates, CNFs leverage their one‐dimensional (1D) structure and exceptionally high aspect ratio (with diameters ranging from several nanometers to tens of nanometers and lengths reaching micrometer or even millimeter scales) to establish efficient conductive pathways while providing mechanical reinforcement. When combined with elastomeric polymers such as PDMS, PVA, or specialized biodegradable substrates, the resulting deformable conductors maintain stable electrical performance under significant mechanical deformation, including stretching, bending, and compression [201]. The graphitic structure of CNFs, achieved through high‐temperature thermal treatment that rearranges carbon atoms into graphite‐like microstructures, exhibits enhanced mechanical, electrical, and thermal properties. When uniformly dispersed in polymer matrices, graphitized CNFs form interconnected conductive networks that facilitate efficient charge transport. However, a critical challenge in CNF‐based applications lies in their inherent mechanical weakness, which prevents their direct use as standalone conductive materials. To address this, researchers have focused on optimizing electrospinning processes to produce CNFs with tunable structures that balance conductivity and mechanical flexibility. This approach has emerged as a key strategy for performance enhancement, enabling the fabrication of CNFs that meet the stringent requirements of deformable electronics. For example, Wei et al. prepared deformable conductors with CNFs as the conductive component by electrospinning polyacrylonitrile (PAN) nanofiber membranes, followed by stabilization and carbonization treatments. The carbonized CNFs exhibited high specific surface area, aligned graphitic layers, and adaptable structures. Four‐point probe measurements showed that CNFs prepared at a carbonization temperature of 1000°C achieved the highest conductivity of 6.02 S/cm [202]. CNFs are often combined with other materials to obtain desired properties. For instance, Vahdani et al. doped CNFs with gold (Au) to improve the conductivity of the conductor and its sensitivity in piezoresistive sensors. This combination enhanced the strain‐sensing capability of the material while maintaining its tensile properties [203]. Stretchable conductive circuits based on CNFs are not only used in strain sensors but also in capacitive touch devices, energy storage components, and biomedical devices. Their multifunctionality makes them attractive for applications ranging from human–machine interfaces to implantable electronics. However, achieving uniform dispersion of CNFs is often challenging due to their tendency to agglomerate. Additionally, the high production cost of purified CNFs and the complexity of composite processing may limit their large‐scale industrial applications [204].

As a representative carbon‐based material, CNTs are among the most extensively utilized components in the development of deformable conductors. Their high aspect ratio and exceptional axial electrical conductivity enable significant reductions in material usage while minimally affecting the mechanical properties of compound matrices. In addition, CNTs exhibit outstanding mechanical strength, allowing them to maintain stable electrical performance even under cyclic stretching and repeated mechanical deformation [205, 206]. Sun et al. incorporated CNTs into a PDMS matrix to fabricate a deformable conductor, which was subsequently employed in a flexible tactile sensor capable of detecting 3D forces. The dual‐sided porous structure of the CNT networks conferred high sensitivity to the sensor, achieving a response rate of 12.1 kPa^−^^1^ within a low‐pressure range of 600 Pa. This high‐pressure sensitivity reflects the superior electromechanical performance of the CNT‐based deformable conductor. Within this system, CNTs significantly enhanced the piezoresistive response by forming stable and continuous conductive networks, ensuring reliable signal transmission under varying mechanical loads [147]. The integration of CNTs with other conductive nanomaterials in layered nanocomposite designs has shown great promise in achieving high stretchability and conductivity. For example, Lee et al. utilized CNTs to establish localized percolation networks that operated synergistically with a high‐conductivity AgNW framework. This multiscale hybrid design capitalized on the inherent flexibility and stretchability of CNTs while simultaneously enhancing charge transport efficiency. The resulting deformable conductor exhibited robust performance, maintaining electrical conductivity under strains exceeding 460%, following over 10 000 bending cycles, and during large‐angle twisting and complete folding deformations [55]. CNTs exhibit high mechanical and chemical stability as well as excellent electrical conductivity. In addition, they can be processed via solution‐based methods, facilitating their integration with other materials. Min et al. employed a spray‐coating technique to deposit multi‐walled carbon nanotubes (MWCNTs) onto pre‐stretched PDMS substrates that had been plasma‐treated. Upon releasing the pre‐strain, the resulting wrinkled structure of the PDMS enabled the formation of a stable conductive network of MWCNTs on its surface (Figure 7a–c) [207]. Despite these advantages, CNT‐based conductors still face challenges, including uniform dispersion in the substrate, aggregation, and relatively high production costs [208]. One promising strategy to address these limitations involves the use of surfactants or chemically functionalized CNTs to improve dispersion and interfacial adhesion within the substrate. Additionally, hybrid composite designs that integrate CNTs with other conductive nanomaterials offer a viable path to enhancing both electrical conductivity and mechanical durability, thereby broadening the applicability of CNT‐based deformable conductors.

**FIGURE 7:**
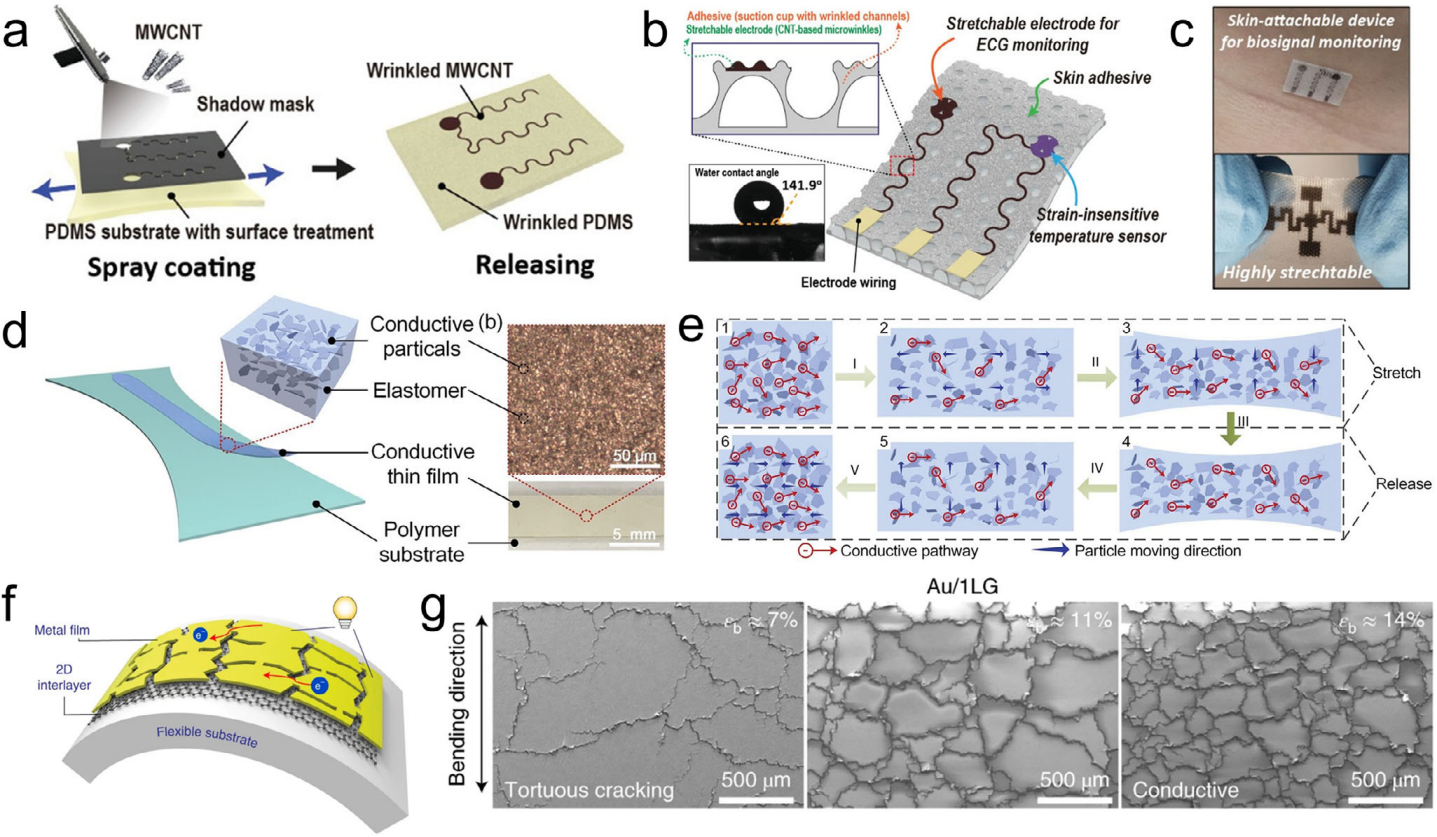
Carbon materials, metal particles and metal films as conductive materials. (a) Schematic of the fabrication of the isotropic microwrinkle structure. Reproduced with permission [207]. Copyright 2022, Wiley‐VCH. (b) The inset shows a side view of the APSE structures and the water CA of the wrinkled APSE. Reproduced with permission [207]. Copyright 2022, Wiley‐VCH. (c) Photograph showing the APSE's conformal adhesion to wet human skin and high stretchability. Reproduced with permission [207]. Copyright 2022, Wiley‐VCH. (d) Schematic illustration of a conductive elastomer containing a conductive thin film printed on a stretchable polymer substrate. Reproduced with permission [209]. Copyright 2022, Wiley‐VCH. (e) Movements of conductive particles and changes of conductive pathways in the conductive elastomer during a stretch‐release cycle. Reproduced with permission [209]. Copyright 2022, Wiley‐VCH. (f) Schematic of a flexible metal electrode with an interlayer of atomically thin 2D material. Reproduced with permission [210]. Copyright 2021, Springer Nature. (g) Crack progression at various bending strains (*ε*_b_) in a bare Au electrode. Reproduced with permission [210]. Copyright 2021, Springer Nature.

In summary, carbon‐based materials demonstrate significant potential for applications in deformable conductors. Materials such as graphene, CNTs, and CNFs offer not only electrical conductivity but also superior mechanical strength and flexibility, providing robust support for the advancement of next‐generation flexible electronics. Nonetheless, several challenges persist, including issues related to uniform dispersion, interfacial bonding with polymer matrices, complicate fabrication processes, and high production costs. Through continuous optimization of material architectures and composite fabrication strategies, carbon‐based conductive systems are expected to play an increasingly vital role in complex and multifunctional applications, particularly in wearable electronics, health monitoring technologies, and artificial intelligence‐integrated sensing platforms.

#### 2.2.2 Metallic Materials

Metallic materials are extensively utilized in the fabrication of deformable conductors owing to their exceptional electrical conductivity and versatile structural configurations. These materials are available in a variety of forms, including metal nanowires [3, 70, 211, 212, 213, 214, 215], metal nanoparticles [82, 216], metal thin films [217, 218, 219, 220], and liquid metals [221, 222, 223, 224, 225]. Each form offers distinct advantages, thereby providing a broad spectrum of design options for deformable conductors. From the high flexibility of metallic nanowires to the fluidity and adaptability of liquid metals, the distinct properties of each metallic material provide vast possibilities for developing deformable conductors. In particular, metallic nanowires, such as those made of Ag, Au, and Cu, enable widespread applications of deformable conductors in various fields due to their high conductivity, excellent transparency, and superior biocompatibility. The incorporation of metallic nanoparticles and thin films significantly improves the mechanical and electrical reliability of deformable conductors. This is achieved not only through microcrack mitigation and surface self‐healing mechanisms, but also through enhanced resistance to oxidation, which prevents degradation of conductive pathways and ensures long‐term stability in ambient or reactive environments. Additionally, liquid metals have rapidly emerged due to their fluidity and high electrical conductivity.

Owing to their unique combination of high conductivity, flexibility, and biocompatibility, metal nanowires (MNWs) have emerged as a cornerstone material in the development of deformable conductors. Their low percolation threshold also makes them suitable for the design of transparent deformable conductors [226, 227]. For example, Kim and colleagues fabricated a deformable conductor by embedding AgNWs into a TPU substrate, creating an electrode material with light‐induced self‐healing capabilities. When exposed to intense pulsed light (IPL), localized heating of the AgNWs repaired cracks caused by mechanical stretching or external damage [161]. To address the issue of nanowire network fractures under high strain, Kim et al. proposed a design strategy that leveraged the buckling instability of 1D nanowire networks. By depositing AgNWs onto a pre‐stretched elastic substrate and allowing the formation of a wavy structure upon relaxation, the researchers mitigated strain‐induced damage while preserving the transparency and conductivity of the conductor. This structural engineering approach significantly improved the overall stretchability of the deformable conductor without compromising its optical or electrical performance [228]. As demonstrated previously, engineering AgNWs into wavy or mesh‐like configurations helps preserve the integrity of the conductive networks under high tensile strain. Xu et al. embedded AgNWs into the surface layer of a PDMS substrate, achieving an electrical conductivity of approximately 8130 S/cm. Even under 50% strain, the electrical resistance exhibited only minor variation [31]. This performance is primarily attributed to the sliding and reorganization of the nanowire network during deformation, which effectively dissipates mechanical stress while maintaining the conductivity of the conductive pathway. AgNW‐based transparent conductive films have also been extensively utilized in flexible display technologies. Ding et al. summarized several optimization strategies involving AgNW spatial distribution, grid architecture, and surface treatments to improve conductor stability under high‐strain conditions [212]. In a related advancement, Seufert and co‐researchers developed a scalable and stable synthesis technique using polymeric reducing agents to produce size‐controlled gold nanowires (AuNWs) with excellent biocompatibility and high aspect ratios. The resulting deformable conductor demonstrated neuron‐like softness (approximately 250 kPa), high conductivity (16 000 S/cm), and reversible stretchability, making it a promising condition for bio‐integrated electronic applications [52]. In addition to AuNWs, copper nanowires (CuNWs) have also attracted extensive research interest due to their unique 1D morphology, high aspect ratio, low annealing temperature, and efficient electron transport properties. Compared to other metal nanowires, CuNWs offer a significantly more economically viable alternative [229]. For instance, Kim et al. developed highly conductive, transparent, and flexible CuNW electrodes by synthesizing CuNWs of various sizes through hydrothermally growth and embedding them in UV‐curable polyurethane acrylate (PUA) resin as conductive pathways. The resulting conductor demonstrated excellent mechanical stability and electrical conductivity under compressive and bending stresses [45]. Furthermore, coating the nanowire surfaces with polymer or oxide protective layers can substantially enhance their durability and oxidation resistance, thereby extending the service life of deformable conductors [230, 231, 232].

Metallic nanoparticles are also commonly employed as conductive materials in deformable conductors. When embedded into elastomeric substrates, nanoparticles such as Au, Cu, and Ag can form uniform conductive networks, enabling an effective balance between mechanical flexibility and electrical conductivity. For instance, Kim et al. utilized layer‐by‐layer (LBL) deposition to integrate spherical Au nanoparticles with polyurethane, creating a deformable conductor with high conductivity (11 000 S/cm at 21.7 vol% Au) and stretchability. Although spherical nanoparticles are typically less conductive than high‐aspect‐ratio nanowires, they exhibited dynamic self‐organization under strain, rearranging into chain‐like conductive pathways to maintain electrical connectivity [58]. In another approach, Lyu et al. developed a high‐strength, highly conductive deformable conductor by combining Au nanoparticles with aramid nanofiber (ANF). The exceptional mechanical strength and thermal stability of ANFs provided a robust framework for nanoparticle alignment, while subsequent thermal treatment further enhanced conductivity to 12 500 S/cm [233]. Cu has garnered significant attention for its exceptional cost‐effectiveness and superior conductivity compared to precious metals. However, the practical application of Cu nanomaterials faces limitations due to their susceptibility to oxidation under ambient conditions. To overcome this limitation, Kwon et al. employed a bar‐coating technique to deposit Cu nanoparticles onto a polyethylene naphthalate (PEN) substrate, forming conductive pathways. Subsequent localized laser sintering in an inert gas environment not only enhanced conductivity but also prevented oxidation, resulting in stable stretchable conductive pathways. The fabricated deformable conductor demonstrated remarkable durability, with only ±10% resistance variation after 1000 bending cycles [234]. AgNPs represent another prevalent conductive material for deformable conductors. Park et al. demonstrated this approach by integrating Ag nanoparticles with elastic fibers, using electrospun poly(styrene‐butadiene‐styrene) (SBS) fibers as substrates to create highly stretchable circuits. The Ag nanoparticles not only formed continuous conductive networks within the fibers but also generated conductive Ag shells on the fiber surface, achieving a conductivity of approximately 2200 S/cm at 100% strain for 150 µm‐thick samples [235]. Alternatively, Tan improved conductive composites by embedding nickel (Ni) particles in a nylon‐6 matrix. At 30 vol% Ni loading, the composite reached a conductivity of approximately 310 S/cm [59]. In deformable conductors, metal particles are uniformly dispersed within the substrate, forming an interconnected conductive network. The contact points or quantum tunneling junctions between these particles serve as critical components of the conductive pathway. When the conductor is stretched or released, the metal particles shift in response to the deformation of the polymer substrate, thereby altering the structure of the conductive network and resulting in changes in electrical resistance (Figure 7d,e) [209]. However, long‐term reliability remains a critical challenge as nanoparticle migration and aggregation may disrupt conductive networks. This necessitates mitigation strategies such as surface modification or composite engineering to enhance stability.

Metallic thin films play a vital role in deformable conductors by providing continuous, high‐conductivity pathways that remain stable under mechanical deformation, making them suitable for various flexible electronic applications. However, most metallic films exhibit a physical fracture limit below approximately 2% tensile strain [14]. To overcome this limitation, strategies such as introducing microcracks, nanostructures, or composite materials are typically employed to enhance their performance. For example, Shu et al. developed a strain sensor composed of a PU substrate and an Au thin film featuring micro/nano‐protrusions and micro/nano‐cracks. This design achieved both a wide measurement range and high sensitivity. Experimental results demonstrated that the Au‐based flexible sensor could withstand up to 80%, significantly surpassing the 5% limit of conventional metal foil sensors. Moreover, the sensor maintained electrical stability after 1000 stretching cycles. This remarkable durability originated from the micro/nano‐protrusions on the film surface, which inhibit crack propagation by promoting the formation of numerous short cracks instead of long, continuous ones that would disrupt conductive pathway [40]. Inspired by the dermal structure of pufferfish, Sun et al. implemented an interlayer modulation strategy to achieve ultra‐stretchability (up to 295% strain) and high sensitivity (maximum gauge factor of approximately 5500) in metal thin films used for electronic skin applications. An intermediate FeO_x_ layer with tunable surface morphology was introduced between the elastic substrate and the metal thin film conductor. This interlayer effectively regulated crack propagation during mechanical deformation, thereby dissipating strain energy and preserving the continuity of the conductive pathways [236]. Han and colleagues designed an innovative transparent flexible electrode featuring a bilayer stacked metal film structure. This electrode exhibited high optical transmittance of approximately 90%, low sheet resistance below 3 Ω/sq, and mechanical flexibility. It withstood 10 000 bending cycles at a curvature radius of 1 mm without exhibiting significant changes in resistance. Moreover, this design also offered facile patterning capabilities [237]. In another study, Wang et al. fabricated a strain sensor by depositing a gold/titanium (Au/Ti) bilayer onto PDMS substrate, where the Ti layer functioned as the adhesion layer and the Au layer served as the conductive layer. Microcracks were introduced through pre‐stretching, and these cracks remained closed upon strain release. The sensing mechanism was governed by the microcrack overlap modes and electron tunneling effects. Notably, this metal thin film sensor was capable of detecting minor strains in non‐joint areas of the human body [238]. Trost et al. fabricated molybdenum‐silver alloy films on flexible PI substrates using co‐sputtering technology. Due to the high mixing enthalpy of Molybdenum (Mo) and Ag, the alloy exists in a metastable state. This unique property enables Ag atoms to migrate and fill cracks during tensile deformation, thereby restoring electrical conductivity [239]. Au thin‐film electrodes are prone to fracture even under small strains, leading to rapid surface crack propagation and eventual interfacial failure, which causes a sudden loss of electrical performance. This brittle fracture behavior severely limits the functional lifespan of flexible devices under various deformations. Cho et al. introduced a two‐dimensional material interlayer between the Au film and the flexible substrate, which significantly altered the crack propagation behavior. The cracks followed a more tortuous path, forming polygonal interconnected regions, thereby successfully enhancing the strain‐tolerant electrical performance of the Au electrodes (Figure 7f,g) [210]. Lee and colleagues developed highly conductive aluminum (Al) films through an innovative solution stamping process suitable for flexible substrates. Compared to solution‐processed Ag and Au films, these Al films demonstrated superior performance when fabricated at temperatures of 150°C or lower, achieving a remarkably low specific electrical resistance (below 16 µΩ·cm) and high conductivity (6.25 × 10^3^ S/cm) [240].

Different from traditional solid‐state metal materials, liquid metals are utilized as deformable conductors owing to their fluidity and conductivity, and are generally embedded within elastomer substrates such as PDMS [241], polyurethane [60], and Ecoflex to serve as conductive pathways. These materials can adaptively deform within the substrate while maintaining stable electrical performance. At or near room temperature, liquid metals represent the only materials that simultaneously exhibit metallic properties and fluid characteristics. The known liquid‐phase metallic elements include cesium (Cs), francium (Fr), rubidium (Rb), mercury (Hg), and Ga. However, despite its low melting point, Cs finds limited applications due to its highly reactive chemical nature. Fr, as a radioactive alkali metal, has severely restricted practical utility, and Rb is similarly unsuitable for applications owing to its radioactivity and high reactivity. Hg presents toxicity concerns and possesses an extremely high surface tension (487 mN/m), which complicates processing and material compatibility [242]. In comparison, Ga shares numerous physical properties with Hg but demonstrates significantly reduced toxicity. Moreover, Ga and its alloys exhibit high electrical conductivity and favorable biocompatibility. Eutectic gallium‐indium (EGaIn), a representative gallium‐based liquid metal alloy composed of Ga and indium (In), offers several desirable properties including a low melting point, high electrical conductivity, low vapor pressure, and low viscosity. Park et al. employed EGaIn as a conductive material that could be uniformly spread across chemically cross‐linked hydrogel surfaces, thereby enabling direct micropatterned printing on hydrogels. The resulting EGaIn‐based deformable conductor retained stable conductivity under strains as high as 1500% (Figure 8a) [241]. Similarly, Zhuang et al. employed eutectic gallium‐indium alloy (eGaIn)‐based liquid metal as the conductor, which demonstrated exceptional stretchability on a fiber substrate, withstanding strains of up to 1500% without significant changes in electrical resistance (Figure 8b) [243]. Despite these advantages, the inherently high surface tension of liquid metals often presents challenges in achieving uniform coating and precise patterning. To overcome these limitations, various strategies have been developed. For example, Zhu et al. introduced a fully solution‐processed technique for directly generating liquid metal patterns on a wide range of elastomer substrates. This method enabled the fabrication of a stretchable matrix comprising liquid metal circuit patterns integrated with light‐emitting diodes (LEDs), which maintained stable illumination in both relaxed and highly stretched states. The fabricated circuits achieved an electrical conductivity of 4.15 × 10^4^ S/cm [49]. Huang et al. achieved uniform dispersion of liquid metal microdroplets and formation of stable conductive pathways on flexible substrates through mechanical sintering or surface chemical modification. In their study, dopamine‐modified polyurethane sponges served as carriers for the liquid metal, enabling the resulting composite conductor to exhibit both high mechanical strength and excellent electrical conductivity, achieving a conductivity of 478 S/cm [60]. Ma et al. developed a novel highly breathable, ultra‐elastic liquid metal fiber mat by coating or printing liquid metal onto electrospun elastic fiber mats. The material exhibited remarkable breathability, extreme elasticity (exceeding 1800% strain), and exceptionally high conductivity (up to 18,000 S/cm). Under mechanical deformation, the liquid metal reorganized into a laterally porous and longitudinally wrinkled architecture suspended between fibers. This adaptive structure allowed the material to accommodate stretching in multiple directions, thereby offering omnidirectional stretch stability. Furthermore, both in vitro and in vivo biocompatibility evaluations confirmed the compatibility of the material with biological systems, making it a promising candidate for wearable and implantable electronics [61]. Given the difficulty of achieving traditional fine patterning with liquid metal due to its high surface tension, researchers have also proposed structural design‐based solutions, such as three‐dimensional interconnect architectures. Liquid metal, owing to its unique physical and chemical properties, plays a critical role in 3D deformable conductors. In 3D interconnect architectures, Liquid metal can be patterned into arched interconnect structures, enabling cross‐over arrangements on a single plane without short‐circuiting, while also simplifying circuit design. These 3D arched interconnects exhibit excellent mechanical stability during bending and stretching, with negligible changes in electrical resistance even after 20 000 bending cycles (Figure 8c) [225].

**FIGURE 8:**
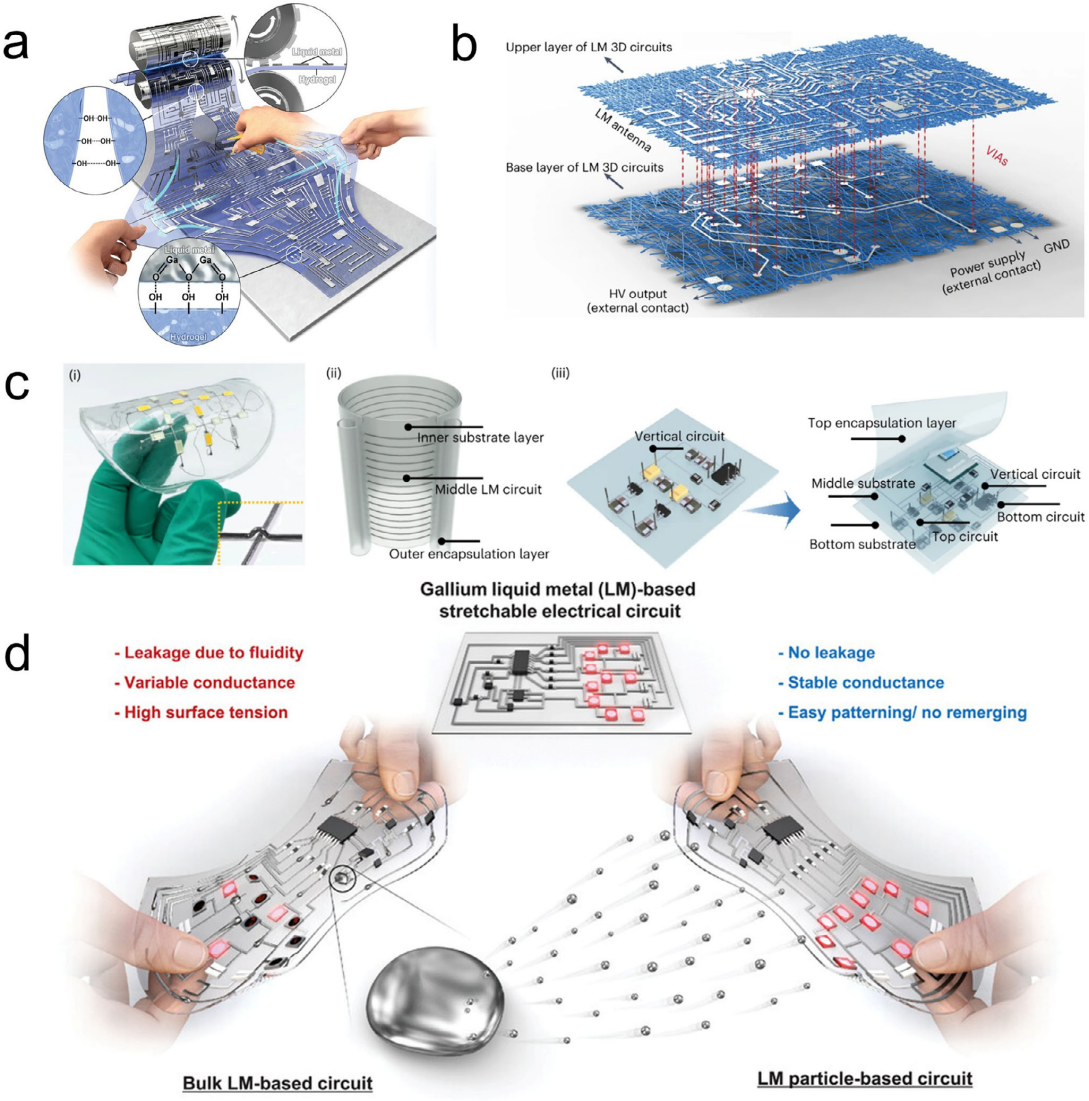
Liquid metals as conductive materials. (a) Schematic illustration of a deformable and self‐healable EGaIn electrode on a hydrogel. Reproduced with permission [241]. Copyright 2020, Wiley‐VCH. (b) LM microelectrodes are adopted as the reliable interface between the soft, rough fiber mat substrate and the rigid components. Reproduced with permission [243]. Copyright 2024, Springer Nature. (c) Flexible electronics prepared using 3D structured liquid metals solid wire: flexible LED array with arched interconnects (i), wearable 3D structured finger movement detection sensor (ii), and multilayer flexible circuit board (iii). Reproduced with permission [225]. Copyright 2023, Springer Nature. d) Schematic illustration of comparison between bulk LM and LM particles‐based electrical circuit. Reproduced with permission [252]. Copyright 2024, Wiley‐VCH.

Liquid metals, existing in a fluid state at room temperature, exhibit exceptional deformability and can readily accommodate complex mechanical deformations of deformable conductors. Unlike conventional solid conductors that tend to fracture and lose conductivity under large strains, liquid metals maintain continuous conductive pathways even during extensive stretching or bending. For instance, Tian et al. developed a sandwich‐structured deformable conductor by integrating liquid metal with a custom‐designed polyurethane (PU) elastomer. The fluidity of the liquid metal enables the formation of a uniform conductive layer on the elastomer surface, which flows along with the substrate deformation, thereby preserving electrical continuity. Notably, the device maintained stable resistance performance under up to 850% strain [244]. Similarly, Liao et al. combined liquid metal with hydrogels to fabricate an integrated deformable conductor that eliminates the need for additional encapsulation or complex interconnects, simplifying the fabrication process and reducing overall cost [245]. Beyond direct integration with substrates, liquid metal is also hybridized with other materials to enhance conductivity and functionality. Jeong et al. introduced a composite conductor composed of liquid metal and silver flakes, where pressure‐induced sintering was employed to facilitate selective interlayer connections, resulting in a reconfigurable double‐sided textile circuit. This configuration achieved a high conductivity of up to 3.14 × 10^3^ S/cm, allowing for stable current transmission under strain without significant resistance increase. The design leverages the fluidity and low surface tension of liquid metal to form controllable conductive paths across textile structures [246]. In another example, Li et al. combined liquid metal with copper materials and utilized laser‐induced selective metallization to pattern conductive circuits on polypropylene nonwoven fabric (PPNF). The resulting liquid metal‐based electronic textile maintained conductivity under mechanical deformation, while also exhibiting antibacterial and electromagnetic interference (EMI) shielding properties [247]. Additionally, liquid metal has been incorporated with lignin, where ionic and electrostatic interactions between carboxyl groups on lignin and the oxide layer on the liquid metal surface promote stable dispersion within the polymer matrix [248]. Luo et al. further advanced this field by formulating a self‐healing liquid metal ink through the combination of liquid metal with infusive polydimethylsiloxane (H‐PDMS). This approach enhanced the dispersion and interfacial adhesion of liquid metal within the substrate, enabling the formation of a stable conductive network that maintained excellent electrical performance under stretching and bending. Moreover, the self‐healing capability of the H‐PDMS matrix offered additional protection to the conductive pathway, allowing the conductor to retain high performance after repeated damage and healing cycles [249].

The surface properties of liquid metals significantly impact their practical application. When exposed to air, liquid metals such as gallium‐based alloys rapidly form a robust oxide film (gallium oxide) on their surface. Although the oxide layer enhances mechanical stability and effectively suppresses irregular flow on substrate surfaces to enable precise patterning on elastic substrates, it introduces elevated electrical resistance, which can hinder overall conductivity. To address this issue, researchers have developed various electrochemical and mechanical approaches to remove the oxide layer and restore conductivity. For example, Ford et al. demonstrated that mechanical sintering can rupture the oxide shells between liquid metal microdroplets to restore conductivity [250], while Tang et al. demonstrated that applying approximately 10% strain can effectively rupture the oxide layer through a room‐temperature strain‐induced sintering process [251]. These techniques have significantly improved the flexibility and durability of liquid metal conductors (Figure 8d) [252]. Conventional activation methods typically necessitate multi‐step procedures and remain susceptible to liquid metal (LM) leakage over extended operational periods. To circumvent these limitations, LM conductors engineered with surface asymmetry and self‐encapsulating properties present a robust strategy for streamlining the fabrication of flexible circuits. For instance, Ahmed et al. fabricated an asymmetric self‐insulated stretchable conductor (aSISC) via interface‐assisted self‐assembly. By integrating a conductive bottom interface with an insulating top layer, this architecture intrinsically mitigates LM leakage [253]. In another approach, Seo et al. employed ligand‐binding chemistry to suppress oxide growth, driving the in situ sintering and vertical phase separation of LMs within a polymer matrix. This enables the one‐step printing of highly conductive, self‐packaged circuits while facilitating the scalable integration of multilayer architectures via vertical interconnect accesses (VIAs) [254]. Although structural and interfacial engineering effectively resolve fabrication and leakage constraints, hybridizing liquid metals with functional fillers provides an alternative pathway to further augment composite performance. Moreover, by carefully tuning the composition ratios of the constituents, the overall performance of the hybrid conductors can be effectively optimized. For instance, Kim et al. demonstrated that incorporating Ag nanowires into a liquid metal matrix significantly reduced surface resistance and improved mechanical durability under large strain. The resulting hybrid conductor maintained stable electrical properties under strains exceeding 800% and achieved a high conductivity of 7090 S/cm [62]. To further push the limits of mechanical compliance, researchers have focused on developing ultradeformable liquid metal‐based systems. For instance, Wang et al. designed an intrinsically stretchable electronic patch featuring liquid metal interconnects with a high conductivity of 3.8 × 10^4^ S/cm. This compliant device can withstand up to 400% tensile strain, allowing for conformal contact with dynamically moving organs with minimized mechanical mismatch to achieve stable epicardial electrophysiological monitoring [255].  Additionally, a bilayer structure comprising a copper thin film and liquid metal microcapsules has been constructed on elastomer substrates to achieve electrically self‐healing, ultrastretchable conductors. Upon mechanical damage, the localized stress concentration in the fractured copper layer selectively ruptures the underlying microcapsules to release the liquid metal healing agents, thereby ensuring robust electrical connectivity even under extreme deformations [256]. Furthermore, highly conductive liquid metal emulsion gels have also been developed to fabricate highly stretchable electronic circuits through automated 3D printing [257].

Despite these advantages, liquid metal conductors continue to face several critical challenges, including electromigration, corrosion, solidification, and biocompatibility concerns [258]. Electromigration refers to the phenomenon where liquid metals migrate toward the cathode under direct current conditions, leading to anode depletion and compromising the long‐term stability of the conductor [259]. Corrosion can occur when liquids undergo chemical reactions in aqueous or humid environments over extended periods, potentially damaging the protective oxide layer and resulting in the failure of conductive pathways [260]. Solidification remains a notable issue, as liquid metals, although prone to remaining in a supercooled liquid state, may still solidify under specific environmental or operational conditions, thereby affecting their mechanical and electrical properties [261]. Biocompatibility also presents a significant concern. While Ga‐based liquid metals are generally considered safe, their prolonged contact with biological tissues necessitates further in‐depth studies to ensure long‐term safety for biomedical applications [262].

Overall, metallic materials provide a robust foundation for the development of deformable conductors in flexible electronics. Although metal nanowires, nanoparticles, thin films, and liquid metals each offer distinct advantages and face specific limitations, these challenges can be effectively addressed through rational material design and the integration of composite structures. Such strategies can significantly enhance the conductivity, stretchability, and durability of the resulting conductors. Future research on metallic materials for deformable conductors is expected to focus on the synergistic interaction between different material components and the design of novel composite architectures. In particular, the incorporation of liquid metals with complementary functional materials holds considerable promise for overcoming existing performance bottlenecks and expanding application potential.

#### 2.2.3 Conductive Polymers

Conductive polymers (CPs) constitute a type of polymer capable of electrical conductivity, typically facilitating charge transport through their π‐electron conjugated systems. Their electrical conductivity is generally achieved through chemical or electrochemical doping. In addition to their tunable electrical properties, CPs exhibit excellent mechanical flexibility and ease of processing, making them uniquely advantageous for applications in deformable conductors. Under mechanical deformation, conductive polymers maintain structural integrity and electrical functionality, and they integrate well with various flexible substrates, thus offering substantial potential for stretchable electronic device development. Representative CPs include polyacetylene (PA), PANI, PPy, polythiophene (PT), and derivatives such as poly(3,4‐ethylenedioxythiophene) (PEDOT) and poly(3‐hexylthiophene) (P3HT) [263]. The main challenge in both academic and industrial research is to enhance the electrical conductivity of CPs without compromising their inherent stretchability. To address this, researchers have developed a range of optimization strategies, including advanced doping techniques and composite material design. The following section reviews recent advancements in performance optimization of CP‐based deformable conductors.

PANI is a widely studied conductive polymer that exhibits multiple oxidation and protonation states. In its oxidized state, PANI demonstrates metal‐like conductivity, while non‐conductive PANI becomes conductive through proton doping when exposed to protonic acids (HA) [264]. This enables PANI to exhibit tunable electrical conductivity between metallic and semiconducting behavior through acid doping control [265]. Despite its advantages, PANI suffers from intrinsic limitations. Its rigid molecular chains and strong interchain interactions lead to poor solubility in most solvents and render the material mechanically brittle. To enhance the mechanical and electrical performance of PANI, Horev et al. developed an innovative approach. They first treated polystyrene‐block‐polyisoprene‐block‐polystyrene (SIS) membranes with oxygen plasma to increase surface activity, followed by (3‐aminopropyl) triethoxysilane (APTES) treatment to enhance interfacial adhesion between the PANI layer and the substrate. The surface‐modified SIS membranes then underwent dynamic interfacial inverse emulsion polymerization under ultrasonication to graft PANI in acid‐doped conditions. This method enabled uniform PANI growth on SIS fibrous substrates, creating multifunctional deformable PANI conductors. Various doping acids (hydrochloric, oxalic, nitric, and sulfuric acids) were employed to control the morphology and conductivity of PANI. The resulting PANI‐based nanofiber sensors exhibited high breathability (10‐33 m/h) and could accurately distinguish bending and torsional stimuli across different angles (1–180°) and speeds (3–18 rpm) [63]. Zhang et al. employed ammonium persulfate (APS) as an oxidizing agent to initiate the polymerization of aniline, enabling the homogeneous dispersion of PANI within a modified natural rubber (NR) matrix to form continuous conductive pathways. Phytic acid (PA) was simultaneously introduced as both a dopant and a crosslinking agent during the polymerization process, significantly enhancing the structural integrity and continuity of the conductive network. The resulting deformable conductor exhibited remarkable self‐healing capabilities due to non‐covalent interactions (electrostatic interactions and hydrogen bonding) between PANI and NR. Notably, the deformable conductor maintained stable electrical performance under mechanical deformation, showing a minimal resistance variation of no more than 1.2% under 200% strain [266].

Conductive polymers have emerged as a research focus in flexible and stretchable electronics due to their unique combination of electrical conductivity and mechanical flexibility. Among these, PEDOT stands out as one of the most commercially successful conductive polymers, particularly when composited with poly(styrenesulfonic acid) (PSS) to form the classic PEDOT:PSS system [267, 268, 269]. PEDOT:PSS has a complex microstructure, typically characterized by the close interaction between PEDOT segments and PSS bundles. These interactions lead to the formation of colloidal gel particles in water. The morphology and electrical properties of PEDOT:PSS films can be precisely tuned through processing methods and additives, including secondary dopants [270]. Gao et al. improved the electrical and mechanical properties of PEDOT:PSS by introducing metal salts (e.g., Li^+^ and Fe^3^^+^) during the wet‐spinning process. These metal ions modulated the interactions between PEDOT and PSS chains through charge complexation. The study revealed that low‐valency metal ions like Li^+^ enabled PEDOT: PSS conductors to achieve a maximum elongation of 55%, the highest reported value for PEDOT: PSS. High‐valency ions such as Fe^3^^+^ promoted interchain crosslinking, thereby increasing mechanical strength while maintaining a conductivity of approximately 10 S/cm [64]. Beyond metal salt modifications, composite designs that integrate PEDOT:PSS with other functional materials have emerged as critical strategies for enhancing overall performance. For instance, Bessaire et al. synthesized continuous conductive PEDOT: PSS nanofibers using electrospinning. Since pristine PEDOT:PSS is nearly insulating, the nanofibers were subsequently treated with alcohols and glycerol as dopants. This treatment induced conformational changes in the polymer, shifting PEDOT chains from a benzoid to a quinoid structure, thereby improving electrical performance [271]. In another study, Wang et al. introduced a stretchability and conductivity enhancer to engineer a highly stretchable, transparent, and conductive PEDOT:PSS composite. The enhancer additively softened the PSS domains and facilitated partial aggregation of conductive PEDOT regions. As a result, the PEDOT:PSS‐based deformable conductor achieved outstanding electrical conductivity, exceeding 3100 S/cm at 0% strain and maintaining over 4100 S/cm at 100% strain [65].

PPy exhibits excellent flexibility and stretchability [272], enabling it to maintain electrical conductivity under various deformations. Lu et al. fabricated a conductive composite by coating PPy onto a PDMS sponge substrate via in situ polymerization. The deposited PPy layers formed on both the pore walls and framework of the sponge, significantly enhancing its conductivity. This unique structure facilitates the formation of new conductive pathways under compressive stress. When implemented in a gradient porous PPy/PDMS sponge‐based flexible pressure sensor, the deformable conductor demonstrated remarkable stability, exhibiting excellent conductivity and signal repeatability even after 10 000 compression‐release cycles [32]. PT has emerged as another promising conductive polymer due to its high doping capacity and electrochemical stability [273]. Chen et al. enhanced the compatibility of PT with soft polymers by doping it with anionic surfactants, which served to bridge the interfacial interactions between PT and PEG. The resulting PT‐based composite achieved a conductivity of 9.5 S/cm, demonstrating its suitability for integration into flexible electronic systems [274].

As a critical category of conductive materials for deformable conductors, conductive polymers demonstrate significant application potential due to their unique π‐conjugation systems and tunable doping characteristics, while maintaining both flexibility and electrical conductivity. Although recent advancements have been made in balancing electrical conductivity and mechanical performance, challenges remain in ensuring the stable conductivity of conductive polymers under high strain conditions and maintaining long‐term stability in extreme environments. Current research efforts have primarily focus on doping strategies and the integration of composite material technologies, which have improved the overall performance of conductive polymers to a certain extent.

#### 2.2.4 Composite Materials

Composite materials, engineered by integrating diverse material types such as carbon‐based substances, metals, and conductive polymers, offer the ability to combine the inherent advantages of each component while achieving synergistic enhancements through interfacial engineering. This hybridization strategy effectively improves electrical conductivity, enhances mechanical properties, and optimizes both processability and long‐term stability.

The composite strategy combining carbon materials with metal materials is a widely adopted approach to enhance both electrical conductivity and mechanical properties. Carbon‐based materials offer excellent electrical conductivity, chemical stability, and a high specific surface area, while metal materials provide superior conductivity and mechanical strength. The rational integration of these two components enables the composite to exploit the flexibility and surface activity of carbon materials alongside the high conductivity and strength of metals, thereby improving overall performance. For example, Patil and colleagues developed a deformable conductor as the conductive component in a flexible three‐electrode sensor system. This conductor consists of a composite material composed of nitrogen‐doped silk carbon (NSC) with AuNPs, referred to as AuNPs@NSC. The composite harnesses the high conductivity and electrocatalytic properties of AuNPs, along with the chemical stability and conductive of nitrogen‐doped carbon, making it a promising conductive material for use in flexible and high‐performance electronic devices [275]. Carbon quantum dots (CQDs), as an emerging class of carbon‐based nanomaterials, exhibit excellent electrical conductivity and chemical stability. Yu and colleagues developed a deformable conductor by integrating CQDs with AgNWs, which was subsequently employed in the fabrication of high‐performance flexible resistive switching devices [276]. Rahimi et al. utilized CO_2_ laser‐induced carbonization to produce a porous carbon‐Ag nanocomposite conductor. After 15 000 bending cycles, the resistance change of the composite was less than 0.6 Ω, in stark contrast to the pure carbonized polyimide, which exhibited a resistance change of up to 350 Ω. This result demonstrates that the addition of Ag nanoparticles improved electrical conductivity, achieving a surface resistivity of 0.02 Ω/sq (corresponding to a conductivity of 50 S/cm assuming a thickness of 1 cm), as well as significantly enhanced mechanical strength and durability [9]. Peng and colleagues fabricated a novel stretchable nanocomposite conductor by alternately depositing AgNWs and CNFs onto a polyurethane substrate using dip‐coating technology, forming a 3D segregated conductive network that was subsequently cured with polymethylsiloxan. In this composite, the AgNWs provide rigid conductive pathways, while the CNFs serve as flexible conductive bridges, creating a synergistic effect that maintains electrical conductivity under large deformations. The resulting composite conductor exhibits a high electrical conductivity of up to 16.6 S/cm and could withstand tensile strains up to 140%, while also maintaining stable conductivity under various deformation modes, including twisting and bending deformations [277].

The combination of metals and conductive polymers also demonstrated excellent performance [278], Wang and colleagues used PEDOT:PSS as a mediating material to improve the wettability of liquid metals on flexible substrates, enabling the fabrication of liquid metal‐based flexible electronic devices. The interaction between PEDOT:PSS and liquid metal comprised physical adsorption as well as chemical reactions, reflecting a synergistic interfacial behavior. PSS could react with oxides on the liquid metal surface, such as Ga_2_O_3_, to form a PEDOT:PSS‐Ga (III) composite, which softened the surface of the liquid metal as well as enhanced its adhesion to the PEDOT:PSS film. The synergistic combination of chemical and physical interactions ensures stable anchoring of the liquid metal to the PEDOT:PSS surface, facilitating the formation of continuous conductive pathways [279]. Ahmed and co‐workers developed a novel composite conductor based on liquid metal (composed of 75.5% gallium and 24.5% indium) and conductive polymer PEDOT:PSS. After printing, this composite could spontaneously self‐assemble into an asymmetric, self‐insulating deformable conductor. The bottom surface exhibited a high conductivity of up to 2089 S/cm, while the top surface remained electrically insulating. The composite demonstrates exceptional stretchability exceeding 800% and a mechanical modulus comparable to human skin, making it particularly suitable for wearable electronic devices that require conformal contact with the skin [280]. Namkoong and colleagues fabricated a deformable conductor by forming an interpenetrating network structure of AgNWs and PEDOT:PSS. The nanocomposite film exhibited a sheet resistance of approximately 70 Ω/sq, which was significantly lower than the 120 Ω/sq resistance of PEDOT: PSS films without AgNWs, demonstrating a substantial improvement in conductivity through the incorporation of AgNWs [281].

In summary, carbon‐based materials, metal‐based materials, and conductive polymers are commonly employed as core components in deformable conductors due to their unique physicochemical properties. Through the rational design of composite systems that integrate these materials, it is possible to combine their respective advantages. This approach enables the development of deformable conductors with high electrical conductivity, enhanced mechanical strength, and reliable structural stability, thereby supporting a wide range of applications in flexible and wearable electronic devices.

## 3 Circuit Interconnections

To enable the adaptive functionality of deformable electronics, advanced materials alone are insufficient, equally critical is the rational design of the conductive pathways. Specifically, the circuit interconnections and structural architectures that can accommodate diverse mechanical deformations without compromising electrical performance. Rather than focusing on active electronic components, this section provides an overview of the physical layout strategies for deformable conductors. We highlight how system‐level mechanical adaptability is achieved by patterning these conductors into specific geometric architectures.

### 3.1 Structural Design of Circuit Interconnections

The structural design of conductive traces within adaptive systems plays a critical role in balancing electrical conductivity and mechanical deformability. During stretching or deformation, these interconnects must maintain stable conductive pathways. Common structural configurations include island‐bridge structures, wavy structures, origami and kirigami designs, and crack‐based geometries. The island‐bridge structure integrates rigid functional units with deformable conductors, providing stable stretchability and good electrical conductivity. Wavy structures are well suited for applications involving dynamic deformation and highly elongation, offering enhanced flexibility but potentially underperforming under extreme deformation. Origami and kirigami designs provide increased structural adaptability and tunability, although they often involve greater fabrication and design complexity. Crack‐based structure focuses on maintaining conductivity under deformation but may fail under excessive stretching. By introducing geometric curvature or controlled dimensional modulation within conductive pathways, these structural strategies can effectively mitigate the trade‐off between conductivity and stretchability.

#### 3.1.1 Island‐Bridge Structure

The island‐bridge structure, a widely adopted design in flexible conductive circuits, is extensively utilized in adaptive conductive systems. Its fundamental configuration consists of multiple island‐like regions connected by bridging elements. In island‐bridge structures, the islands serve as stable platforms for rigid or functional electronic components, while the bridges act as stretchable interconnects that absorb mechanical strain and maintain electrical continuity [282]. This design effectively prevents the disruption of conductive pathways under strain, and its distinctive geometric configuration ensures excellent electrical stability and mechanical reliability under high‐strain conditions. The interconnecting sections in island‐bridge structures are commonly engineered in serpentine (Figure 9a) [283, 284, 285] or helical geometries, with each configuration exhibiting specific advantages depending on the application. For example, the serpentine structures are particularly suitable for highly stretchable electronic devices due to their efficient stress distribution and adaptability to deformation. In contrast, helical structures are better suited for high‐frequency deformation scenarios because of their mechanical stability and robust recovery capability. Therefore, investigating various interconnection strategies within the island‐bridge configuration and optimizing its design parameters are essential for advancing the performance of adaptive conductive systems [286].

**FIGURE 9:**
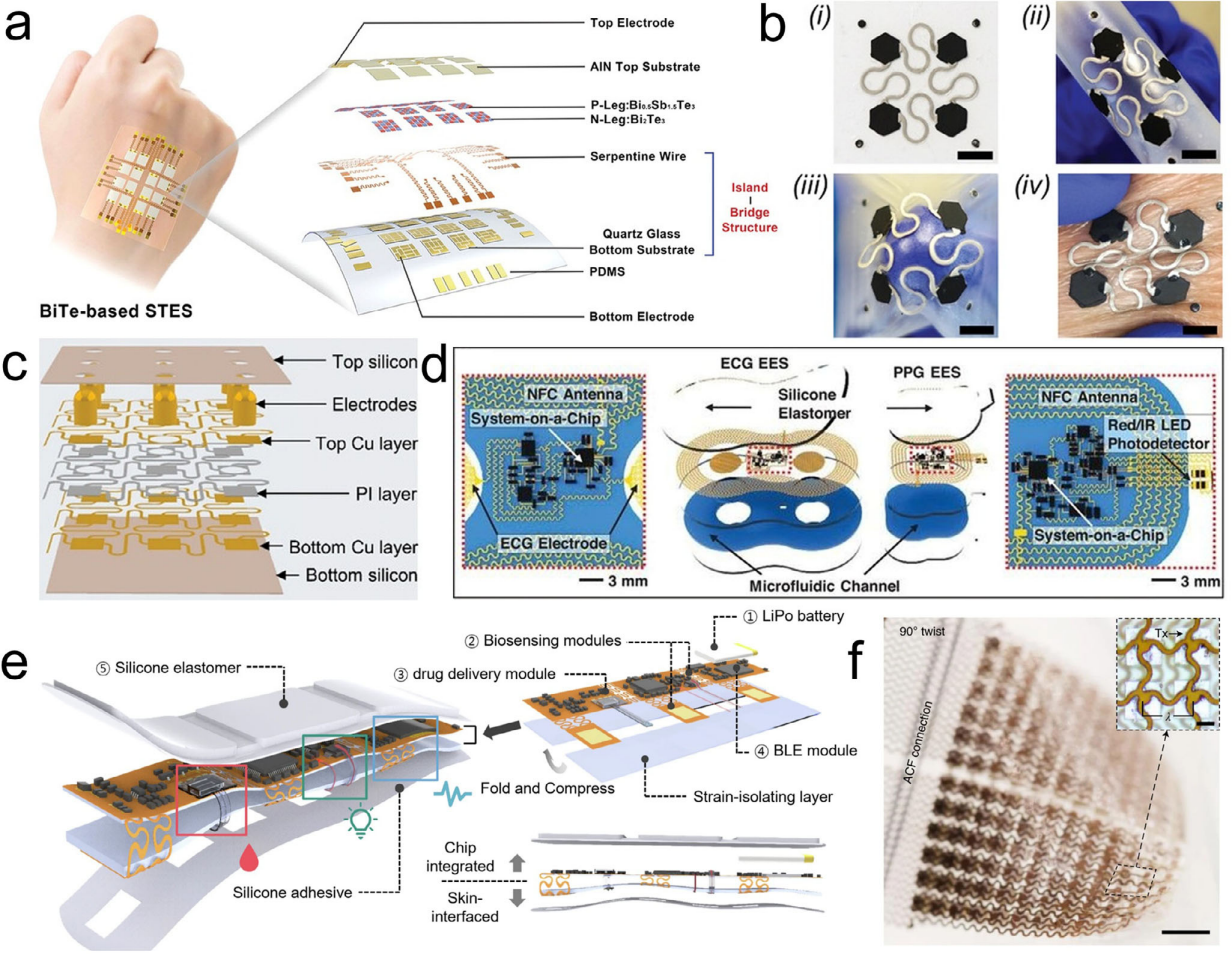
Schematic illustrations of the island‐bridge structure in flexible conductors. (a) Schematic illustration of the STES, showing the internal composition and structure in detail. Reproduced with permission [283]. Copyright 2023, Wiley‐VCH. (b) Optical Images of the printed Ag‐LM‐IB device under different mechanical deformations. Reproduced with permission [57]. Copyright 2020, Wiley‐VCH. (c) The detailed structure explosion view of SEFM‐RFT. Reproduced with permission [294]. Copyright 2025, Wiley‐VCH. (d) Schematic diagram of the internal circuit of a wireless battery‐free module. Reproduced with permission [296]. Copyright 2019, American Association for the Advancement of Science. (e) Integrating and folding process of folded multi‐modal device with multilayer components of chemical, optical, and electrical modalities. Reproduced with permission [297]. Copyright 2025, Springer Nature. (f) Optical image of a stretchable phased array twisted 90°, with bending and stretching of the interconnects. Reproduced with permission [298]. Copyright 2021, Springer Nature.

Serpentine interconnects typically consist of multiple periodically distributed units, each formed by two straight segments connected through a semicircular arc, producing a characteristic serpentine configuration. The key advantage of this structure lies in its ability to effectively distribute applied stress through geometric deformation, thereby preventing fracture or performance degradation of conductive traces under stretching [18]. Liu et al. demonstrated that by creating serpentine pathways between conductors, the island‐bridge structure allows conductors to bend and stretch without breaking. This design mimics the natural extensibility of human skin, where the serpentine pathways help distribute stress across the conductors, reducing stress concentration points and enhancing durability and reliability. They use a 170 µm thick PDMS layer as both the flexible substrate and triboelectric material, with copper traces that keep maximum principal strains below the 0.3% yield limit under roughly 8% stretching, 76° twisting, and bending down to a 5 mm radius [33]. Serpentine interconnects significantly improve the strain tolerance of the overall structure. For instance, Zhao et al. fabricated GaIn‐Ni‐based circuits with varying line widths on an elastic Ecoflex substrate using screen printing. By seamlessly integrating inherently stretchable liquid metal bridges with mechanically deformable serpentine structures, they achieved stable electrochemical performance even at 400% elongation [56]. Silva et al. fabricated interconnects composed of eutectic EGaIn particles blended with Ag composite ink, which retained conductivity under 800% strain (Figure 9b) [57]. Park et al. optimized the width and connection angle of serpentine traces, achieving only a 9% change in resistance at 300% strain, representing an exceptional performance metric [287]. The serpentine design provides bending flexibility not only within two‐dimensional planes but also accommodates substantial deformation in 3D [288]. This effectively reduces material strain, lowers overall structural stiffness, and enhances stretchability. Serpentine interconnects are typically fabricated using micromachining techniques such as photolithography [289], shadow mask evaporation [290], screen printing [291], and laser ablation [292]. These methods enable high‐precision patterning while maintaining good production efficiency. Lee et al. proposed a pre‐strain strategy in which the elastomer substrate is stretched prior to the integration of components and interconnects, thereby enhancing the conductor area coverage and overall stretchability of the device [293]. Zhou et al. first applied photolithography and wet etching to both the upper and lower copper layers to create the desired circuit patterns. Circular notches were etched into the top copper layer on certain island regions to achieve electrical isolation between these islands and the positive circuit pathway. Even under 30% tensile strain, the island‐bridge electrode array maintained stable electrical performance over 1000 stretching cycles (Figure 9c) [294]. In island‐bridge structures, serpentine interconnects are typically fabricated using metals such as Au, Cu, or Al, which exhibit excellent electrical conductivity. A key parameter in the design of serpentine interconnects is stretchability, which defines the maximum strain the structure can withstand without mechanical failure. Finite deformation models and finite element analysis (FEA) are often employed to predict the stretchability of such interconnects, including estimates of the maximum principal strain. Fan et al. emphasized that under large deformation conditions, linear models derived from small‐deformation theory can significantly overestimate their stretchability—by more than 50% in some cases. To address this, they proposed a simplified analytical model that incorporates nonlinear effects to improve predictive accuracy [295]. The advantage of the island‐bridge structure lies in its ability to combine high flexibility with mechanical and functional stability. The island regions serve as robust platforms for housing electronic components, ensuring their reliable operation under deformation, while the bridge sections are engineered to maintain electrical conductivity during stretching, thereby accommodating complex motions and mechanical strains. Chung et al. implemented this structure in a neonatal vital sign monitoring system, where the island regions support key components such as sensors, chips, and antennas, maintaining their stability even during the natural movements of newborns. The bridge sections, designed as serpentine metal thin films, allow the device to conform intimately to the skin without exerting pressure or causing damage during motion and deformation. To further reduce mechanical stress and enhance flexibility, microfluidic channels were introduced between the island and bridge regions. These channels, filled with a low‐conductivity ionic liquid, provide mechanical decoupling and minimize the impact of bridge deformation on the functional electronic components (Figure 9d) [296]. Building upon the same design principle, Lee et al. developed a multifunctional skin patch using an island‐bridge circuit configuration. Functional modules are interconnected by serpentine conductive traces, which are fabricated by electron‐beam evaporation of thin Cr and Au layers onto a polyimide substrate. The electronic components are protected by a strain‐isolating layer, such as silicone, forming a fully integrated adaptive conductive system. With this folded structure, the patch maintains flexibility and stretchability while supporting multimodal functionality (Figure 9e) [297]. Wang et al. employed a multilayer stretchable circuit fabrication strategy to construct a stretchable ultrasonic phased array. Each element in the array is individually connected through serpentine interconnects, enabling both independent control and system‐level stretchability (Figure 9f) [298]. Despite these advantages, serpentine interconnects in island‐bridge structures still face several challenges in practical applications. Densely packed serpentine designs may lead to stress concentration and mechanical failure, whereas loosely spaced serpentines can compromise electrical efficiency. Additionally, ensuring material compatibility during fabrication and controlling resistance variation under strain remain key challenges in optimizing serpentine structures.

Helical interconnect designs, as a critical component within island‐bridge structures, enhance the stretchability of conductors by incorporating spiral‐shaped conductive pathways. Compared to conventional serpentine configurations, helical structures offer distinct advantages in stress distribution and mechanical extensibility. Two‐dimensional (2D) helical patterns are typically generated by winding a circular or helical curve from a fixed point. Such geometries are frequently found in nature, as exemplified by golden spirals and nautilus shells, and are increasingly employed in the design of flexible electronic devices [13]. When subjected to external mechanical forces, 2D helical structures dissipate strain energy through out‐of‐plane deformation. This design effectively reduces stress concentration and provides superior stretchability compared to traditional serpentine interconnects at equivalent coverage areas and contour lengths [299]. Moreover, once the external force is removed, the helical structure quickly returns to its original configuration, demonstrating a high degree of reversible deformation. These characteristics make helical interconnects particularly advantageous for applications involving adaptive conductive systems that require repeated stretching and recovery cycles. Lv et al. used FEA to study Archimedean spiral interconnects, showing that they could stretch elastically up to 250% and reach 325% strain before breaking [299]. To further enhance the performance of spiral interconnects, 3D helical structures have been increasingly adopted in island‐bridge designs. Unlike the 2D serpentine structure, which tends to concentrate strain under stretching, the 3D helical structure effectively mitigates stress concentration through subtle mechanical coupling and uniform spatial deformation [13]. In the fabrication of 3D helical interconnects, a pre‐stretched elastomer substrate is typically employed to transfer filamentary serpentine traces with predefined bonding sites onto the substrate. When the pre‐strain in the substrate is released, the serpentine precursors experience compressive stress, triggering in‐plane and out‐of‐plane translational/rotational motions that ultimately form 3D helical structures with bending and twisting characteristics. This configuration achieves exceptional stretchability and mechanical robustness without compromising conductivity [300, 301, 302]. Jang et al. successfully implemented 3D helical interconnects in bio‐integrated electronic circuits, enabling devices to accommodate natural human body motion and deformation, withstanding biaxial stretching up to 50% [79]. Helical interconnects are prestructured in a spiral configuration oriented perpendicular to the substrate, enabling them to deform in a spring‐like manner during stretching to alleviate stress concentration. This architecture maintains high electrical conductivity as well as significantly enhances the reversible deformability of the conductor under complex mechanical strains such as multiaxial stretching, bending, and twisting.

#### 3.1.2 Curved Structures

Curved structures represent another crucial approach in the structural design of adaptive conductive systems. Owing to their excellent adaptability to mechanical deformation, these structures can effectively distribute stress under high‐strain conditions and prevent the disruption of conductive pathways. Unlike the rigid island connections of the island‐bridge structures, curved structures maintain conductivity stability during stretching through periodic or continuous curve designs (wavy [303, 304, 305] and helical [306] structures are common curved structures). Wavy structures typically exhibit periodic undulating patterns, offering superior deformability and uniform stress distribution. Similarly, helical rotating structures further enhance stretchability and deformation adaptability.

Unlike conventional straight‐line configurations or island‐bridge structures, wavy structures introduce periodic sinusoidal‐like geometric variations along conductive pathways through designed periodic undulations [307]. The wavelength (distance between adjacent crests or troughs) and amplitude (vertical distance between crest and trough) serve as critical design parameters [308]. When external stress is applied, each periodic segment of the wavy structure deforms uniformly, enabling homogeneous stress distribution across the entire structure. For example, Wu et al. fabricated a partially delaminated periodic wavy geometry by attaching a AgNW/PDMS composite film onto a pre‐stretched elastomer substrate and subsequently releasing. Upon strain release, the system formed partially delaminated periodic wavy geometries. During stretching, this wavy configuration effectively reduced local strain in the AgNW composite layer, maintaining excellent conductivity with less than 3% resistance change at 100% strain [309]. A larger bending radius further contributes to structural integrity by minimizing the risk of material failure during deformation, thereby improving the long‐term stability and durability of the conductor. Wavy structures play a significant role in improving the hysteresis performance of conductors during stretching. For example, Chen and collaborators developed an adaptive conductor with wavy microchannel designs that effectively constrained the viscoelasticity of the elastomer, thereby enhancing hysteresis performance and enabling the conductor to withstand strains up to 320% [310]. Ren et al. constructed wavy‐structured conductive networks using MXene and CNTs as conductors on TPU electrospun fiber membranes. The resulting wavy TPU fiber network exhibited low hysteresis and excellent stretchability during vertical deformation [311]. Wang et al. employed screen‐printing technology to pattern silver electrode grids and carbon electrode templates onto a styrene‐ethylene/butylene‐styrene substrate. The electrodes were designed in a wavy configuration, which enhances stretchability and flexibility while maintaining electrical conductivity. The device remained fully functional under 60% strain, exhibited no structural deformation at 15% strain, and showed no delamination at 30% strain (Figure 10a) [303]. Fabrication of wavy structures typically relies on precision microfabrication techniques to achieve high‐precision on substrates, ensuring feasibility and consistency for large‐scale manufacturing. Li et al. employed nanoimprint technology to fabricate wavy metallic interconnects on fiber‐embedded PDMS substrates [312]. Similarly, Yoon et al. utilized screen printing to produce wavy and horseshoe‐shaped Ag nanoparticle circuits on polyurethane substrates, which maintained stable conductivity even under 20% strain [313]. Integration with 3D printing further enhances the design flexibility of wavy architectures. Bao et al. used direct ink writing (DIW) 3D printing technology to fabricate wavy PANI composite networks that can withstand up to 550% strain [314]. Additionally, Bowden et al. deposited metal thin films as conductive layers onto thermally expanded PDMS substrates. Upon cooling, the PDMS contracted, generating compressive stress that induced buckling in the metal film, thereby forming a wavy structure [315]. Laser carving technology can significantly reduce bending stiffness and stress concentration by modifying the curvature of wavy circuit structures, thereby facilitating deformation at bending points and enhancing the overall flexibility of the circuit. Yun et al. employed finite element modeling to investigate the effect of different laser carving depths on the maximum stretchability of wavy circuits. The simulation results indicated that an optimal carving depth can markedly improve the stretchability, whereas excessive carving may lead to structural failure (Figure 10b) [304]. The wavy circuit structure developed by Yuan et al. was fabricated using a surface energy‐directed assembly technique. This process leverages the contrast in surface energy on a functionalized substrate to selectively guide the assembly of nanomaterial solutions into hydrophilic regions, thereby forming high‐resolution serpentine electrodes (Figure 10c) [316].

**FIGURE 10:**
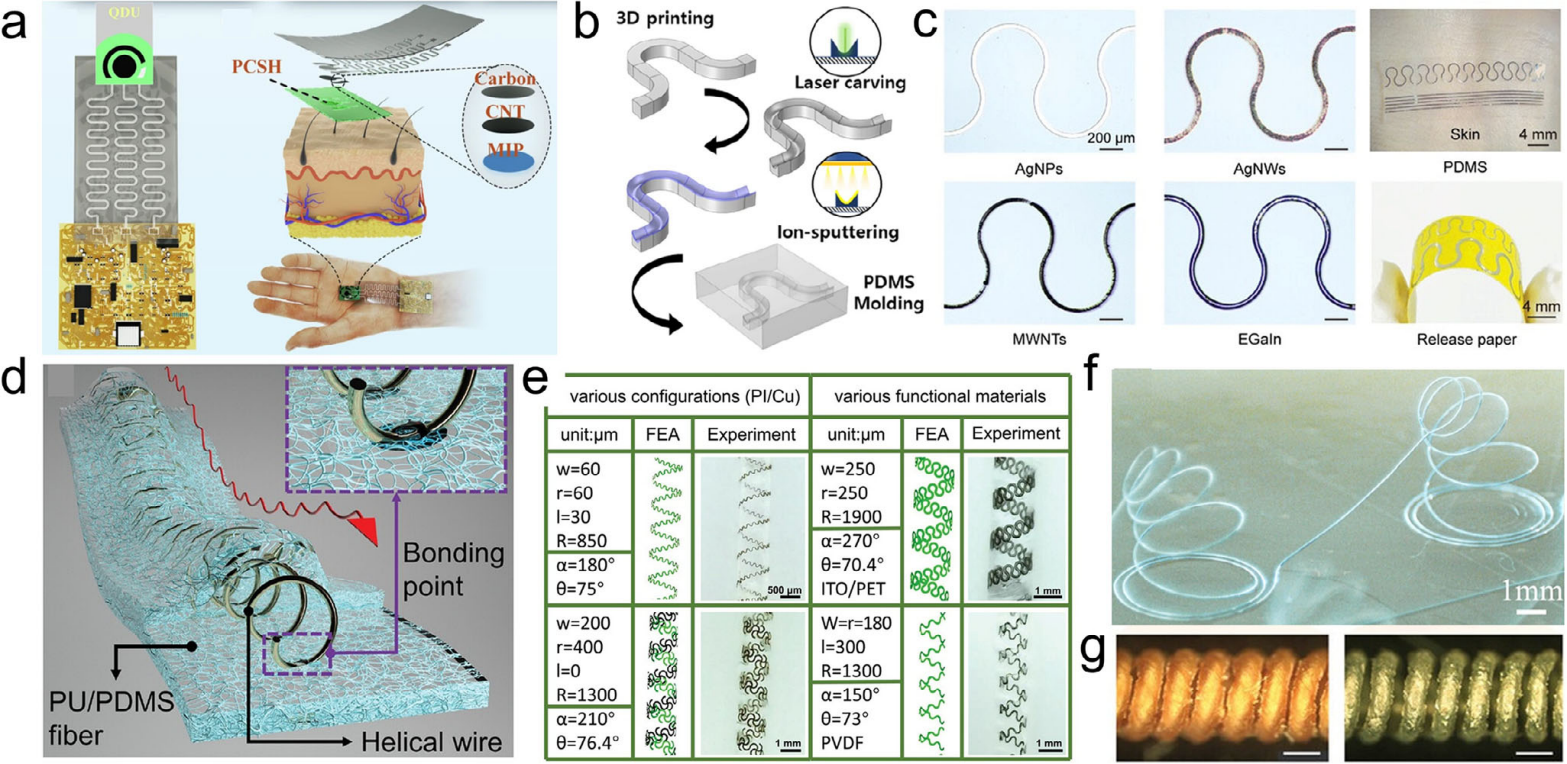
Schematic illustrations of the curved structures in adaptive conductive systems. (a) Schematic diagram of the specific circuit structure and the sensing mechanism in which cortisol diffuses from accumulated palm sweat through the hydrogel to the MIP electrode. Reproduced with permission [303]. Copyright 2024, Springer Nature. (b) Scheme for fabricating laser carved wavy circuit sample. Reproduced with permission [304]. Copyright 2022, Springer Nature. (c) Schematic diagram of the wavy electrode. Reproduced with permission [316]. Copyright 2025, Wiley‐VCH. (d) Schematic diagram of the spiral circuit. Reproduced with permission [46]. Copyright 2023, Wiley‐VCH. (e) FEA predictions and optical micrographs of hierarchical 3D wavy‐helix combination with various geometric configurations and diverse functional materials. Reproduced with permission [319]. Copyright 2023, Wiley‐VCH. (f) Spatial helices formed by accelerated warping under 85°C, showing the repeatable and stable shape. Reproduced with permission [320]. Copyright 2024, Taylor and Francis Ltd. g) Schematic diagram of the fabrication of the spiral structure. Reproduced with permission [321]. Copyright 2023, Wiley‐VCH.

These configurations facilitate effective stress dispersion during mechanical deformation, thereby preventing localized stress concentrations and potential conductor failure. For instance, Jang et al. fabricated high‐aspect‐ratio serpentine metal structures with wavy bending profiles to achieve both mechanical stretchability and optical transparency. In this context, a high aspect ratio refers to the height of the wavy profile being significantly greater than its width. The resulting structure exhibited excellent stretchability, maintaining stable electrical resistance under 100% tensile strain [317]. Sadri et al. utilized inkjet printing to fabricate conductors on paper substrates. During stretching, the wavy segments progressively unfolded, effectively increasing the conductor length while avoiding mechanical failure [80]. Another notable morphological design is the spiderweb‐like structure, as demonstrated by Chae et al. using laser processing technology. These geometrically engineered designs achieve stretchability by evenly distributing mechanical stress through curved pathways. This efficient stress dissipation helps preserve electrical continuity under deformation [318]. As a structural design strategy, wavy configurations offer substantial advantages in terms of stress distribution, deformation adaptability, and compatibility with scalable manufacturing techniques. These characteristics make wavy structures a highly promising approach for adaptive conductive systems in a broad range of application domains.

Helical structures represent a unique curved configuration that exhibits performance in the design of adaptive conductive systems. Distinct from wavy structures that employ linear or periodic curves to accommodate strain, helical structures provide superior stretching stability while preserving conductivity through their 3D rotational geometry. The essential advantage of these structures originates from their 3D morphology, where each coil can simultaneously extend axially during stretching while undergoing adaptive radial deformation, enabling effective stress distribution and minimized stress concentration through rotating and expanding. For example, Li et al. were inspired by accordion lanterns and designed a helical interconnect structure composed of two electrospun PU fiber membrane substrates and a helical metal fiber conductor. In this system, the helical metal fiber functioned as a stable conductor, while the PU fiber membranes and a PDMS layer provided a self‐adhesive substrate. The metal fiber was fixed onto the PU membrane and bonded with PDMS to form a helical structure that maintained stable conductivity under strains of up to 150%. The 3D helical configuration effectively dispersed mechanical stress during deformation, ensuring the integrity of the conductive pathways (Figure 10d) [46]. Geometric parameters of the helical structure, such as pitch, bending angle, and helical radius, directly influence its stretchability and mechanical stability. For instance, larger pitch and smaller bending angles tend to enhance stretchability, whereas smaller radii and tighter helices may improve the structural stability under high strain. By fine‐tuning these parameters, it is possible to achieve an optimal balance between stretchability and conductivity. By combining serpentine and helical configurations from wavy architectures, Yan et al. developed a 3D conductive circuit. This hybrid design enabled deformation exceeding 200% along the x, y, and z axes. Under strain, the serpentine segments unfolded to increase the length of the conductor, while the helical segments contributed additional elongation through rotational extension (Figure 10e) [319]. The helical structure extends within the plane as well as possesses a certain vertical height, forming a spring‐like three‐dimensional configuration. This spatial arrangement enhances the flexibility and stretchability of the conductor, allowing it to deform freely in three‐dimensional space without being constrained as planar conductors often are. Guo et al. employed highly conductive silver wires to construct such structures, which maintained nearly constant resistance under 40% strain. Upon release of the applied force, the conductors quickly returned to their original shape without exhibiting noticeable residual deformation (Figure 10f) [320]. Wu et al. fabricated hydrogel yarns by blending aramid nanofibers (ANFs) and PVA using a temperature‐controlled wet spinning strategy. These yarns were then twisted and coiled into helical shapes, followed by coating with AgNWs to establish conductive pathways. In addition to excellent conductivity and stretchability, the helical conductors also demonstrated high sensitivity to environmental temperature variations. This temperature‐sensing capability is primarily attributed to changes in the stacking density of AgNWs at different temperatures (Figure 10g) [321].

Curved structures possess exceptional capabilities for stress distribution and deformation accommodation, enabling adaptive conductive systems to achieve superior mechanical resilience and functional stability in dynamically changing environments. By employing periodic or continuous curved geometries, such structures effectively alleviate localized stress concentrations and minimize the risk of mechanical failure, thereby addressing the common limitations of conventional straight‐line conductors under tensile strain. This structural strategy not only enhances stretchability but also ensures the preservation of electrical conductivity during repeated or extreme deformations, making it highly advantageous for advanced flexible and stretchable electronic applications.

#### 3.1.3 Origami and Kirigami Structures

In recent years, origami and kirigami structures [322], inspired by traditional art forms, have been widely adopted as innovative design strategies in the structural engineering of adaptive conductive systems. These two approaches utilize precise geometric manipulations through folding and cutting, respectively, to achieve exceptional deformation adaptability. The designed structures enable conductors to maintain both electrical functionality and mechanical stability under various strain conditions.

Origami structures [323, 324] achieve mechanical deformability and recoverability through predetermined folding lines and crease patterns, allowing the material to undergo repeated deformations while maintaining overall structural integrity. Unlike kirigami, origami primarily relies on folding rather than cutting, with the resulting creases preserving structural integrity through multiple deformation cycles. For example, Song et al. employed PDMS as a substrate and introduced protruding PDMS walls on a pre‐stretched surface to support silicon nanomembranes, thereby creating microscale origami‐inspired structures (Figure 11a,b) [325]. Na and collaborators developed a reversible self‐folding origami configuration by integrating micropatterned conductive stripes on the top and bottom layers of a trilayer photo‐crosslinked polymer film [326]. The design principle of origami structures lies in portioning materials into multiple folding units through predetermined fold lines and creases. These units can deform independently under mechanical stress while preserving the overall structural stability. This approach provides excellent stretchability and deformability without requiring additional materials or structural complexity. For instance, Chen applied origami patterns onto paper substrates integrated with conductive polyester tape, resulting in a conductive circuits with a stretchability of up to 30.4% [67]. Among various origami patterns, Miura‐Ori configuration with a stretch ratio exceeding 1300% is currently the most widely adopted [327]. The Miura‐Ori crease pattern can be represented as a quadrilateral mesh composed of vertices, lines, and facets [328]. This specific crease arrangement creates a highly deformable structure capable of effectively distributing stress and maintaining stability under load. Li et al. implemented the Miura‐Ori design on a flexible printed circuit board (FPCB), enabling it to transition from a fully unfolded state to a completely folded state with a stretchability of up to 33.3% [329].

**FIGURE 11:**
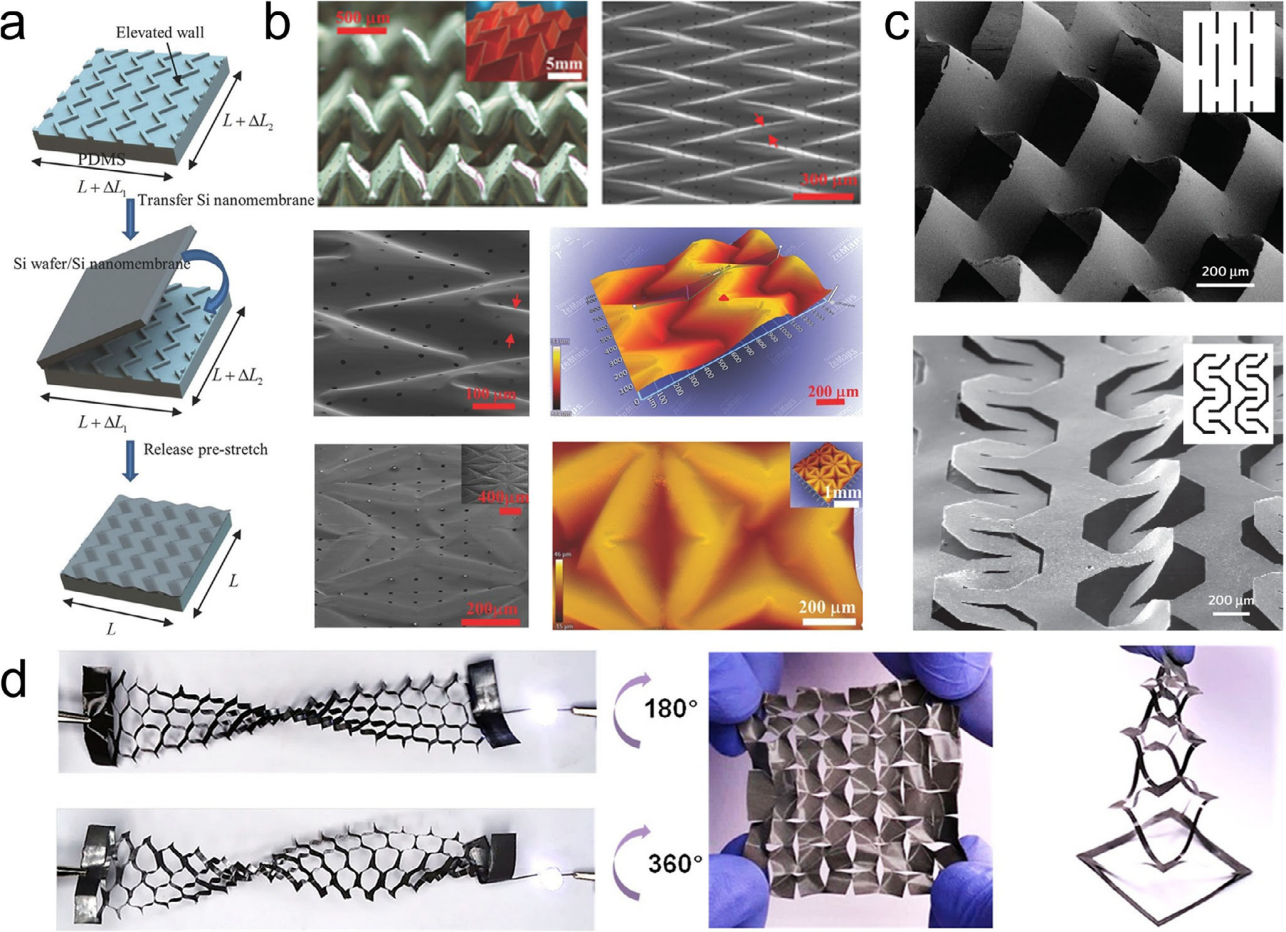
Schematic illustrations of origami and kirigami structures in flexible conductors. (a) Schematic illustration of using elevated PDMS walls to suspend Si NM and releasing the pre‐stretch to generate designated microscale origami patterns. Reproduced with permission [325]. Copyright 2016, Wiley‐VCH. (b) A photography of Miura‐ori patterns. Reproduced with permission [325]. Copyright 2016, Wiley‐VCH. (c) Two examples of microscale kirigami patterns in GO‐PVA nanocomposites after photolithography. Reproduced with permission [35]. Copyright 2015, Springer Nature. (d) Three types of kirigami patterns, including uniaxial, biaxial, and square spiral patterns in their original and stretched states. Reproduced with permission [68]. Copyright 2022, American Chemical Society.

Kirigami structures [330, 331] achieve enhanced deformability by incorporating precisely designed cuts that relieve localized mechanical stress, enabling significant mechanical deformation without compromising electrical conductivity. Compared to origami structures, kirigami designs provide greater deformation capacity while maintaining continuous and stable conductive pathways under extreme strain conditions. Wang et al. fabricated kirigami‐patterned circuit by laser‐cutting CNT networks on elastomeric PDMS substrates. These structures exhibited exceptional stretchability up to 430% elongation, with minimal resistance variation under 380% strain, demonstrating excellent electromechanical stability [34]. Similarly, Park et al. created kirigami patterns on PI films via laser processing, achieving stable performance at 80% strain [332]. Guan et al. employed air‐water interface assembly to fabricate freestanding polymer nanosheet conductors. By introducing kirigami patterns, they enhanced the stretchability of material from approximately 20% to an unprecedented 2000% strain while preserving conductive functionality [333]. The primary advantage of kirigami designs lies in their ability to enhance stretchability by reducing local stiffness, making them particularly well‐suited for applications requiring exceptional mechanical adaptability. Experimental studies by Shyu et al. demonstrated that while unpatterned materials typically fail at approximately 4% strain, kirigami‐patterned materials can achieve remarkable strain limits up to 370% (Figure 11c) [35]. By introducing well‐defined cuts, kirigami designs create predetermined pathways for both mechanical strain and electrical conduction. Li and colleagues developed kirigami‐based liquid metal‐paper conductive circuits by introducing precisely designed cuts that create predetermined strain distribution pathways and conductive networks. This innovative design maintained nearly constant electrical resistance during tensile deformation (0%–105% strain), torsional testing (−360° to 360°), and bending experiments (0° to 90°), while achieving a conductivity of 3.1 × 10^3^ S/cm (Figure 11d) [68]. The performance of kirigami structures can be precisely tailored by adjusting the geometry and spatial distribution of the cuts. This design flexibility allows for substantial deformation capacity while ensuring maintained conductivity and structural stability. Through careful optimization of cutting patterns, kirigami structures achieve an optimal balance between mechanical stability and electrical performance, making them highly adaptable to various application requirements.

Origami structures achieve significant deformation within confined spaces through folding of materials, while kirigami structures enhance deformability by releasing localized stress through strategically placed cuts. Both origami and kirigami techniques obtain shape‐morphing structures through intricate design spaces involving numerous mechanical nonlinearities and geometric constraints [327]. Through the implementation of these structural strategies, it is possible to substantially improve the stretchability and adaptability of conductive circuits without compromising their electrical conductivity. These characteristics make origami‐ and kirigami‐inspired designs particularly well‐suited for flexible electronic devices operating under high‐frequency deformation, long‐term mechanical cycling, and complex stress environments.

#### 3.1.4 Crack Structure

Cracks in conductors are traditionally regarded as defects that compromise device integrity or lead to increased electrical resistance under mechanical deformation. However, with precise design and control, cracks can be transformed into a functional advantage. Unlike conventional conductors, in which uncontrolled cracks disrupt conductive pathways, a well‐engineered crack configuration can establish an adjustable and recoverable conductive network. This approach enables the conductor to maintain stable conductivity even under substantial strain.

In conductive circuits, the formation of cracks is directly associated with applied strain. While intentionally introduced crack structures may induce localized fractures during stretching, they do not completely disrupt the overall conductivity of the conductor. Instead, conductivity is maintained through a “fracture‐reconnect” mechanism that preserves the continuity of conductive pathways. By precisely controlling crack depth, density, and geometric morphology on the substrate, localized disconnections of conductive paths can be achieved during deformation. However, the undulating morphology at crack edges enables adjacent conductive segments to reconnect under tensile stress, thereby effectively reducing resistance and maintaining conductive performance (Figure 12a) [334]. For example, Park et al. created a conductive system by depositing platinum (Pt) layers on PUA substrates. Through mechanical bending and stretching treatments, controlled crack patterns were generated. Further tensile strain allowed precise adjustment of crack depth without altering other parameters, thereby enhancing the sensitivity of the resulting sensor (Figure 12b) [69]. Regarding the control of crack density and depth, Kong and collaborators deposited an Al layer onto a PDMS substrate, followed by oxygen plasma treatment to form an ultra‐thin silicon oxide (SiO_x_) interlayer. Cracks were subsequently introduced into this trilayer Al/SiO_x_/PDMS structure. By adjusting the oxygen plasma exposure time and the thickness of the Al layer, they were able to precisely control both the density and depth of the cracks. The resulting cracks exhibited uniform dimensions and were confined within boundaries, producing stable circuit patterns with well‐defined edges. The fabricated conductive circuit maintained stable structural and electrical performance under 125% omnidirectional strain for 3000 repeated cycles (Figure 12c) [36]. Wang et al. fabricated a conductive circuit by depositing multilayer CNT thin‐film conductor on PDMS substrates. During a pre‐stretching process, networked cracks were generated within the CNT films. These cracks dynamically opened and closed during subsequent mechanical stretching. The conductor maintained electrical conductivity after 1500 stretching cycles at 20% uniaxial strain [335]. Under high‐strain conditions, such crack‐based structures not only facilitate uniform stress distribution but also prevent significant disruption to local conductive pathways, thereby ensuring the overall continuity and stability of electrical performance.

**FIGURE 12:**
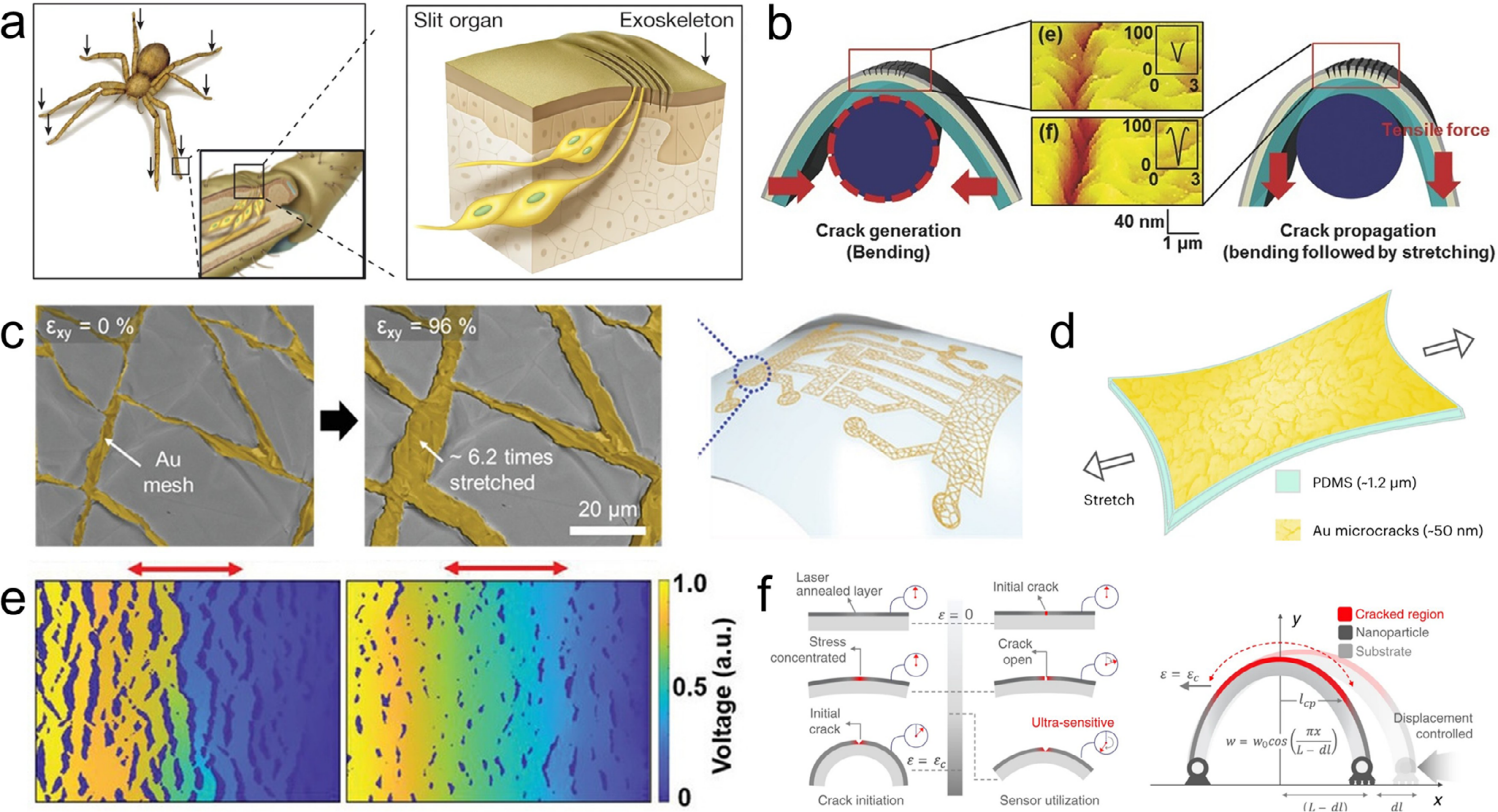
Schematic illustrations of crack‐based structures in conductive circuits. (a) The spider has highly sensitive organs located on its leg joints for the detection of external forces and vibrations. The slits are connected to the nervous system to monitor vibrations. Reproduced with permission [334]. Copyright 2014, Springer Nature. (b) Initial crack generation by bending, and crack propagation by additionally applied stretching. Reproduced with permission [69]. Copyright 2016, Wiley‐VCH. (c) SEM image of the Au grid showing before and after stretching. Reproduced with permission [36]. Copyright 2021, Wiley‐VCH. (d) Structural illustration of the PDMS‐Au conductor, where PDMS has a thickness of ∼1.2 µm and the Au layer with microcracked morphology has a thickness of ∼50 nm. Reproduced with permission [336]. Copyright 2022, Springer Nature. (e) Potential distribution of PDMS_0.9_‐IPDI stretchable electrodes at 100% tensile strain based on conventional single‐layer microcrack structure and double‐microcrack coupling structure, respectively. Reproduced with permission [339]. Copyright 2023, Wiley‐VCH. (f) Schematic illustration and modeling parameters of displacement‐controlled bending environment. Reproduced with permission [340]. Copyright 2020, Springer Nature.

Inspired by the biological crack structures found in spider organs, crack‐based conductive circuits have demonstrated outstanding performance in flexible electronic devices [334]. By introducing microcracks on the surface of the conductive layer, [238] electrical conductivity can be effectively maintained and, in some cases, even enhanced. The dynamic formation and reconnection of these cracks enable high repeatability and mechanical durability, making such materials particularly suitable for applications requiring both high sensitivity and large strain tolerance. In particular, the tunability of the crack geometry allows the conductor to sustain long‐term, high‐performance operation under cyclic deformation. For instance, Au films with engineered microcracks exhibit remarkable stretchability. Their high deformability is attributed to the ability of the metal network to twist and deflect out of the plane during substrate elongation, while still maintaining adhesion to the soft substrate (Figure 12d) [336]. This structural configuration enables the Au film to undergo minimal internal strain and deform elastically under mechanical loading. The microcrack‐patterned conductive circuits developed by Lacour et al. further validated the effectiveness of this approach. Their results showed that although resistance increased during stretching, it returned to a relatively stable baseline after each cycle. The system achieved reliable operation under strains of up to 32%, demonstrating robust mechanical resilience and electrical recovery [337]. Sun et al. deposited CNTs and AgNWs onto electrospun TPU fiber mats to fabricate conductive films. Microcracks were introduced through pre‐stretching, enabling the resulting conductor to withstand strains exceeding 450% while maintaining electrical functionality [338]. Building upon the concept of single microcrack structures, Yang proposed a dual‐microcrack coupling strategy. In this design, two Au microcrack films were integrated to form enhanced conductive pathways, significantly enhancing stretchability and preserving conductivity under strains of up to 200% (Figure 12e) [339]. Kim et al. employed a laser‐induced technique to generate microcrack‐based circuit structures within a AgNP layer. This laser processing method enables precise control over crack formation, allowing for the adjustment of crack density and depth. The gap between cracks varies continuously with applied strain, resulting in a corresponding continuous change in electrical resistance (Figure 12f) [340]. Common methods for creating microcrack structures in conductive circuits include pre‐stretching [341], deposition [342], and laser ablation processes [340]. These approaches enable the control of crack density, depth, and orientation, ensuring the creation of reliable and stretchable conductive networks with excellent mechanical and electrical performance.

Although crack structures in circuits offer excellent conductivity and stretchability, the configuration design still presents several challenges. One of the primary concerns is the random distribution of cracks, which can lead to interruptions in the conductive pathways and compromise electrical performance. Therefore, it is essential to precisely control the crack shape, depth, and spatial distribution during the design and fabrication process. Irregular or uncontrolled crack formation may result in severe damage to the conductive network, ultimately offsetting the intended mechanical and electrical benefits. To address this issue, researchers have proposed various strategies to optimize crack design, including, for example, employing multilayer composite materials [343], incorporating self‐healing methods, or implementing structural designs [344]. These advancements contribute to both the enhancement of mechanical reliability and the long‐term stability of crack‐based adaptive conductive systems.

### 3.2 Fabrication of Circuit Interconnections

The fabrication process of the circuits plays a pivotal role in optimizing their performance, directly influencing their applicability in flexible electronic systems. To ensure precise placement of conductive components on elastic substrates, a variety of fabrication techniques have been developed. Beyond specialized or emerging methods such as laser processing, electrospinning, and microfluidic injection, widely adopted and representative strategies include deposition, printing, and etching. The selection of each method depends on material properties as well as the application demands, particularly in balancing large‐scale production with high‐performance stability.

#### 3.2.1 Deposition

Deposition is a widely adopted fabrication technique for conductive circuits, enabling the uniform formation of thin films or intricate conductive networks on flexible substrates (Figure 13a) [345]. This method offers high controllability, excellent reproducibility, and scalability, making it well‐suited for large‐area and high‐performance device production. Common deposition approaches include CVD, PVD, electrochemical deposition (ECD), and spray deposition. These techniques can be categorized into vapor‐phase deposition (CVD and PVD) and liquid deposition (ECD and spray deposition). Vapor‐phase deposition relies on reactions between gaseous precursors and substrate surfaces for material deposition, whereas liquid‐phase deposition utilizes liquid precursors that undergo solvent evaporation or current‐driven deposition onto substrates.

**FIGURE 13:**
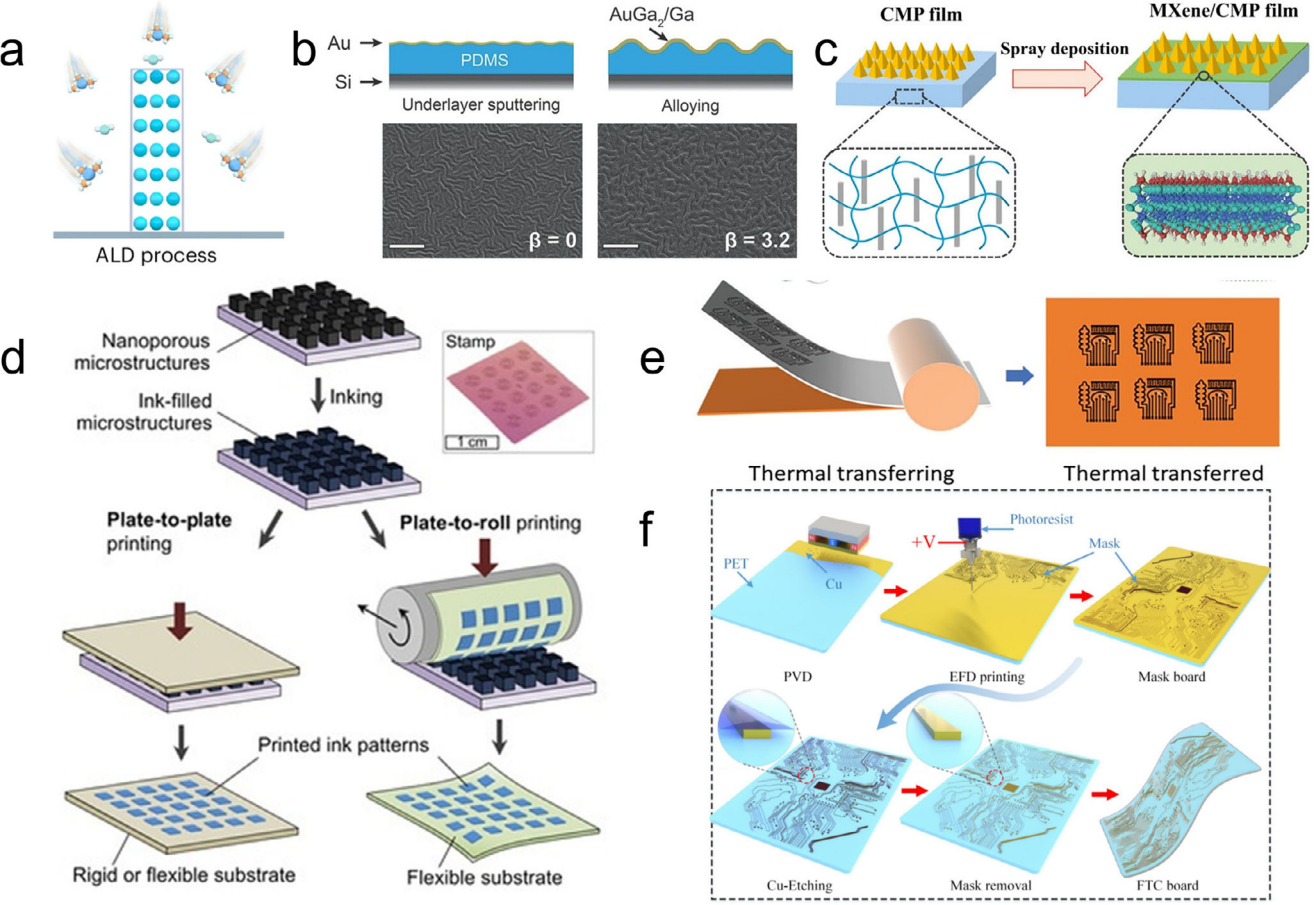
Schematic illustrations of circuit fabrication process. (a) Schematic diagram of ALD process to conformally deposit ultrathin Al_2_O_3_ film on vertically grown metals. Reproduced with permission [345]. Copyright 2024, Springer Nature. (b) Growth of biphasic gold‐gallium thin films on a PDMS membrane. Reproduced with permission [351]. Copyright 2016, Wiley‐VCH. (c) The spray deposition method was used to uniformly deposit MXene dispersion on the surface of the CMP film. Reproduced with permission [356]. Copyright 2024, American Chemical Society. (d) Schematics of the printing procedure. Reproduced with permission [373]. Copyright 2016, American Association for the Advancement of Science. (e) Schematic diagram of the transfer printing process. Reproduced with permission [394]. Copyright 2025, John Wiley and Sons Ltd. (f) A schematic of the additive/subtractive composite manufacturing method. Reproduced with permission [393]. Copyright 2024, Elsevier.

CVD [181, 346, 347] is a thin‐film fabrication technique that utilizes gaseous precursors to undergo chemical reactions on heated substrate surfaces. During the CVD process, the gaseous precursors react with the substrate surface through thermal activation or plasma excitation, forming solid materials that deposit onto the substrate. This process typically requires specific temperature conditions (200–1000°C) and low‐pressure environments, with commonly used reaction gases including methane and hydrogen. CVD finds extensive applications in preparing metallic and carbon‐based materials, particularly for depositing graphene and CNTs. A representative example is demonstrated by Chen et al., who employed CVD to grow graphene films on Ni foam templates. Through subsequent processing steps, free‐standing graphene foam was obtained and transferred onto PDMS prepolymer substrates, resulting in GF/PDMS composite conductive circuits [200]. By precisely controlling the CH_4_ concentration during CVD, the researchers effectively tuned key parameters of the graphene foam, including layer number, specific surface area, and density, showcasing the exceptional flexibility of CVD in regulating graphene microstructures.

PVD [348, 349] is a versatile thin‐film coating technique that transforms solid materials into the vapor phase through physical processes, followed by deposition onto substrate surfaces. This method enables cost‐effective deposition of various inorganic materials [350] without involving chemical reactions. Common PVD techniques include thermal evaporation and sputter deposition, where source materials are vaporized via heating or plasma sputtering under low‐pressure conditions, subsequently condensing on substrates to form thin films. PVD is particularly suitable for depositing metals, alloys, and certain conductive polymers. For instance, Hirsch et al. first sputtered a gold film onto a PDMS substrate to serve as an alloying layer, and then thermally evaporated gallium onto the surface. The evaporated gallium diffused along the gold layer to form a continuous biphasic film. Moreover, this approach was compatible with traditional high‐throughput microfabrication techniques, enabling precise patterning (Figure 13b) [351]. Yu et al. also used PVD to deposit Ga‐In nanoparticles on substrates such as silicon, glass, and mica. By adjusting the deposition time, they successfully tuned the particle size, demonstrating the broad applicability of PVD in nanomaterial synthesis [352].

ECD [353] is a technique that utilizes current‐driven electrochemical reactions to deposit metals or conductive polymers from an electrolyte solutions onto substrates. By applying an external current, metal ions in the electrolyte are reduced and deposited onto the electrode surface, forming metallic thin films or conductive layers. Common electrochemical deposition techniques include electroplating and electro polymerization. For example, Chang et al. employed elastic Polybutylene Terephthalate (PBT) elastic fibers as substrates for Ni electroplating. Through surface pretreatment, the adhesion of the metal layer to the substrate was significantly enhanced, preventing the delamination of the metal layer during stretching. Ni was electroplated onto the treated PBT substrate, forming a stretchable helical metal conductor with stable performance under strain [354]. Monnens et al. demonstrated that electrodeposition enables both high‐resolution patterning of EGaIn and the formation of highly conductive, deformable circuits on PDMS elastomeric substrates. The initially deposited EGaIn formed spherical microdroplets with liquid metal cores and thin Ga_2_O_3_ shells. Subsequent mechanical sintering disrupted these oxide shells, enabling the formation of smooth, continuous liquid metal structures with enhanced conductivity [355].

Spray deposition [356] is a technique in which conductive materials, dispersed or dissolved in a liquid medium, are atomized and sprayed onto a heated flexible substrate. As the solvent evaporates, a uniform thin film of the conductive material is formed on the surface of the substrate (Figure 13c). This process uses an atomizing nozzle to disperse liquid precursors, and upon solvent evaporation, the conductive material remains as a thin, continuous layer. Spray deposition is widely used in the fabrication of conductive polymers, metal nanoparticles, and synthetic nanomaterials. It is particularly advantageous for large‐area and cost‐effective manufacturing, offering high scalability and process simplicity [357]. This technique has been applied to deposit 1D nanowire networks onto pre‐stretched elastic substrates for creating large‐area conductive circuits. For example, Kim et al. utilized spray deposition to form a random AgNW network on a pre‐stretched PDMS substrate [228]. The AgNW network adhered well to the PDMS surface, and by adjusting deposition parameters such as injection pressure, nozzle‐to‐substrate distance, and deposition duration, the network density, thickness, and morphology were precisely controlled. This optimization improved both the electrical and optical performance of the electrodes. In a similar application, spray deposition was employed to uniformly deposit CuNWs on flexible substrates, forming interconnected conductive networks with high electrical conductivity [358].

#### 3.2.2 Printing

Printing technology has been widely adopted in the fabrication of conductive circuits due to its simplicity, low cost, and high scalability. As an efficient manufacturing approach, printing enables precise patterning of conductive materials such as conductive inks or polymer solutions onto flexible substrates to form desired conductive traces [359]. Printing methods are generally divided into two main types, namely direct printing and transfer printing. Transfer printing is typically employed in applications that require high precision and complex geometries, while direct printing is more suitable for rapid and large‐scale production of conductive structures.

Direct printing refers to the process of transferring conductive materials directly onto flexible substrate surfaces. Common techniques include screen printing [360, 361, 362], inkjet printing [363, 364], and flexographic printing [365]. Screen printing employs a mesh‐based stencil to transfer conductive materials onto substrates. During this process, a squeegee forces the material through the open areas of the mesh onto the substrate surface, forming desired conductive traces. This method typically utilizes viscous inks or colloidal materials that are precisely controlled to create patterned features on substrates. Due to its simple equipment and straightforward operation, screen printing offers significant cost advantages for large‐scale production. The technique enables both high‐throughput manufacturing and continuous large‐area printing, with its most distinctive characteristic being the high aspect ratio of printed patterns compared to other printing methods [366]. However, the resolution of screen printing is limited, making it difficult to print small patterns. It is generally suitable for thicker conductive lines and larger patterns, and not suitable for fine structures. Recent advancements demonstrate that these simple strategies can produce high‐performance screen‐printed AgNW inks. Using ultra‐long AgNWs (approximately 75 µm) as conductors, researchers have achieved printed AgNW patterns with high conductivity (up to 8.32 × 10^3^ S/cm) and excellent mechanical flexibility [367]. Besides, customized stainless steel mesh masks can also be used to print graphene ink and Ag ink onto flexible PET substrates, as reported by Kim et al. [368]. Inkjet printing is a precision technique based on liquid ink droplet deposition, where ink droplets are ejected from print heads onto substrate surfaces to form desired patterns. Inkjet printing achieves high resolution and fine pattern printing by controlling the size, ejection position, and frequency of the ink droplets. Inkjet printing allows for highly accurate pattern printing, down to the micron level. This non‐contact method avoids potential substrate damage caused by contact‐based printing, making it suitable for fragile, flexible materials [369]. However, its relatively slower production speed compared to screen and flexographic printing limits large‐scale manufacturing applications [370]. For example, Huang et al. used an inkjet printing system to fabricate conductive AgNW networks on PET substrates [371]. In addition, inkjet printing can also be used to print AgNP conductors onto PI film substrates [372]. Flexographic printing employs flexible relief plates made of rubber or plastic to transfer ink onto a substrate. The ink is transferred through a blade or printing roller, and this method is suitable for continuous, high‐speed production [366]. Flexographic printing offers high efficiency and is ideal for mass production. It enables high‐throughput printing in a short time and can accommodate irregular or curved substrates. However, due to inherent limitations of the technique, flexographic printing faces challenges in producing complex and finely detailed conductive structures. Kim and co‐workers developed a nanoporous stamp‐assisted approach, where nanoparticle‐based inks are loaded onto the nanoporous stamp by dip‐coating or spin‐coating. Capillary action draws the ink into the nanopores, and upon brief contact with the target substrate, a thin ink layer is transferred, producing a patterns that precisely matches the stamp design (Figure 13d) [373].

Transfer printing technology utilizes intermediate materials to transfer patterns from an original substrate to a target substrate, and the common techniques include thermal transfer printing (Figure 13e) [374, 375] and flexible transfer printing [376]. Thermal transfer printing employs a heat source to transfer conductive patterns from a transfer film or paper onto the surface. Through heating, the thermally sensitive ink on the transfer paper comes into contact with and adheres to the target substrate, forming the desired conductive pattern. Compared to conventional methods such as photolithography, thermal transfer printing requires lower equipment costs, making it suitable for medium‐scale production. However, precise control of temperature, pressure, and transfer time is essential to ensure high fidelity and quality of the pattern. Additionally, the relatively slow speed of the transfer process limits its suitability for high‐throughput and large‐scale manufacturing. For example, Guo et al. used a desktop laser printer to print target carbon powder patterns onto thermal transfer paper. The printed paper was then placed on the target substrate and heated to 150°C for 30 s using a hot plate. This process enabled the complete transfer of the carbon powder pattern onto the substrate, forming a deformable conductor [377]. Flexible transfer printing typically utilizes elastic materials such as silicone films as intermediaries to transfer patterns from a source layer to the target substrate. This method allows for high‐precision pattern transfer on irregular or complex surfaces. However, it requires specialized flexible transfer equipment, which increases the overall equipment cost compared to more conventional printing techniques.

#### 3.2.3 Etching

Etching processes play a crucial role in fabricating conductive circuits by enabling precise patterning of conductive materials to enhance stretchability, flexibility, and electrical performance. Through etching, fine conductive line patterns can be formed on flexible substrates, commonly in designs such as serpentine or wave‐like patterns. These patterns help effectively distribute stress when the material is stretched or deformed, maintaining good electrical conductivity. Etching is also useful for selectively removing excess metal layers or exposing specific regions of conductive materials, thereby enhancing their functionality and overall performance. Etching refers to the process of removing material from a surface using chemical, physical, or optical methods. The basic procedure involves the application of specific etchants, such as acids, alkalis, or reactive gases, or the use of physical means such as laser or plasma, to remove designated areas of the target material, resulting in precise patterning or structural modification. This surface patterning technique is fundamentally divided into two main types known as dry etching and wet etching.

Dry etching, also referred to as vapor‐phase etching, is a technique that removes material from a surface using gases, plasmas, or other physical means. Unlike traditional wet etching methods that rely on chemical solutions, dry etching uses non‐liquid media to achieve material removal through chemical reactions or physical processes. Common dry etching techniques include plasma etching [378, 379] and laser etching [380], both of which are widely used for high‐precision micro‐patterning. Laser etching employs a focused high‐energy laser beam directed at the material surface. The beam induces localized heating, raising the temperature of the material to the point of evaporation or vaporization through photothermal and photochemical interactions, thereby removing unwanted regions [381]. This method allows for precise control of etching position and depth, offering high spatial resolution. The laser beam can be focused onto extremely small areas, enabling etching accuracy down to the nanometer scale. Despite these advantages, laser etching may cause a rise in surface temperature, which can lead to thermal stress or damage, particularly in thermally sensitive substrates. Furthermore, the high cost of laser equipment limits its use primarily to high‐value or specialized applications. Wang et al. used a high‐power ultra‐fast fiber laser system to laser‐etch grooves on PET substrates for conductor placement [382]. This technique enabled precise definition of the conductive pathway by directly structuring the substrate surface. Plasma etching involves the use of reactive particles, such as ions and free radicals, in a plasma state to interact with and remove material from the substrate surface. Common plasma etching techniques include reactive ion etching (RIE) and deep reactive ion etching (DRIE) [383]. By selecting appropriate process gases, the etching process can be tailored to remove materials selectively, allowing for efficient and high‐precision processing across large substrate areas. It is important to note that plasma etching requires a high‐vacuum environment, and the associated equipment is often complex and demanding in terms of maintenance. Moreover, the high‐energy ion bombardment inherent to this method may cause structural damage to brittle or sensitive materials. Vomero et al. demonstrated this technique by applying oxygen plasma RIE to pattern CF/PI composites, achieving high‐precision features for deformable conductor fabrication [384].

Wet etching [385] is a technique that utilizes liquid chemical solutions to selectively remove portions of the surface of materials. This method is straightforward to implement and involves relatively low equipment costs. However, compared to dry etching, wet etching generally offers lower precision and resolution. It is typically used for large‐area processing and coarse pattern formation. Common wet etching techniques include acid etching [386, 387], alkali etching [388], and solvent etching [389, 390]. Acid etching involves immersing the target material in an acidic solution, such as ferric chloride or hydrofluoric acid, which reacts with the material to dissolve undesired regions [391]. This method is simple and cost‐effective, and it is suited for mass production. However, it lacks the fine control that is needed for microscale patterning and may result in uneven etching depths. Additionally, the use of acidic solutions poses environmental concerns and requires proper waste management. Alkali etching employs alkaline solutions such as sodium hydroxide or potassium hydroxide to remove material, often targeting the removal of oxide layers from metals or etching of specific materials [392]. Compared to acid etching, alkaline etching offers higher material selectivity and more stable etching outcomes, making it particularly suitable for removing oxide layers. However, it generally requires longer processing times and exhibits lower overall efficiency. This process operates by dissolving specific thin films or patterned layers using a compatible solvent. It is easy to perform, low in cost, and highly efficient for tasks such as photoresist removal. Nonetheless, it offers limited material selectivity and is not suitable for applications involving complex or multilayered materials. Zhang et al. developed high‐density Cu‐based flexible transparent circuits by precisely controlling etching parameters, including solution concentration, temperature, and processing time (Figure 13f) [393]. Their work demonstrates the optimized conditions of wet etching that can achieve fine‐patterned conductive structures on flexible substrates.

#### 3.2.4 Other Fabrication Methods

While deposition, printing, and etching establish the foundational paradigms for flexible electrode fabrication, the transition toward next‐generation electronics imposes increasingly stringent requirements on patterning resolution, mechanical robustness, and the heterogeneous integration of functional materials. To address these evolving demands, a series of specialized manufacturing methodologies has been developed. By circumventing the inherent constraints of conventional processes, such as restricted material compatibility, topological limitations, and rigid environmental prerequisites. These emerging strategies unlock novel pathways for engineering flexible circuits with hyper‐stretchability, intrinsic breathability, and customized three‐dimensional architectures. Accordingly, this section highlights these frontier technologies, with a specific focus on the mechanisms and applications of laser processing, electrospinning, and microfluidic injection.

As a highly versatile and maskless alternative to conventional photolithography, laser processing, encompassing laser ablation, induction, and direct writing, has been widely employed in the fabrication of flexible and stretchable electronics. Laser ablation enables the rapid and direct patterning of complex strain‐relieving geometries without reliance on expensive photomasks. Chae et al., for example, utilized a programmable desktop laser cutter to selectively remove redundant Au/Ti layers on a PI substrate, precisely defining the desired circuit patterns without requiring complex microfabrication procedures [318]. To address the typical trade‐off between stretchability and device resolution, Yun et al. employed laser engraving to generate slanted cross‐sections on printed corrugated circuits, effectively reducing bending stiffness and guiding crack propagation to enhance macroscopic stretchability. The Au conductive layer was subsequently deposited via ion sputtering before embedding the entire structure into a PDMS matrix. The laser‐engraved grooves induce crack propagation parallel to the circuit rather than causing perpendicular fracturing, thereby allowing the conductive pathways to remain functional after crack initiation and significantly delaying catastrophic electrical failure [304]. Beyond physical ablation, laser direct writing techniques are widely used to induce localized physicochemical transformations, most notably the photothermal conversion of polymer precursors into conductive LIG. Given the mechanical mismatch often encountered when applying LIG synthesized on rigid substrates to soft tissues, several studies have focused on transferring these porous carbonaceous networks onto flexible elastomers or hydrogels. Wang et al. directly synthesized laser‐induced MXene‐decorated graphene (LIMG) on PI films coated with an MXene dispersion, which was subsequently integrated with hydrogels via a reversible freeze‐thaw method to form a 3D porous conductive network. By utilizing embedded TiO_2_ nanospheres as dynamic conductive bridges during the rupture of the graphene network under deformation, a broad linear sensing response of 0–366% was achieved [26]. To further optimize the interfacial adhesion between the laser‐patterned layer and the soft matrix, Lu et al. proposed a cryogenic transfer strategy. By flash‐freezing an ultrathin adhesive hydrogel in contact with the laser‐treated layer, they enhanced the interfacial electrostatic interactions and utilized the hydrogel as an energy dissipation layer, inducing deflected ductile fractures rather than catastrophic straight cracking. The fabricated LIG electrodes were ultimately transferred onto PPH hydrogel films via this cryogenic approach (−196°C) to yield the final flexible conductor circuits [104]. Furthermore, highly localized thermal energy from lasers allows for the selective sintering and metallization of conductive nanomaterials on heat‐sensitive flexible substrates. Kwon et al. demonstrated a low‐temperature selective laser sintering of copper nanoparticles on polymer films, this process employs ultrafast heating and cooling cycles to effectively suppress copper oxidation in air, ensuring robust electrical pathways between the nanoparticles [234]. Extending this approach to highly porous and breathable textiles, Li et al. reported a laser‐induced selective metallization strategy on PPNF. By activating specific laser sensitizers (e.g., ZnO), they achieved precise electroless copper plating, which subsequently underwent an intermetallic alloying reaction with liquid metals, enabling the selective growth of conductive pathways without damaging the heat‐sensitive fiber architectures [247]. Overall, these laser processing techniques overcome the geometric and material constraints of conventional planar fabrication, offering unprecedented design freedom for next‐generation wearable systems.

Electrospinning has emerged as a highly versatile bottom‐up fabrication method for generating continuous, high‐aspect‐ratio nanofiber networks. Compared to continuous planar films, these porous structures possess inherent breathability, vast specific surface areas, and exceptional structural flexibility, making them highly suitable for flexible conductive interfaces. To achieve conformal coverage over complex topographies, intrinsically conductive polymers can be directly electrospun into continuous networks. Bessaire et al. co‐electrospun PEDOT:PSS with a polyethylene oxide (PEO) carrier, followed by a solvent extraction process to remove the insulating PEO phase. This morphological engineering yielded highly transparent and conductive coatings capable of seamlessly conforming to and wrapping around various intricate micro‐topographies like a soft net, ensuring uninterrupted electrical current across physical obstacles [271]. Intrinsic conductivity can also be realized through the thermal conversion of polymer precursors. Wei et al. converted electrospun PAN membranes into CNFs via a high‐temperature carbonization process. The resulting graphitized skeleton provided abundant active sites and facilitated electron transfer, enabling its use as a highly efficient flexible working electrode [202]. In addition to intrinsically conductive fibers, insulating electrospun templates are frequently surface‐modified to construct robust core‐shell conductive architectures while retaining breathability. For instance, Horev et al. covalently grafted PANI onto electrospun elastomeric SIS meshes via dynamic interfacial polymerization. The nanofibrous mesh architecture endowed the devices with excellent gas permeability (10‐33 mg/h), significantly enhancing wearing comfort and effectively mitigating the skin erythema and inflammation commonly associated with the long‐term application of flexible electronics [63]. To further optimize directional sensitivity, Wang et al. fabricated highly oriented PI nanofiber arrays. This parallel nanofiber grid, formed via electrospinning, exhibited exceptional uniformity, providing an ideal template for the subsequent homogeneous deposition of silver nanoparticles [42]. Moreover, to alleviate the external abrasion of bare metal coatings, embedding these electrospun meshes within elastomeric matrices has proven to be a highly effective strategy. Singh et al. fabricated AgNFs using conventional electrospinning combined with thermal vacuum deposition, which were subsequently embedded between a PEDOT:PSS layer and a photopolymer substrate. Through a reverse peel‐transfer process, they obtained an ultrasmooth, junction‐fused conductive network that maintained reliable capacitive performance even under severe bending deformations [66].

Encapsulating room‐temperature liquid metals within elastomeric microchannels is a promising and reliable strategy for constructing intrinsically stretchable conductors. Unlike conventional solid metal films, which inevitably suffer from stress concentration and microcracking under continuous deformation, microfluidic‐templated liquid metals sustain stable electrical conductivity through the dynamic redistribution of internal fluids. Wang et al., for example, injected eutectic liquid metals into Ecoflex microfluidic channels with continuously varying cross‐sections, deliberately introducing a trapezoidal gradient geometry rather than uniform channels. Consequently, upon the application of localized pressure, the volumetric deformation of the LM channel induces a resistance variation uniquely correlated with the specific contact location. By arranging multiple gradient microfluidic conductors in parallel and employing a backpropagation neural network to process their anisotropic resistance changes, this structural design successfully decoupled the magnitude, location, and direction of the applied force without requiring a bulky cross‐grid electrode array [224].

Fundamentally, the evolution of these patterning methods represents a critical transition from conventional planar processing to function‐driven, multidimensional fabrication. The maskless prototyping capabilities of laser direct writing, the inherent breathability of electrospun meshes, and the mechanical resilience afforded by molded microfluidic architectures collectively address the complex electromechanical requirements of flexible conductors. By overcoming the rigidity and geometric limitations inherent in traditional deposition and etching processes, these advanced manufacturing technologies present new possibilities for the fabrication of highly flexible and morphologically customizable electrodes.

## 4 System and Applications

As research on deformable conductors and stretchable conductive circuits advances, the field of flexible electronics is witnessing a shift toward more integrated, intelligent, and application‐oriented systems. This progress is underpinned by synergistic developments across signal processing, data‐driven optimization, and adaptive system architectures. The transition from individual deformable conductors to functional flexible electronics necessitates a systematic approach to manage the entire signal‐to‐application workflow. To illustrate this evolution, this section follows a progressive logic, starting from the physical transmission of signals, moving to intelligent data processing via algorithms, followed by the architectural integration of hardware modules, and finally demonstrating practical system‐level applications. By examining the interaction of these technologies, we aim to illustrate how they collectively improve the performance, stability, and adaptability of flexible electronic systems, ultimately offering more versatile and efficient solutions for future intelligent wearable devices and environmentally adaptive applications.

### 4.1 Signal Transmission and Processing

As the primary interface of an adaptive conductive system, the signal transmission layer acts as the physical foundation, ensuring that raw data acquired by sensors can be delivered with high fidelity despite mechanical deformations. The application of adaptive conductive systems in flexible electronic devices continues to expand, with the stability and efficiency of signal transmission remaining a key technical challenge. Previous sections have outlined strategies for preserving conduction characteristics under large mechanical strain. Ensuring the structural integrity of the conductive paths is critical for maintaining reliable and stable signal transmission. In wearable electronics and biological monitoring systems, low‐power signal transmission is essential for achieving long‐term operational stability. Communication protocols such as Bluetooth Low Energy (BLE) [395] and Near Field Communication (NFC) [396, 397] have been widely adopted in flexible electronic systems due to their low energy consumption and high transmission efficiency. Traditional rigid circuits rely on metal wires for signal transmission, whereas deformable circuits require dynamically stable conductive paths on flexible substrates. For example, liquid metal‐elastomer composite conductors that encapsulate eutectic EGaIn alloy within microfluidic channels can maintain resistance variations of less than 5% under 300% strain, thereby ensuring stable signal transmission and providing a reliable medium for wireless signal transfer [224]. Liquid metal conductors, due to their excellent flexibility and self‐healing properties, provide a more advantageous medium for wireless signal transmission in flexible electronic devices compared to traditional rigid conductors. This type of conductor has been successfully integrated into BLE modules for real‐time ECG data transmission, with power consumption only one‐third that of conventional rigid circuits [398]. These protocols, by optimizing data transmission methods and reducing power consumption, not only ensure the real‐time nature of transmission but also extend the operational lifetime of devices. For example, Lyu et al. integrated a BLE module into a flexible sensor system for remotely monitoring the beating activity of cardiac organoids. The low‐power characteristics of BLE allow the system to operate for an extend period [395]. At the same time, wireless power transfer (WPT) technology provides a wireless power source for the system, eliminating the constraints of traditional batteries and improving the wearability and comfort of the devices. With the continuous optimization of WPT systems, such as the introduction of electromagnetic coupling technology, the efficiency and stability of wireless energy transmission have been improved, especially in dynamic environments. This provides greater flexibility and reliability for signal transmission and energy supply [399].

The deep Integration of edge computing and adaptive conductive systems represents a significant shift in flexible electronics, transitioning from a traditional “sensing‐transmission” model toward a more advanced “sensing‐decision” framework. To meet the requirements of real‐time signal processing and reduce reliance on cloud computing, edge computing has emerged as an effective solution. In conventional cloud‐based architectures, signal transmission delays and bandwidth constrains in dynamic environments [400]. By relocating data processing tasks to devices situated closer to the data source, edge computing minimizes transmission latency and enhances both processing speed and accuracy [401]. Moreover, it supports essential real‐time signal processing functions such as filtering, denoising, and feature extraction, which are critical for maintaining signal integrity. When deformation introduces signal fluctuations, edge computing can dynamically adjust the operating parameters of the system in real time, thereby mitigating signal loss or distortion caused by environmental disturbance.

With the advancement of multimodal signal transmission technologies, systems based on adaptive conductive systems are no longer restricted to a single mode of signal transmission. The integration of radio frequency (RF) technologies [402], optical signals [403], and acoustic wave signals [397] not only enhances transmission bandwidth but also enables the selection of optimal transmission modalities according to specific application scenarios and environmental conditions. For instance, Zhang et al. improved the efficiency of RF power transmission by optimizing the design of silver nanoflower (AgNF) coils, particularly under tensile strain [397]. The key to RF‐based systems lies in maintaining stable RF performance under varying strain conditions, which requires careful modulation of inductance and resistance. In frequency‐modulated signal transmission, audio signals can be transmitted through stretchable helical coils. The study investigated the effect of tensile strain on signal quality and confirmed that harmonic components of audio signals, such as piano tones or human voice, could still be detected under different strain levels. A helical structure composed of AgNWs embedded within a PDMS substrate was able to maintain reliable communication at the 2.4 GHz frequency band under 50% strain, while optical signals were simultaneously utilized to assist with data calibration. For optical signal transmission, the photosensitive components within the material absorb photons and generate electron‐hole pairs. These free charge carriers migrate toward the electrodes under the influence of an electric field, thereby producing an electrical current and enabling signal transmission [404]. While RF technologies are well‐suited for long‐distance data transmission, optical signals offer higher transmission rates and lower latency in localized regions, providing greater flexibility in signal delivery. When combined with low‐power transmission protocols, this multimodal transmission strategy can effectively address the diverse requirements of flexible electronics across various applications scenarios, enhancing both the system reliability and adaptability of the overall system.

Therefore, with the continued advancement of emerging technologies such as multimodal signal transmission, edge computing, and wireless power transfer, flexible electronic devices based on adaptive conductive systems, are expected to exhibit improved adaptability and reliability in future applications. The integration of these technologies improves the efficiency of signal transmission as well as ensures the stability of devices under dynamic conditions in complex environments. These developments provide a solid foundation for the large‐scale application of flexible electronic system.

### 4.2 Machine Learning and Algorithms

Adaptive conductive systems are capable of collecting biosignals, environmental data, or user behavior information in real time. However, the acquired data are often highly complex and prone to noise. In addition, deformation of the device or changes in external conditions can further degrade data quality, resulting in significant fluctuations in signal stability and accuracy. While the efficient transmission protocols discussed in the previous section ensure that data reaches the processing unit, they do not inherently resolve the signal distortions caused by mechanical strain. Consequently, to transform these complex and unstable data streams into meaningful information, machine learning algorithms are integrated as the intelligent core of the system to perform advanced feature extraction and pattern recognition. In recent years, the development of machine learning algorithms, especially the progress in deep learning, has provided effective tools for identifying meaningful features within complex signals and enabling accurate analysis [405].

When signals are influenced by stretching or bending of the device, trained models can be employed to automatically recognize and classify the signals of ECG, strain, motion, etc. [406]. These signals often exhibit complex time‐varying and nonlinear characteristics, especially when the sensors are deformed or exposed to external disturbances, which may result in fluctuations in signal quality and stability. Traditional classification methods, such as support vector machines (SVM) [407] and linear discriminant analysis (LDA) [408], may be inadequate for capturing the underlying complexity of these signals. Consequently, more advanced machine learning algorithms are required to address these challenges effectively [409]. Convolutional neural networks (CNNs), [410] a widely used deep learning model, have been widely used for time‐series signal classification. The hierarchical architecture of CNNs enables automatic extraction of local signal features, proving especially capable for processing intricate bioelectrical and strain signals. These networks can effectively identify distinguishing features within these signals, enhancing the accuracy of classification models. For example, Kwon et al. developed a deep learning‐embedded electrophysiological mapping system for real‐time classification of finger motions. By applying CNNs to process multi‐channel electromyography (EMG) data, the system achieved precise classification across seven types of finger movements with an accuracy of approximately 99% [411]. This result demonstrates the significant potential of deep learning in classifying complex bioelectrical signals, particularly under dynamic conditions. Meanwhile, recurrent neural networks (RNNs) and their variants, such as long short‐term memory (LSTM) networks [412] and gated recurrent units (GRUs) [413], have demonstrated strong capabilities in handling time‐series data by capturing long‐term temporal dependencies. In bioelectrical signal and motion data classification tasks, LSTM networks can learn the dynamic characteristics of signals over time, overcoming the limitations of traditional methods in processing long time‐series signals. Kim et al. developed a conductive circuit incorporating curved serpentine structures, employing LSTM for processing time‐series signal data. The LSTM effectively captured the temporal characteristics of sensor signals, particularly in analyzing dynamic finger movements. Through time‐series data processing, the system accurately identified finger flexion and extension states, achieving an average classification accuracy of 96.2% [340]. This research demonstrates that the application of LSTM for temporal signal analysis not only enhances classification precision but also expands the potential applications of adaptive conductive systems in dynamic scenarios. In the Intelligent Takeover Assistance System (ITAS), Lu et al. applied a long short‐term memory (LSTM) algorithm to the non‐driving behavior recognition module. The primary objective of this module was to identify drivers’ non‐driving behaviors by analyzing electrical signals generated from omnidirectional sensing gloves (AS‐Gloves). The use of LSTM was motivated by its strong capability in handling time‐series data, which is essential since these electrical signals exhibit temporal dependencies that evolve with driver behavior. LSTM networks, with their memory cells and gating mechanisms, are particularly effective in capturing long‐term dependencies within sequential data, enabling accurate identification of complex behavioral patterns. To further enhance performance, the authors proposed a hybrid model that combines a time‐distributed convolutional neural network (CNN) with LSTM, referred to as the TCNN‐LSTM architecture. In this framework, the CNN component extracts spatial features from the input signals, while the LSTM component captures the temporal dynamics. This integration allows for effective recognition of non‐driving behaviors by jointly modeling spatial and temporal characteristics of the input data (Figure 14a) [414]. Beyond basic data processing, advanced deep learning algorithms such as RNNs and LSTMs can extract underlying temporal relationships from continuous physiological signals. This capability enables predictive functionality, potentially providing early warning systems for critical events.

**FIGURE 14:**
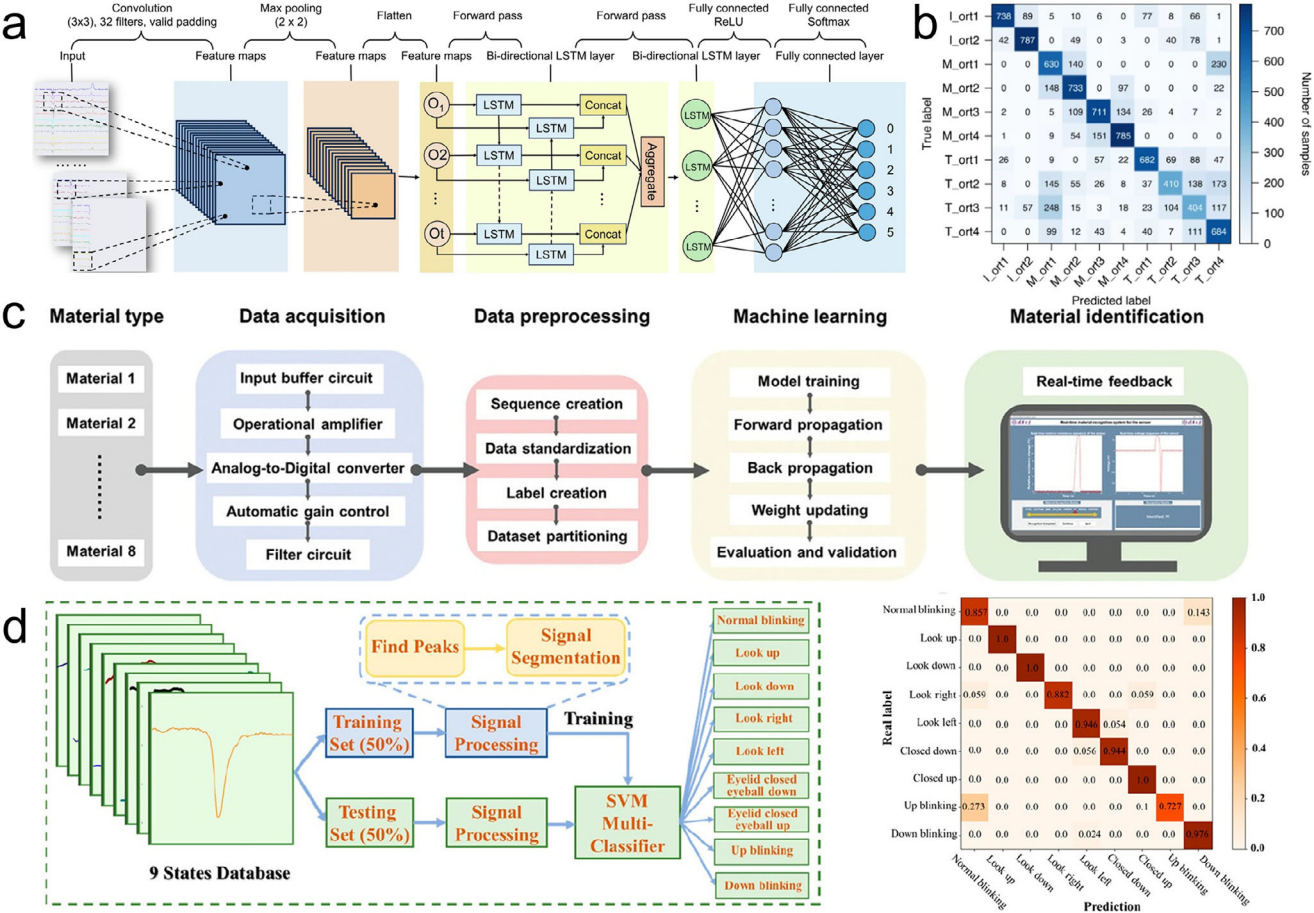
Schematic diagram of machine learning architectures in flexible conductor systems. (a) Structure of detailed TCNN‐LSTM model. Reproduced with permission [414]. Copyright 2025, Springer Nature. (b) The confusion matrix for classifying the letter and the orientation. Reproduced with permission [415]. Copyright 2021, Springer Nature. (c) Machine learning process flowchart. Reproduced with permission [417]. Copyright 2025, Wiley‐VCH. (d) Flow chart of a machine learning algorithm for eye movement classification. Confusion matrix of classification training recognition accuracy. Reproduced with permission [418]. Copyright 2022, American Chemical Society.

In addition to the appropriate algorithm and dataset selection (Figure 14b) [415], the significance of data processing also lies in effective feature extraction [416]. Time‐domain and frequency‐domain features represent fundamental characteristics in traditional signal processing, providing essential information about morphology and spectral composition. For biological and strain signals acquired by adaptive conductive systems, time‐domain features including mean values, variance, and peak amplitudes effectively characterize basic signal variations, while frequency‐domain features such as power spectral density reveal periodic components and frequency characteristics. For non‐stationary signals, wavelet transform and time‐frequency analysis can accurately capture transient changes, and separate noise from relevant information, and provide cleaner and more informative inputs for classification models. These methods enable precise identification of instantaneous signal properties, thereby providing more accurate inputs for classification models. In a real‐time material recognition system based on triboelectric and piezoresistive signals, Xue et al. utilized a one‐dimensional convolutional neural network (1D‐CNN) to classify deformable materials. A key component of their approach was time‐domain signal analysis, which served as the foundation for signal processing and feature extraction. Triboelectric and piezoresistive signals generated during material contact and separation are inherently time‐varying. Capturing these dynamic patterns is crucial for accurate material identification. Time‐domain analysis was used to retain the temporal characteristics of the raw signals, such as amplitude fluctuations, rise time, and waveform shape—features that directly reflect the physical interactions between materials. Unlike frequency‐domain methods that focus on spectral components, time‐domain analysis processes the signal in its original form, making it particularly effective for detecting transient behaviors during material deformation. The extracted time‐domain features were then fed into the 1D‐CNN model, which automatically learned and classified the underlying patterns without requiring manual feature engineering. This model demonstrated strong adaptability across different signal types and enhanced the system's capability to accurately identify various materials in real time (Figure 14c) [417]. Through time‐domain analysis, features including amplitude fluctuations and rise time were extracted to characterize the instantaneous variations and dynamic behavior of finger movements. These parameters effectively reflected the transient responses during motion. In this way, the system was able to identify and distinguish gestures or motion patterns at different time points. Additionally, frequency‐domain features such as frequency peaks, bandwidth, and energy distribution regions also served as key input features for the system. For example, in the study on eye movement signals, Xu et al. employed time‐domain analysis to reveal a linear relationship between the amplitude of the EOG signal and the angle of eye movement, highlighting the dynamic characteristics of signal amplitude during eye motion. In addition, by setting appropriate thresholds, they segmented continuous periodic eye movement signals to extract single‐cycle segments, thereby providing standardized inputs for subsequent feature extraction and classification. This processing approach demonstrates the practical value of time‐domain analysis in signal preprocessing (Figure 14d) [418].

In summary, machine learning provides robust technical support for adaptive conductive systems, particularly in areas such as signal recognition, data processing, and intelligent decision‐making. As algorithms continue to evolve and hardware systems are further optimized, machine learning is anticipated to play an increasingly significant role in the development of flexible electronics, which is expected to accelerate innovation and drive flexible electronic technologies toward more advanced and sophisticated applications.

### 4.3 System Integration

Beyond optimizing signal transmission and processing algorithms, the practical realization of a flexible system relies on system integration, which involves the physical orchestration of sensors, processors, and power management units into a unified platform. One of the primary challenges in system integration is achieving the efficient incorporation of these functional modules while preserving mechanical flexibility and structural stability. In addition, certain components must meet the concurrent requirements of miniaturization and operational reliability [416]. Although each module performs distinct functions, they are closely coordinated to form a unified, reliable, and high‐performance system (Figure 15a) [395].

**FIGURE 15:**
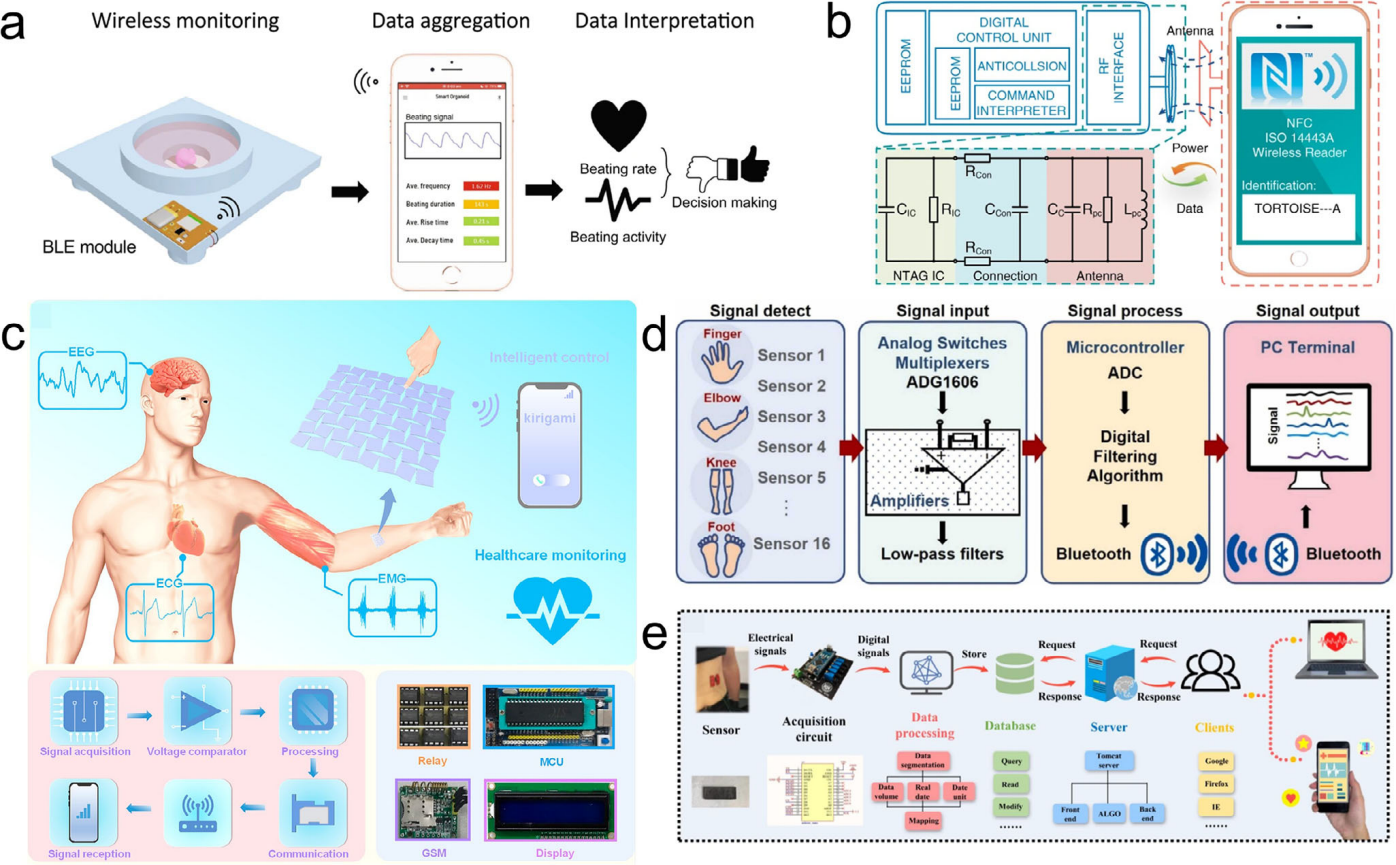
Schematic diagram of the integration architecture of adaptive conductive systems. (a) Full spectrum procedures of organoid biosensory system integrated with wireless BLE module for remote organoid monitoring and decision‐making. Reproduced with permission [395]. Copyright 2022, Springer Nature. (b) Functional block diagram of near‐field communication (NFC) tags with a smartphone that includes an NFC reader. Reproduced with permission [397]. Copyright 2020, Springer Nature. (c) A smart dialing communication system, which is applied to human skin to call a cellphone. Reproduced with permission [68]. Copyright 2022, American Chemical Society. (d) Schematic illustration of the system to monitor health signals. Reproduced with permission [71]. Copyright 2023, Elsevier. (e) Schematic illustration of a big data cloud monitoring platform. Reproduced with permission [187]. Copyright 2022, Springer Nature.

The sensor module serves as the core component of the system (Figure 15b) [397], responsible for acquiring environmental or physiological signals. To meet the requirements of adaptive conductive systems, these sensors must exhibit high sensitivity, broad measurement ranges, and excellent mechanical flexibility [419]. Recent advancements in flexible electronics have enabled widespread application of sensors based on conductive polymers, metal nanowires, CNTs, and other novel materials. These materials maintain stable electrical performance under various mechanical deformations, including stretching, bending, and compression, thereby ensuring accurate signal acquisition. In biological signal monitoring applications, flexible strain sensors, temperature sensors, and pressure sensors find extensive use across multiple domains [420]. These include but are not limited to ECG monitoring, EMG recording, respiratory rate measurement, and motion tracking. The unique properties of adaptive conductive systems allow these sensors to conform to biological surfaces while maintaining signal fidelity during movement and deformation. Gao et al. integrated multiple sensor modules onto a FPCB, with each module specifically designed to detect distinct biomarkers [398]. To ensure high‐quality signal acquisition, sensor designs must incorporate appropriate sensitivity levels and effective noise suppression. Additionally, efficient sensing relies on optimizing the layout of sensors and interconnects through integration strategies that accommodate various deformations while minimizing signal interference. The design of the sensor module should also be compatible with other functional components to facilitate system integration.

After signal acquisition, the data processing module converts raw sensor data into operational information (Figure 15c) [68]. The data processing module typically comprises a microprocessor, memory units, and signal processing components (Figure 15d) [71], which perform tasks such as filtering, decoding, and data analysis [421]. In flexible electronic systems, the processing module must achieve a balance between high integration and low power consumption, while maintaining robust computational capacity and adaptability [422]. A primary challenge lies in extracting meaningful information from complex signals under dynamic, high‐noise conditions and ensuring real‐time responsiveness. With the applications of flexible electronics, microprocessors and memory modules based on flexible integrated circuits are rapidly evolving, enabling the miniaturization of intelligent sensing systems without compromising computational efficiency. In addition to real‐time data processing, the module must also support data compression and efficient energy utilization to extend the overall operational lifespan of the system.

The energy management module is another essential component, responsible for delivering continuous power to support the stable operation of adaptive conductive systems. In these systems, flexibility and miniaturization of the power supply are particularly important. Conventional rigid batteries often do not meet the requirements of flexible electronics. As a result, flexible batteries and energy harvesting technologies have become key areas of this research. For example, integrated power solutions that incorporate rechargeable lithium polymer batteries [423], supercapacitors, and energy harvesting techniques [424] can provide a reliable power supply, especially in continuous monitoring applications such as physiological signal tracking and motion detection. The energy harvesting devices convert ambient energy sources such as light, heat, and mechanical motion into electrical energy, enabling continuous system operation. After energy generation, an efficient energy storage module is essential to retain and buffer the harvested energy. Subsequently, the energy management module must support efficient energy conversion and distribution to ensure the stable operation of all system components under various working conditions [425].

The output interface module is responsible for presenting processed data in a visual or interactive form to the user (Figure 15e) [187]. In adaptive conductive systems, the output interface must not only support data visualization but also enable effective user interaction. Flexible display technologies offer high‐quality visual output while preserving user comfort and convenience [426]. In systems requiring real‐time feedback and control, the output interface module should incorporate touch functionality to support intuitive user operations and timely adjustments. Furthermore, the design of the interface module facilitates the seamless integration with other functional modules, ensuring accurate data transmission and prompt system responsiveness [427].

In summary, the integration design of adaptive conductive systems requires not only functional optimization of individual modules but also highly coordination integration among all components. Each module must operate in close alignment with the others to ensure overall system performance, reliability, and stability.

### 4.4 Applications

Empowered by stable transmission, intelligent algorithms, and robust integration strategies, adaptive conductive systems have moved beyond fundamental research into diverse practical applications. By offering a compelling combination of flexibility, stretchability, and conductivity, these integrated systems are uniquely well‐suited for dynamic environments where conventional rigid electronics often fail. Notably, electronic systems based on adaptive conductive systems combine high electrical conductivity with mechanical adaptability, providing a critical foundation for the next generation of flexible electronic technologies. This technical synergy has fostered prosperous development across multiple application domains, ranging from wearable electronics and bioelectronics to intelligent perception systems and environmental monitoring. These conductors can be integrated into components such as displays, sensors, and electronic skin, etc., thereby enabling the development of fully flexible and wearable systems [428]. Translating these adaptive conductors from benchtop prototypes to practical devices hinges on application‐specific material design, as distinct operating environments impose unique physicochemical and mechanical constraints. Wearable electronics, which prioritize skin‐conformability, breathability, and non‐toxicity, typically rely on soft elastomers paired with biocompatible fillers. Implantable bioelectronics present far more stringent requirements; mitigating foreign‐body responses necessitates absolute biocompatibility, anti‐biofouling properties, and a tissue‐matching mechanical modulus, making naturally derived biopolymers or specialized biodegradable hydrogels preferable to conventional synthetic rubbers. Meanwhile, industrial and environmental monitoring shifts the focus toward mechanical robustness, cost‐effectiveness, and long‐term endurance against harsh stressors like extreme temperature fluctuations, humidity, and ultraviolet radiation. These scenarios generally utilize highly durable polymer matrices integrated with stable carbonaceous fillers [429]. Ultimately, the viability of the diverse practical applications detailed below is intrinsically tied to this targeted, environment‐driven material formulation.

Adaptive conductive systems have demonstrated broad potential in wearable electronics, especially in applications such as health monitoring [430], motion tracking, and daily activity recognition [431]. For instance, by combining conductive polymers with stretchable substrate materials, researchers have successfully developed bendable and stretchable touchscreens that respond to user gestures in real time and function reliably under complex environmental conditions. This advancement plays a pivotal role in driving innovation in smart electronic products. In a representative study, Mostafalu et al. developed an automated “electronic bandage” specifically engineered for chronic wounds. Driven by these clinical demands, they selected an alginate‐based hydrogel for its excellent capacity to manage wound exudates, paired with a flexible, FDA‐approved parylene substrate to ensure conformal adhesion to the dynamic wound bed. This application‐specific material configuration enabled the reliable, continuous monitoring of wound pH and temperature, which, when integrated with an automated drug delivery module and wireless data processing, provided a closed loop platform for early infection alerts and on‐demand treatment [432]. Wearable sensors based on flexible piezoresistive materials, such as PPy@rGA (polypyrrole/reduced graphene oxide aerogel) films, have also been used to monitor both macro‐movements (e.g., finger bending and foot pressure) and subtle physiological signals (e.g., pulse) [433]. When transitioning to multi‐modal epidermal monitoring, the integration of physically disparate sensors (e.g., rigid acoustic transducers and soft electrochemical electrodes) dictates strict material selection to ensure mechanical resilience and prevent signal crosstalk. Driven by these requirements, an epidermal patch was developed using a highly conformal styrene‐ethylene‐butylene‐styrene (SEBS) block copolymer substrate. This specific elastomeric matrix enabled the robust integration of rigid piezoelectric chips and soft printed traces via a room‐temperature solvent soldering process (Figure 16a) [434]. These devices, when fixed on the arm, neck, or insole, can detect muscle activity, swallowing behavior, and gait, and transmit motion signals to external devices such as computers for analysis via wireless modules [435]. With continuous advancements in sensor technology and deformable conductive materials, future devices will achieve tighter integration with the human body for comprehensive health monitoring. Beyond conventional vital sign tracking, next‐generation wearable systems incorporating artificial intelligence and machine learning will provide disease prevention, early diagnosis, and personalized health management capabilities.

**FIGURE 16:**
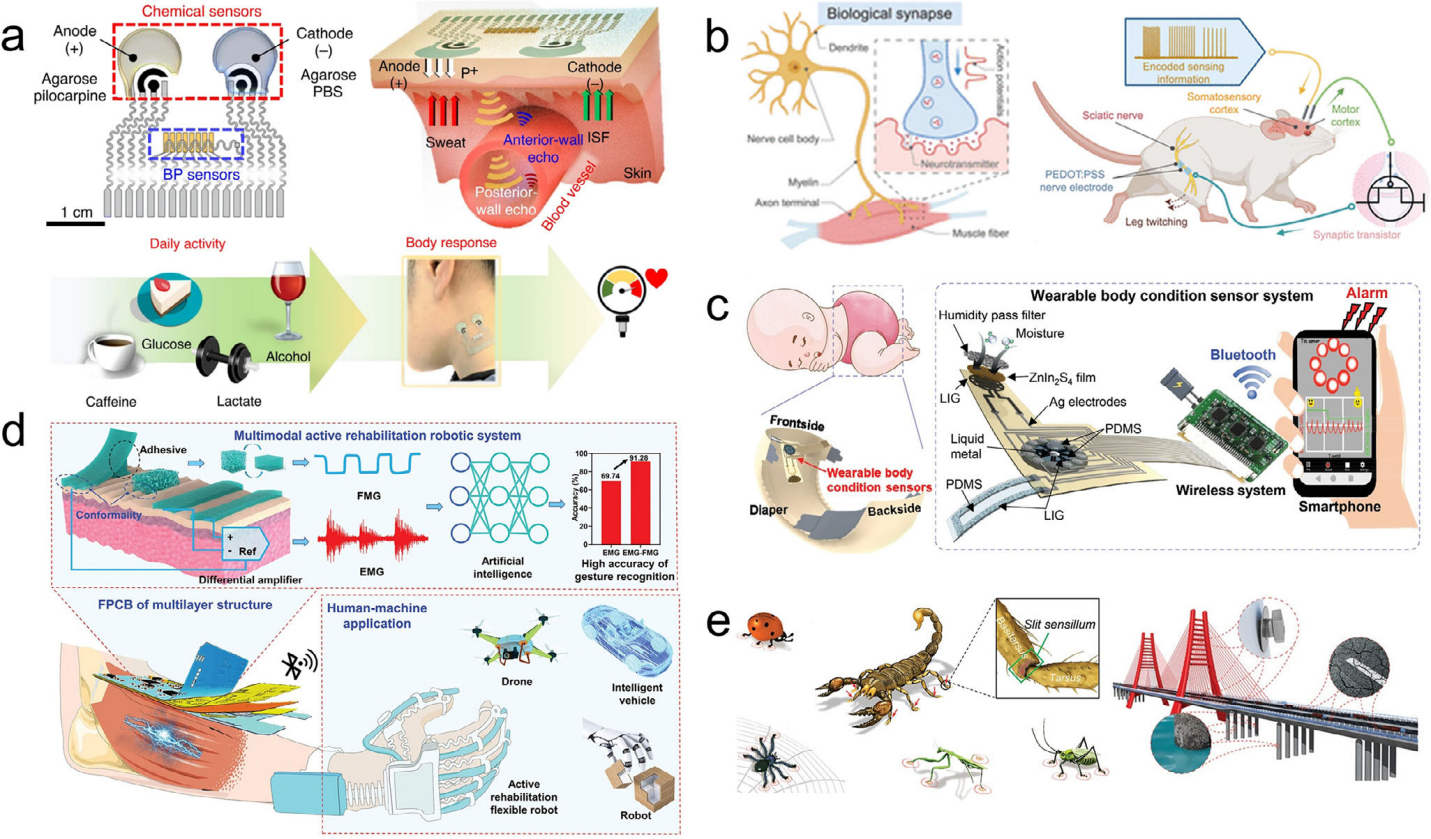
Scenario applications of adaptive conductive systems. (a) The stretchable integrated BP‐chemical sensing patch. Reproduced with permission [434]. Copyright 2021, Springer Nature. (b) A monolithic soft prosthetic electronic skin. Reproduced with permission [437]. Copyright 2023, American Association for the Advancement of Science. (c) Conceptual illustration of the multimodal flexible sensor system for wireless vulnerable population monitoring. Reproduced with permission [445]. Copyright 2021, Wiley‐VCH. d) Schematic illustration of the hydrogel‐based electromyogram and pressure sensors‐enabled wearable HMI interface and the corresponding AI‐assisted intelligent active rehabilitation robotic system. Reproduced with permission [446]. Copyright 2024, Wiley‐VCH. e) A bioinspired flexible strain sensor consisting. Reproduced with permission [429]. Copyright 2022, Wiley‐VCH.

In the biomedical field, adaptive conductive systems have demonstrated considerable promise for advancing non‐invasive diagnostics and electronic skins [436]. By bridging electronic functionalities with bio‐interactive properties, adaptive conductive systems are enabling a new generation of bioelectronic devices capable of dynamic interaction with the human body. Adaptive conductive systems have been integrated into soft electronic skin (e‐skin) systems, which mimic the sensory feedback functions of biological skin. However, direct interfacing with the peripheral nervous system dictates exceptionally stringent material criteria, particularly the necessity for safe, ultra‐low operating voltages and robust stability within physiological environments. To address these application‐specific challenges, researchers engineered a monolithically integrated e‐skin utilizing a high‐permittivity stretchable trilayer dielectric to ensure safe, low‐voltage operation. Furthermore, they deliberately selected a solid‐state polyelectrolyte matrix for the artificial synapse to prevent component dissolution in biofluids. Built upon these tailored materials, the system demonstrates reliable multimodal sensing capabilities (including pressure and temperature detection) and successfully converts stimuli into bioinspired neural pulse signals. In the practical experiment, researchers connected an e‐skin system to a live rat model to simulate the sensory‐motor feedback loop. When the sensor received external mechanical stimuli, it successfully triggered a response in the somatosensory cortex and subsequently stimulated the sciatic nerve via an artificial synapse, causing downstream muscle contractions. This illustrates the feasibility of artificial perception‐motion feedback systems (Figure 16b) [437]. Moreover, a hydrogel‐based nanocomposite composed of laser‐induced graphene (LIG) has been developed as a novel e‐skin platform for real‐time monitoring of respiratory rate, heart rate, body temperature, and skin humidity. These systems support early disease diagnosis and improve health management by providing timely and accurate physiological information [104]. Another example involves a bionic artificial skin based on an ionic gel composite. The integration of a hard scaffold, fabricated by direct ink writing (DIW), and a soft ionic gel matrix enables the device to exhibit high stretchability, ionic conductivity, self‐healing, and crack resistance, which can effectively mimic the mechanical behavior of natural skin while achieving long‐term durability [438]. Through the integration of biocompatible materials, flexible configurations, and neuromorphic signal processing, adaptive conductive systems are accelerating the development of intelligent, responsive biomedical devices.

In intelligent perception and interaction systems, adaptive conductive systems play a critical role in enabling tactile perception, real‐time feedback, and adaptive control [439, 440, 441, 442]. Unlike wearable biomonitoring devices that prioritize skin‐compatibility and softness, the material design for robotic interfaces is fundamentally dictated by the need for high mechanical toughness, fatigue resistance, and signal stability under severe physical abrasion and multidirectional shear forces. These applications are particularly prominent in intelligent robotics, where traditional rigid electronic and electrical systems fail to meet the demands for flexible morphological changes and complex motion control [7]. By integrating adaptive conductive systems into intelligent robotics and interactive human–machine interfaces, precise control can be achieved through bidirectional signal communication. For such dynamic interactive applications, material selection is fundamentally driven by the dual requirements of extreme mechanical resilience against joint movements and visual unobtrusiveness for daily wear. For instance, to replace brittle conventional conductors like indium tin oxide (ITO), researchers deliberately engineered transparent materials using patterned graphene heterostructures hybridized with AgNWs and piezoelectric PLA/SWNT composites [443]. Through the design of flexible circuits, robots can adaptively adjust like humans when performing complex tasks, thereby enhancing their intelligence and operational capabilities. For example, the flexible tactile sensor developed by Zhang et al. mounted on a robotic hand allows the robot to manipulate objects dexterously while also enabling force direction perception for intelligent indication systems [424]. In addition to robotics, tactile sensors have also been used in prosthetics and assistive technologies. Li et al. developed a multifunctional tactile sensor specifically using a PEDOT:PSS‐coated melamine sponge. The macroporous sponge skeleton provides excellent mechanical deformability for piezoresistive force sensing, while the organic PEDOT:PSS coating enables thermoelectric temperature sensing. Integrated with a machine‐learning algorithm, the resistance and open‐circuit voltage variations were processed by a signal acquisition module to trigger real‐time haptic feedback via a mobile device. Ultimately, this tailored material strategy empowers amputees to regain comprehensive, multi‐dimensional tactile perception without relying on visual cues [444]. Applications have also demonstrated capabilities in body condition monitoring, such as measuring finger pressure and temperature changes during object handling. A wearable LIG‐based sensor system, for instance, has been designed to monitor sleep posture, breathing rate, and diaper moisture. The system provides wireless alerts to caregivers, offering early warnings of conditions such as sudden infant death syndrome (SIDS) or incontinence in elderly or disabled populations (Figure 16c) [445]. In some studies, users can control these devices through hand gestures, enabling precise operation. The system utilizes hydrogels featuring skin‐like mechanical properties, biocompatibility, and cost‐effectiveness as the sensing materials. Unlike traditional rigid materials, these intrinsically stretchable hydrogels significantly enhance wearing comfort and minimize perceptual errors caused by the extrusion of the material on muscles during movement. Compared with traditional handheld remote controllers or glove‐based methods, this gesture‐based human–machine interaction approach is more flexible, natural, and convenient, enhancing both operational flexibility and user convenience (Figure 16d) [446]. The integration of adaptive conductive systems into smart systems improves mechanical adaptability as well as enhances the sensory and decision‐making capabilities of soft electronics. By combining advanced signal acquisition with machine learning algorithms, these systems can perform complex tasks with high precision and autonomy.

With the growing adoption of smart packaging and the IoT, sensors and circuits are required to possess high flexibility and adaptability to accommodate diverse application scenarios [421]. Adaptive conductive systems are increasingly utilized in fields such as food and pharmaceutical packaging, where they enable real‐time monitoring of environmental parameters including temperature, humidity, gas leakage, and product freshness. These intelligent packaging systems facilitate real‐time information transmission and dynamic data updates through embedded sensors, thereby enhancing consumer safety and improving the overall shopping experience. For example, the flexible sensors developed by Shi et al. were used to detect chlorpromazine and rhodamine B in food, chemicals that are illegally employed by some companies as food colorants despite their high toxicity and carcinogenicity [447]. Additionally, Wang and colleagues fabricated sensors for detecting p‐nitrophenol (p‐NP), a hazardous pollutant commonly used in dyes, textiles, and pesticides, which poses serious health risks to humans and animals [448]. In the field of smart packaging, Zhan et al. fabricated a self‐healing deformable conductor based on TPU, forming a self‐repairable flexible hybrid electronic system to monitor temperature and humidity in storage environments. This innovation holds great potential for applications in food preservation packaging [449]. Environmental monitoring and disaster early warning systems have also benefited from innovations in adaptive conductive systems. In recent years, frequent global climate changes and natural disasters have made it imperative to develop efficient, low‐cost sensing systems for environmental data collection. Deformable conductor‐based environmental sensors can be deployed on a large scale across various environments, including water quality monitoring, air pollution detection, and soil moisture assessment [421]. These sensors not only enable real‐time monitoring of natural resource changes but can also be integrated with smart city infrastructures and disaster warning systems. Through data transmission and cloud computing analysis, they provide real‐time decision support to help prevent environmental crises. For example, the flexible sensors developed by Liu et al. can detect minute mechanical signals from any direction, including subtle vibrations, cracks, and corrosion in buildings. By identifying vibration signals from different directions, it quickly locates potential failure sources such as cracks and loose screws, which is crucial for the structural safety of buildings and bridges. In a specific implementation, sensors based on CNT/PDMS composites were embedded into concrete cracks to provide early warnings of material fatigue. Resistance‐based measurements from these sensors exhibited an accuracy approximately five times higher than that of conventional strain gauges, thereby substantially enhancing the effectiveness of safety monitoring and predictive maintenance efforts (Figure 16e) [429].

Overall, ongoing research and expanding applications of adaptive conductive systems are increasing their importance across multiple fields. Crucially, the successful deployment of these technologies fundamentally relies on application‐driven material selection [450]. In intelligent electronics, adaptive conductive systems enable the development of integrated systems that combine flexible displays with real‐time sensing capabilities, forming multifunctional platforms for interactive visualization, physiological monitoring, and feedback control. In soft robotics and human–machine interaction, they provide crucial circuit infrastructure for precise actuation and adaptive motion control. Furthermore, in environmental monitoring and disaster early warning systems, the excellent integrability and high sensing sensitivity of adaptive conductive systems make them ideal for constructing large‐scale, cost‐effective sensing platforms.

## 5 Conclusion and Outlook

With the rapid development of deformable electronics, adaptive conductive systems have emerged as a foundational technology, demonstrating tremendous potential in wearable bioelectronics, intelligent prosthetics, soft robotics, and environmental monitoring. This review systematically summarizes the recent progress in the field, encompassing the synergistic development of material systems (including various deformable substrates and conductive materials), circuit interconnection designs, scalable fabrication techniques, and intelligent system‐level integration.

Despite the significant progress achieved in laboratory settings, the successful translation of these technologies into practical, large‐scale applications is still impeded by several fundamental and engineering bottlenecks. A primary challenge remains the inherent trade‐off between electrical conductivity and mechanical stretchability, particularly under extreme or cyclic loading. At the material level, the long‐term operational reliability of these conductors is often compromised by structural and chemical degradation. For instance, carbon‐based composites frequently suffer from weak interfacial bonding between the conductive fillers and the polymer matrix, while metallic nanomaterials are highly susceptible to oxidation that severely degrades continuous conductivity. Future research should prioritize advanced interfacial engineering and molecular design to overcome these limitations. The incorporation of dynamic reversible bonds, such as supramolecular interactions or dynamic covalent crosslinks, into polymer networks offers a promising route to realize intrinsic self‐healing capabilities, allowing the spontaneous reconstruction of damaged conductive pathways. Concurrently, developing sophisticated core‐shell nanostructures or robust encapsulation strategies at the molecular level will be essential to preserve the chemical stability of metallic conductors in harsh environments.

Beyond material synthesis, scalable manufacturing and system‐level integration present substantial hurdles. Currently, the mass fabrication yield for high‐resolution deformable circuits is often below 70%. This limitation is largely attributed to insufficient precision in micro/nano‐scale processing and the inevitable interfacial failure, or delamination, that occurs when integrating mechanically mismatched materials. Bridging the gap between benchtop prototyping and industrial commercialization will require a shift toward continuous, high‐throughput manufacturing methodologies. Techniques such as multi‐material roll‐to‐roll processing and high‐resolution direct ink writing must be further refined to ensure uniform quality over large areas.

From the application perspective, the future trajectory of adaptive electronics will therefore increasingly depend on the deep convergence of adaptive hardware and artificial intelligence. Research should be directed toward hardware‐software co‐design, particularly the development of “in sensor computing” and “edge‐AI” architectures. By embedding lightweight machine learning algorithms directly within flexible sensor nodes, multimodal signals can be locally processed, decoupled, and denoised in real time. This integration will significantly reduce data transmission burdens and enable truly autonomous, closed‐loop smart systems.

As the deployment of flexible electronics scales up, the environmental impact of accumulating electronic waste cannot be overlooked. Future material designs must increasingly prioritize sustainability through the development of transient, biodegradable, and recyclable conductive systems. Minimizing the environmental impact of these devices necessitates the adoption of naturally derived biopolymers and sustainable fabrication methods. Furthermore, for long‐term implantable devices, mitigating immune rejection and the foreign body response remains highly dependent on robust biocompatibility and precise modulus matching with host tissues.

Standing on the brink of this technological breakthrough, the evolution of adaptive conductive systems represents a paradigm shift that extends far beyond traditional materials science. The transition from the relentless pursuit of smaller and faster rigid silicon‐based chips to the development of softer, more resilient, and tissue‐compliant electronics provides a new trajectory that transcends conventional physical constraints. Ultimately, the continued progress of this field will rely heavily on sustained interdisciplinary convergence. The seamless integration of materials science, electrical engineering, biomedical engineering, and computer science is expected to generate innovative solutions that overcome existing bottlenecks, paving the way for a new generation of intelligent electronics that fundamentally redefine human–machine interfaces and bio‐integrated systems.

## Author Contributions

**Jingyi Niu**: writing – review and editing. **Jie Chen**: writing – review and editing. **Shuo Zhang**: writing – review and editing. **Siyu Liang**: writing – original draft. **Hongjian Zhang**: funding acquisition, methodology, project administration, validation, writing – review and editing. **Yufei Lu**: writing – original draft, writing – review and editing. **Wei Huang**: methodology, project administration, resources, supervision. **Dongsheng Jia**: project administration, writing – review and editing, supervision.

## Conflicts of Interest

The authors declare no conflicts of interest.
